# HADD: A hierarchy-aware disentangled diffusion framework with spatio-temporal denoising for 3D human pose estimation

**DOI:** 10.1371/journal.pone.0359321

**Published:** 2026-09-28

**Authors:** Jinze Jiang, Zhongheng Jian, Hualing Zheng, Minsi Liang, Yuxing Shi, Liming Zhang

**Affiliations:** 1 Department of Physical Education, Fujian Agriculture and Forestry University, Fuzhou, China; 2 College of Intelligent Science and Technology, Inner Mongolia University of Technology, Huhhot, China; 3 Department of Physical Education, Fujian University of Technology, Fuzhou, China; 4 School of Electrical and Electronic Engineering, Nanyang Technological University, Singapore, Singapore; 5 School of Software Engineering, Sichuan Polytechnic University, Deyang, China; 6 Department of Mechanical Engineering, Hebei Petroleum University of Technology, Chengde, China; Georgia State University, UNITED STATES OF AMERICA

## Abstract

Monocular 3D Human Pose Estimation (3D HPE) is a core task in computer vision, which provides critical technical support for applications including VR/AR, immersive human-computer interaction, digital human animation, as well as sports motion capture, athletic technique diagnosis, physical fitness assessment and sports rehabilitation training. However, existing methods still face three key limitations: diffusion-based methods lack explicit human anatomical prior constraints in the diffusion process; disentangled-based methods suffer from severe hierarchical error accumulation along the skeletal kinematic tree; and most mainstream methods fail to fully mine the fine-grained hierarchical spatio-temporal correlations of the human skeleton, leading to increased estimation error for high-hierarchy joints. To address these challenges, we propose a novel Hierarchy-Aware Disentangled Diffusion framework with Spatio-Temporal Denoising (HADD) for monocular 3D HPE, which deeply integrates disentanglement strategy with the diffusion model and embeds skeletal hierarchical information into the full diffusion pipeline. In the forward diffusion process, HADD disentangles 3D pose into bone length and bone direction features, and injects Gaussian noise into the two features separately. In the reverse denoising process, we design a Hierarchical Spatial and Temporal Denoising (HSTD) module to accurately model the hierarchical spatio-temporal relationships of the human skeleton and alleviate error amplification of high-hierarchy joints. Meanwhile, a hybrid loss function combining 3D disentanglement loss and 3D pose loss is constructed to realize dual supervision at the bone and joint levels. Extensive experiments on Human3.6M and MPI-INF-3DHP datasets show that HADD achieves consistent performance improvement over state-of-the-art methods. On Human3.6M, it reduces average MPJPE by up to 12.9% compared with traditional disentangled-based methods, and outperforms mainstream diffusion-based probabilistic methods under lightweight inference configurations. On MPI-INF-3DHP, HADD achieves an MPJPE of 29.2 mm, a PCK of 98.5%, and an AUC of 78.1 under single-hypothesis inference, reaching new state-of-the-art performance on this dataset under the single-hypothesis inference setting with only ground-truth 2D joint sequences as input. This work provides an effective solution to the core limitations of existing 3D HPE methods, and also offers reliable technical support for fine-grained motion analysis and quantitative assessment in sports training and rehabilitation scenarios.

## 1. Introduction

3D Human Pose Estimation (3D HPE) is a core research direction in the field of computer vision, with its primary goal being to accurately regress the 3D coordinates of human joints in 3D space from 2D human pose sequences input via monocular vision. This technology provides critical technical support for a wide spectrum of practical applications, including virtual and augmented reality (VR/AR) [1], immersive human-computer interaction [2], digital human animation [3], intelligent video surveillance [4], as well as competitive sports motion capture, athletic technique diagnosis and optimization and public physical fitness assessment. In these scenarios, the precise capture and analysis of human 3D skeletal motion can realize realistic real-time driving of virtual avatars, construction of natural non-contact interactive systems, fine-grained recognition of complex human actions, quantitative assessment of motor function in rehabilitation scenarios, and standardized quantitative evaluation of sports technical movements. Especially in competitive sports training and youth fitness testing, 3D pose estimation breaks through the limitations of traditional manual observation and 2D plane analysis, enabling three-dimensional refined analysis of dynamic movements such as gymnastics, track and field, racket sports and martial arts, assisting coaches in accurately identifying technical defects, formulating personalized training schemes, and effectively reducing the incidence of sports injuries. Current mainstream 3D HPE methods [5,6] generally adopt a two-stage processing strategy: (1) first, high-precision 2D joint coordinates are detected from raw RGB images using mature 2D human pose estimation algorithms; (2) then, a 2D-to-3D lifting operation is performed to compensate for the lack of depth dimension information, ultimately yielding the estimation results of 3D human poses.

In recent years, monocular 3D HPE technology has witnessed rapid development, and researchers have proposed a variety of solutions to address the core challenge of depth ambiguity, which can be mainly divided into two research veins. (1) The first is the spatiotemporal information mining direction: such methods leverage Convolutional Neural Networks (CNNs) [7] or Transformer architectures [8] to fully explore temporal information and spatial context information in video sequences, thereby compensating for the depth information loss in the 2D-to-3D mapping. For example, Bian et al. [9] proposed a novel human pose estimation framework that adopts a task-specific backbone network and an uncertainty-aware regression head, integrating fine-grained local details and global contextual information to improve the accuracy and robustness of pose estimation. These methods significantly improve the continuity and accuracy of pose estimation through the modeling of sequence information. (2) The second is the human pose prior introduction direction: such methods [10,11] constrain the generation space of 3D poses by incorporating prior knowledge such as human anatomical structures and pose distributions, alleviating the estimation uncertainty caused by depth ambiguity. Among them, diffusion model-based methods [12,13] have achieved the current State-of-the-Art (SOTA) performance in this field by directly regressing 3D joint coordinates. For example, Shi et al. [14] proposed the motion-semantics-based diffusion (MS-Diff) framework for monocular video-based 3D human pose estimation, which integrates high-level motion semantics via a Multimodal Diffusion Interaction (MDI) module and applies spectral feature regularization through a Spectral Convolutional Regularization (SCR) module to reduce noise, resolve pose ambiguities, and handle occlusions effectively. And disentangled-based methods [15,16] have taken a different approach by decomposing the 3D pose estimation task into subtasks of bone length and bone direction prediction, and explicitly incorporating human pose prior constraints based on the forward kinematic structure of the human skeleton. For example, Liu et al. [17] proposed a novel framework that achieves viewpoint invariance for 3D human pose estimation by explicitly disentangling motion and view features through a lightweight view estimator and a view-aware inference pipeline.

In recent years, monocular 3D HPE technology has witnessed rapid development, and researchers have proposed a variety of solutions to address the core challenge of depth ambiguity, which can be mainly divided into two research veins. (1) The first is the spatiotemporal information mining direction: such methods leverage Convolutional Neural Networks (CNNs) [7] or Transformer architectures [8] to fully explore temporal information and spatial context information in video sequences, thereby compensating for the depth information loss in the 2D-to-3D mapping. For example, Bian et al. [9] proposed a novel human pose estimation framework that adopts a task-specific backbone network and an uncertainty-aware regression head, integrating fine-grained local details and global contextual information to improve the accuracy and robustness of pose estimation. These methods significantly improve the continuity and accuracy of pose estimation through the modeling of sequence information, and can better adapt to the analysis requirements of continuous high-dynamic movements in sports scenarios. (2) The second is the human pose prior introduction direction: such methods [10,11] constrain the generation space of 3D poses by incorporating prior knowledge such as human anatomical structures and pose distributions, alleviating the estimation uncertainty caused by depth ambiguity. Among them, diffusion model-based methods [12,13] have achieved the current State-of-the-Art (SOTA) performance in this field by directly regressing 3D joint coordinates. For example, Shi et al. [14] proposed the motion-semantics-based diffusion (MS-Diff) framework for monocular video-based 3D human pose estimation, which integrates high-level motion semantics via a Multimodal Diffusion Interaction (MDI) module and applies spectral feature regularization through a Spectral Convolutional Regularization (SCR) module to reduce noise, resolve pose ambiguities, and handle occlusions effectively. And disentangled-based methods [15,16] have taken a different approach by decomposing the 3D pose estimation task into subtasks of bone length and bone direction prediction, and explicitly incorporating human pose prior constraints based on the forward kinematic structure of the human skeleton. This idea is highly consistent with the kinematic analysis logic in sports biomechanics, and has natural application potential in the field of sports technology analysis. For example, Liu et al. [17] proposed a novel framework that achieves viewpoint invariance for 3D human pose estimation by explicitly disentangling motion and view features through a lightweight view estimator and a view-aware inference pipeline.

**Challenges**. Despite the remarkable progress made by existing methods, both diffusion model-based methods and disentangled-based methods still have their own limitations, and neither of the two types of methods has fully explored the hierarchical structural information of the human skeleton, resulting in an insurmountable bottleneck in model performance. Specifically, there are three core challenges: (1) Existing diffusion model-based methods [12–14] directly add Gaussian noise to the original 3D pose as a whole without performing structural decomposition on the 3D pose, making it impossible to effectively learn explicit prior knowledge such as the temporal consistency of bone length and the variation rules of joint angles in the human skeleton. The learning process of the model lacks the constraints of human body structure, resulting in an insufficiently refined modeling of 3D poses; (2) Although disentangled-based methods [15–17] can explicitly incorporate the anatomical priors of the human skeleton through the decomposition of bone length and bone direction, affected by the tree-like hierarchical structure of the human skeleton, the prediction errors of bone length and bone direction will continuously propagate and amplify along the skeletal hierarchy, forming a severe hierarchical error accumulation effect. This ultimately leads to their overall performance being far lower than that of mainstream diffusion model-based methods, failing to exert the practical effect of human pose priors. In sports scenarios, this error accumulation will be more prominent in terminal joints such as hands and ankles that are critical for technical movement evaluation, directly affecting the reliability of sports motion analysis; (3) Whether it is Transformer-based spatiotemporal modeling methods [8,9], or diffusion model-based and disentangled-based 3D pose estimation methods [18–20], all ignore the fine-grained hierarchical correlations of the human skeleton. The human skeleton can be divided into multiple hierarchies according to the depth of the kinematic tree, and the spatial position and temporal variation of each joint are strongly correlated with its parent and child joints. With the increase of joint hierarchy, the estimation error of existing methods rises significantly, and hierarchical error accumulation has become a key factor restricting model performance.

To address the above core issues, we propose a Hierarchy-Aware Disentangled Diffusion Framework with Spatio-Temporal Denoising (HADD), which innovatively combines the disentanglement strategy with the diffusion model and deeply integrates the hierarchical information of the human skeleton into the forward and reverse processes of the diffusion model. HADD realizes the dual optimization of structured prior learning and hierarchical spatiotemporal modeling.

The core design ideas of HADD are as follows: (1) on the one hand, the 3D pose is disentangled into bone length and bone direction in the forward process of the diffusion model, and we add noise to the disentangled bone length and bone direction respectively, allowing the model to focus on the temporal consistency of bone length and the spatial variation rules of bone direction separately. HADD can learn the explicit priors of human pose more efficiently, while avoiding the hierarchical error accumulation caused by direct disentangled prediction; (2) on the other hand, in the reverse denoising process of the diffusion model, we design a targeted hierarchical spatiotemporal denoiser to strengthen the spatial and temporal correlations between each joint and its parent and child joints, accurately modeling the hierarchical structural information of the human skeleton and alleviating the estimation error of higher-hierarchy joints.

The contributions of our work can be summarized as the following three points:

1. We deeply integrate disentangled representation strategy with the diffusion model, and fully embed the hierarchical structural information of the human skeleton into the full pipeline of the diffusion model. This design alleviates two core limitations of existing methods, namely the lack of explicit human anatomical prior constraints in diffusion-based methods and severe hierarchical error accumulation in disentangled-based methods.
2. Based on the hierarchical kinematic structure of the human skeleton, we disentangle the 3D pose into two independent feature dimensions: bone length and bone direction. And we add Gaussian noise to each of them in the diffusion forward process. Meanwhile, we design a disentanglement loss function to guide model training, which enables the model to efficiently learn the explicit prior knowledge of human pose.
3. For the reverse denoising process, we design a Hierarchical Spatial and Temporal Denoising (HSTD) module, which consists of HSDM and HTDM sub-modules to accurately model the hierarchical spatio-temporal relationships of the human skeleton and mitigate error amplification of high-hierarchy joints. Meanwhile, we design a hybrid loss function to realize dual supervision at the bone level and joint level, further improving the accuracy and structural rationality of pose estimation.

## 2. Related work

We focus on diffusion-based 3D human pose estimation integrated with the core ideas of disentangled representation and hierarchical spatiotemporal modeling. Relevant research is mainly divided into two directions: 3D human pose estimation and diffusion models and their applications in pose estimation. Existing studies still have room for optimization in terms of human prior fusion, hierarchical information modeling, and diffusion model adaptation. The research progress and limitations of the two directions are reviewed.

### 2.1. 3D human pose estimation

3D Human Pose Estimation (3D HPE) is one of the core tasks in the field of computer vision, which strongly supports downstream applications such as virtual reality, human action recognition, and human-computer interaction. Its core goal is to regress the 3D spatial coordinates of human joints from monocular 2D pose sequences. Current mainstream methods can be categorized into one-stage methods and two-stage methods: One-stage methods directly extract features from raw RGB images and regress 3D poses. Typical works [21,22] use convolutional neural networks to model image feature volumes for end-to-end pose prediction, yet the estimation accuracy in the depth dimension is difficult to guarantee due to factors such as image background and human occlusion. Two-stage methods [23,24] first detect 2D keypoints in images using mature 2D pose estimation algorithms, and then complete 3D pose inference through 2D-to-3D lifting. This method has more advantages in accuracy and robustness as it fully leverages the mature technology of 2D pose estimation, and thus has become the current mainstream research paradigm.

To alleviate the depth ambiguity problem in the 2D-to-3D mapping process, existing two-stage methods are mainly optimized from two perspectives: spatiotemporal information fusion and introduction of human pose priors. In terms of spatiotemporal information fusion, early studies used convolutional networks to mine temporal information to make up for the information loss in the depth dimension. Subsequent Transformer-based methods further fuse spatial and temporal context information and improve the accuracy of 3D pose estimation by capturing the spatiotemporal correlation of keypoints, with representative works including MixSTE [19], PoseFormer [25], and PoseFormerV2 [26]. For example, Zhang et al. [19] proposed MixSTE (Mixed Spatio-Temporal Encoder), which consists of alternating temporal and spatial transformer blocks to separately model each joint’s temporal motion and inter-joint spatial correlation, and extends the output to the entire input video sequence for better coherence. Zheng et al. [25] proposed a purely transformer-based model for 3D human pose estimation in videos (PoseFormer), which designs a spatial-temporal transformer structure to model intra-frame human joint relations and inter-frame temporal correlations without using any convolutional architectures. Although such methods exhibit strong capabilities in temporal modeling, they generally pay insufficient attention to the fine-grained hierarchical information of the human skeleton. The human skeleton presents a tree-like kinematic structure, and keypoints can be divided into different hierarchies according to kinematic depth. Existing methods ignore such hierarchical correlation, leading to the continuous accumulation of errors with the increase of hierarchies and ultimately affecting the overall estimation performance.

In terms of introducing human pose priors, a class of disentangled methods has become an important research branch. These methods decompose 3D pose estimation into independent prediction tasks of bone length and bone direction, and then reconstruct 3D joint coordinates based on the forward kinematics of the human skeleton, which can explicitly incorporate human anatomical priors such as temporal consistency of bone length, joint angle limits, and human symmetry loss constraints, with representative works including Anatomy3D [18], DKA [27], and Virtual Bones [28]. For example, Chen et al. [18] proposed a novel 3D human pose estimation model that decomposes the task into bone direction prediction and bone length prediction inspired by human skeleton anatomy. Xu et al. [27] proposed a deep kinematics analysis (DKA) pipeline that systematically integrates 2D kinematics structure optimization, human-topology-based articulated motion decomposition, and a temporal refinement module to model both static and dynamic structures for monocular 3D pose estimation. Although disentangled methods can effectively fuse human priors, they have a key problem of hierarchical error amplification: since the 3D joint coordinates are derived layer by layer based on the bone parameters of parent and child joints, the prediction errors of bone length and direction will accumulate continuously in hierarchical propagation, ultimately resulting in their performance being significantly lower than that of current mainstream non-disentangled methods, which also becomes a core bottleneck for the practical application of disentangled methods.

### 2.2. Diffusion models

As a class of generative models, diffusion models have achieved breakthrough results in image generation, super-resolution, semantic segmentation, multi-modal tasks and other fields by virtue of their strong probabilistic modeling and denoising capabilities. The core framework of the model includes a forward diffusion process and a reverse denoising process: the forward process gradually adds Gaussian noise to the original data until the data eventually degenerates into a random noise distribution; the reverse process recovers the original data from the noisy data by training a denoising model. Classic works such as DDPM (Denoising diffusion probabilistic models) [29] and DDIM (Denoising Diffusion Implicit Models) [30] have simplified and accelerated the training and sampling process of diffusion models, laying a foundation for their applications in various fields.

In recent years, researchers have begun to explore the application of diffusion models in the 3D human pose estimation task, aiming to solve the uncertainty problem in 3D pose estimation by using their probabilistic modeling capabilities. Most existing diffusion-based 3D HPE methods [31,32] take the original 3D pose as the diffusion object, directly add noise to the 3D joint coordinates in the forward process, and then recover the real pose from the noisy 3D pose through the reverse denoising process. For example, Liu et al. [31] proposed a novel Uncertainty-Guided Diffusion Model for 3D Human Pose Estimation (UDM-HPE) that uses uncertainty-aware probabilistic guidance to improve the denoising process. Some works also combine strategies such as multi-hypothesis aggregation, gradient field prior, and heatmap distribution initialization to improve performance, with representative works including D3DP [20], GFPose [33], and DiffPose [34]. For example, Shan et al. [20] proposed a novel probabilistic 3D human pose estimation method named D3DP (Diffusion-based 3D Pose estimation) combined with JPMA (Joint-wise re-Projection-based Multi-hypothesis Aggregation), where D3DP generates multiple 3D pose hypotheses via a diffusion model conditioned on 2D keypoints, and JPMA aggregates these hypotheses joint-by-joint using reprojection errors to produce the final pose. Although such methods can use the advantages of diffusion models to improve the robustness of pose estimation, they have a key problem of insufficient fusion of explicit human priors: directly diffusing 3D joint coordinates without combining the disentangled representation of the human skeleton makes it difficult to explicitly incorporate human anatomical priors such as bone length and direction, resulting in the model being unable to fully utilize the inherent constraints of the human structure and limiting the further improvement of estimation accuracy.

At the same time, existing diffusion-based 3D HPE methods still inherit the problems of traditional Transformer methods, i.e., insufficient exploration of hierarchical spatiotemporal information of the human skeleton. The design of the denoising model does not consider the hierarchical correlation of keypoints and cannot effectively alleviate the problem of hierarchical error accumulation. In addition, the error accumulation problem of disentangled methods and the insufficient prior fusion problem of diffusion models are independent of each other.

## 3. Methodology

This section elaborates on the technical details of the proposed HADD, which integrates disentanglement strategies into diffusion models and leverages hierarchical spatio-temporal modeling to mitigate error accumulation. The overall pipeline of HADD is illustrated in Fig 1, which consists of four core components: a 3D pose disentanglement strategy that decomposes joint coordinates into bone length and direction, a forward diffusion process with dimension-wise noise injection, a reverse diffusion process empowered by a Hierarchical Spatial and Temporal Denoising Module (HSTD), and a loss function that unifies disentanglement supervision and pose regression constraints. All mathematical formulations are rigorously derived and explained, with notations and definitions standardized for consistency with the state-of-the-art literature in diffusion models and 3D HPE.

**Fig 1:**
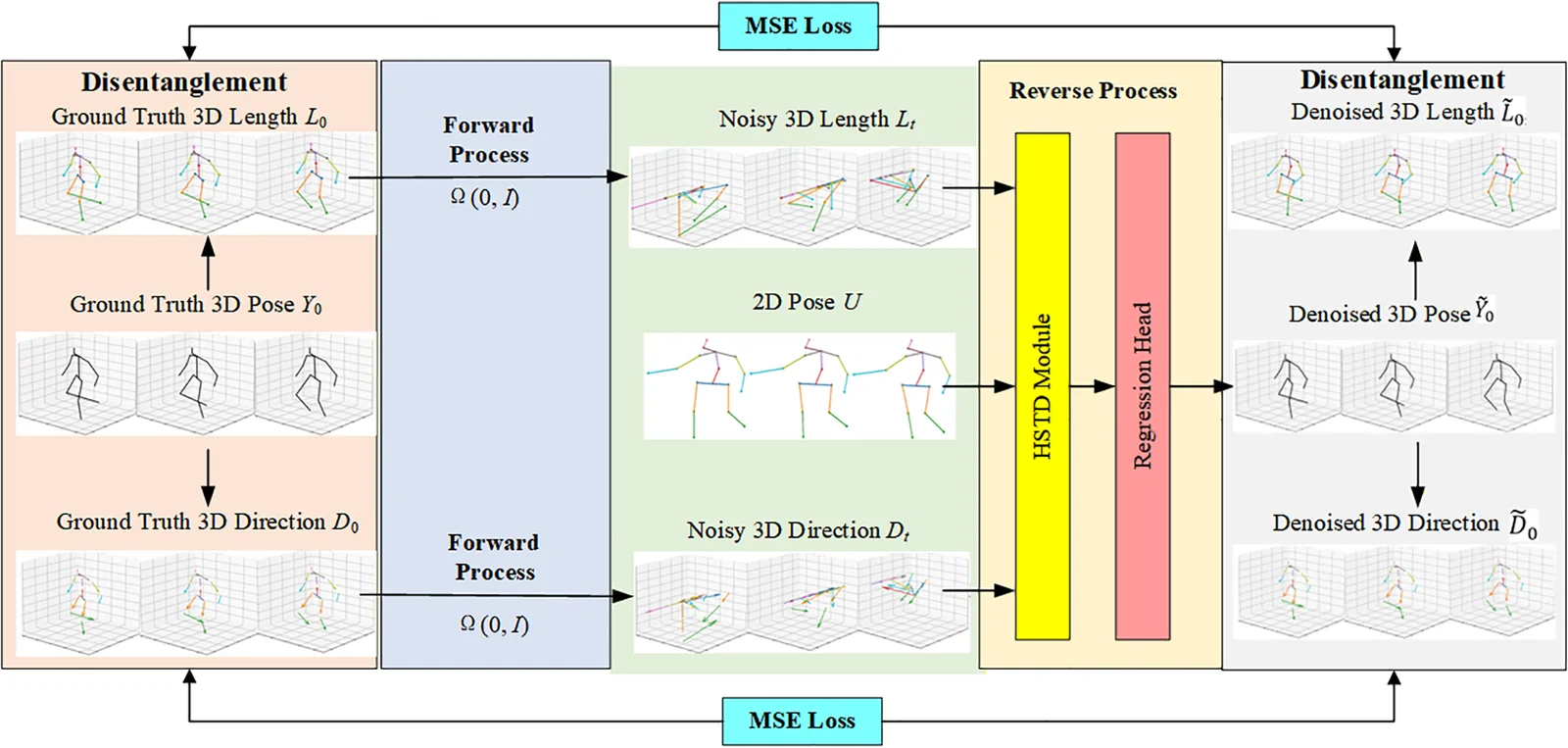
The overview of HADD’s training pipeline.

**Ethics statements**. This study only uses publicly available and strictly de-identified academic benchmark datasets (Human3.6M and MPI-INF-3DHP), and no new human subject data collection was conducted. Both datasets have passed the ethical review of their original publishing institutions, all subjects have signed informed consent forms, and the data do not contain any personally identifiable information. The use of the datasets in this study strictly follows the ethical requirements and usage agreements at the time of their release, and complies with the academic ethical norms in the field of computer vision. Therefore, no additional ethical review approval is required.

### 3.1. Preliminaries

**Definition 1 (Joint hierarchy division rule).** *We select 17 human core joint points as the pose representation units, based on the following rationale* [10]*: this joint set constitutes the minimal necessary set of core joints in line with human anatomy and kinematics, which can fully cover the key nodes of the motion chain of the trunk, limbs and head. In addition, the 17 joint points can be naturally decomposed into 16 bone segments, which is well adapted to the feature decomposition of bone length and bone direction in the disentangled diffusion strategy. We divide the 17 human joints into 6 hierarchies according to the depth from the root joint of the kinematic tree, with the following specific division, as shown in* Table 1:

**Table 1:** Joint hierarchy division rule.

Hierarchy	Layer Name	Joint Name	Descriptions
0	Root Node Layer	Pelvis root	The starting node of the entire skeletal kinematic tree
1	Trunk Proximal Layer	Left hip, right hip, spine (spine 1)	Direct child joints of Hierarchy 0
2	Trunk & Limb Mid Layer	Left knee, right knee, chest (spine 2)	Direct child joints of Hierarchy 1 joints
3	Limb Proximal & Neck Layer	Left ankle, right ankle, neck, left shoulder, right shoulder	Direct child joints of Hierarchy 2 joints
4	Limb Mid & Head Layer	Head, left elbow, right elbow	Direct child joints of Hierarchy 3 joints
5	Terminal Limb Layer	Left hand, right hand	Direct child joints of Hierarchy 4 joints

The division strictly abides by the inherent tree-like kinematic structure of the human skeleton, taking the pelvic root node as the root and classifying the joints step by step from the proximal trunk to the terminal limbs according to their depth in the kinematic tree, which accurately matches the parent-child dependency among human joints.

**Definition 2 (Pinhole Camera Projection Model).** *We adopt the standard pinhole camera model for 3D-to-2D projection, which is fully compatible with the camera calibration parameters provided by the Human3.6M and MPI-INF-3DHP datasets. For the 3D joint coordinate* $Y_{n,j}\in {\mathbb{R}}^{3}$ *(*n∈[1,N] *is the frame index,* j∈[1,J] *is the joint index), its 2D projection coordinate* $Y_{n,j}^{\prime }\in {\mathbb{R}}^{2}$ *on the image plane is jointly determined by the camera intrinsic and extrinsic parameters. The projection formula is as follows:*


$$\begin{array}{c} Y_{n,j}^{\prime }=\pi \left(K\cdot \left(R\cdot Y_{n,j}+g\right)\right) \end{array}$$ (1)

*where* $K\in {\mathbb{R}}^{3\times 3}$ *is the camera intrinsic matrix provided by the dataset (containing focal length and principal point coordinates),* $R\in {\mathbb{R}}^{3\times 3}$ *and* $g\in {\mathbb{R}}^{3\times 1}$ *are the camera extrinsic parameters (rotation and translation matrices).* π(·) *is a conversion function from homogeneous coordinates to inhomogeneous coordinates, defined as:*


$$\begin{array}{c} \pi \left([\begin{array}{c} u\\ v\\ w \end{array}]\right)=[\begin{array}{c} u/w\\ v/w \end{array}],\;w\text{≠}0. \end{array}$$

For a complete 3D pose sequence $Y\in {\mathbb{R}}^{N\times J\times 3}$ containing *N* frames and *J* joints, the projected 2D pose sequence is $Y^{\prime }\in {\mathbb{R}}^{N\times J\times 2}$, which is completely consistent in dimension with the input 2D pose sequence $U\in {\mathbb{R}}^{N\times J\times 2}$, providing a unified dimensional basis for subsequent reprojection error calculation.

We have established a unified key symbol definition system for the full text (in Table 2). It should be mentioned that the explanations of temporary variables are provided at their first appearance in the text.

**Table 2:** Unified key symbol and variable definition.

Symbol	Dimension	Definition
*N*	Scalar	Number of frames in the input video sequence
*J*	Scalar	Total number of human skeletal joints
*B*	Scalar	*J*-1, the number of bone segments in the human skeletal kinematic tree (excluding the root joint with no parent joint)
*T*	Scalar	Total diffusion steps
*t*	Scalar	Current diffusion timestep
*U*	${\mathbb{R}}^{N\times J\times 2}$	Input conditional 2D pose sequence
*Y*_0_	${\mathbb{R}}^{N\times J\times 3}$	Ground-truth clean 3D joint coordinate sequence
${\hat{Y}}_{0}$	${\mathbb{R}}^{N\times J\times 3}$	Model-predicted clean 3D joint coordinate sequence
*L*_0_	${\mathbb{R}}^{N\times B\times 1}$	Ground-truth clean bone length sequence
*D*_0_	${\mathbb{R}}^{N\times B\times 3}$	Ground-truth clean bone direction sequence
$L_{t}$	${\mathbb{R}}^{N\times B\times 1}$	Noisy bone length sequence at timestep *t*
$D_{t}$	${\mathbb{R}}^{N\times B\times 3}$	Noisy bone direction sequence at timestep *t*
${\hat{L}}_{0}$	${\mathbb{R}}^{N\times B\times 1}$	Predicted bone length sequence
${\hat{D}}_{0}$	${\mathbb{R}}^{N\times B\times 3}$	Predicted bone direction sequence
$\beta_{t}$	Scalar	Noise schedule coefficient at timestep *t*
$\alpha_{t}$	Scalar	$\alpha_{t}=1-\beta_{t}$
*O*	Scalar	N·J (the total number of joint-frame elements in the sequence)
$\sigma_{t}$	Scalar	Standard deviation of the additional Gaussian noise in DDIM reverse sampling; $\sigma_{t}^{2}$ denotes its corresponding variance
*H*	Scalar	Number of multi-hypothesis samples
*Z*	Scalar	Number of DDIM iterative sampling rounds
$\epsilon_{l},\epsilon_{d}$	${\mathbb{R}}^{N\times B\times 1}$, ${\mathbb{R}}^{N\times B\times 3}$	Standard Gaussian noise samples for bone length and direction, respectively
$\epsilon_{tl}$, $\epsilon_{td}$	${\mathbb{R}}^{N\times B\times 1}$, ${\mathbb{R}}^{N\times B\times 3}$	Noise estimates derived for DDIM sampling

### 3.2. 3D pose disentanglement strategy

The fundamental motivation of the disentanglement strategy is to decouple the high-dimensional and densely correlated 3D joint coordinate optimization problem into low-dimensional, physically interpretable subproblems of bone length and bone direction prediction. This decomposition not only simplifies gradient-based optimization but also naturally embeds human anatomical priors (e.g., temporal consistency of bone length, kinematic constraints of bone direction) into the diffusion model, addressing the limitation of vanilla diffusion-based 3D HPE methods that fail to learn explicit human pose priors due to direct noise injection on 3D joint coordinates.

For a human skeletal structure with *J* joints, the 3D pose of a single frame is represented as $y_{0}\in {\mathbb{R}}^{J\times 3}$, where each element denotes the 3D coordinate of a joint in the Cartesian space. For a video sequence with *N* frames, the ground-truth 3D pose sequence is defined as *Y*_0_. Each bone is uniquely associated with a parent joint $p_{i}$ and a child joint $c_{i}$. For the *i*-th bone, its ground-truth length $L_{0}^{i}$ and unit direction vector $D_{0}^{i}$ are mathematically formulated as:


$$\begin{array}{c} \left\{ \begin{array}{c} L_{0}^{i}={\left\|Y_{0}^{c_{i}}-Y_{0}^{p_{i}}\right\|}_{2}\\ D_{0}^{i}=\frac{Y_{0}^{c_{i}}-Y_{0}^{p_{i}}}{{\left\|Y_{0}^{c_{i}}-Y_{0}^{p_{i}}\right\|}_{2}} \end{array}\right. \end{array}$$ (2)

where ${\left\|\cdot \right\|}_{2}$ denotes the Euclidean L2 norm, which quantifies the physical length of the bone between the parent and child joint. The $Y_{0}^{p_{i}}$, $Y_{0}^{c_{i}}\in {\mathbb{R}}^{N\times J\times 3}$ represent the 3D coordinates of the parent and child joints of the *i*-th bone in the ground-truth pose. The unit direction vector is normalized to have a magnitude of 1, thus isolating the directional information of the bone from its length. For the entire video sequence, the disentangled ground-truth bone length sequence and bone direction sequence are denoted as *L*_0_ and *D*_0_, respectively.

The disentanglement of 3D poses into *L*_0_ and *D*_0_ confers two critical properties for diffusion-based 3D HPE. Properties of the disentangled representation are as follows: (1) The original optimization space of N×J×3 is split into two independent spaces of N×B×1 (bone length) and N×B×3 (bone direction), reducing the number of trainable parameters and alleviating the problem of vanishing gradients in high-dimensional space; (2) Bone length is a time-invariant scalar for a given human subject (i.e., $L_{0}^{i}$ remains constant across video frames), while bone direction is constrained by physiological joint angle limits. These priors can be explicitly enforced via loss functions, ensuring the physical plausibility of the predicted 3D pose.

From a theoretical perspective, the rationality of confining disentanglement to the forward diffusion process is rooted in the inherent separability and heterogeneity of human skeletal motion features: (1) Human skeletal motion follows a tree-like hierarchical kinematic chain, where bone length (a time-invariant scalar) and bone direction (a spatiotemporally dynamic 3D vector) exhibit distinct statistical distributions. Bone length remains nearly constant across frames for a specific individual, while bone direction is constrained by physiological joint angle limits and exhibits motion-pattern-dependent variation. This inherent separability ensures that disentangled features can be independently modeled without information loss; (2) According to the principle of disentangled representation in information theory, decomposing the joint coordinate space into bone length and bone direction spaces minimizes the mutual information between features, reducing the model’s learning complexity from optimizing a high-dimensional joint distribution *p*(*Y*|*U*) to two low-dimensional marginal distributions *p*(*L*|*U*) and *p*(*D*|*U*) (*U* denotes input 2D pose sequences). This dimensionality reduction alleviates vanishing gradients in high-dimensional optimization and accelerates convergence.

### 3.3. Forward diffusion process

The forward diffusion process of HADD follows the Markov chain formulation of Denoising Diffusion Probabilistic Models (DDPM) [29], and we apply the dimension-wise Gaussian noise injection to the disentangled bone length *L*_0_ and bone direction *D*_0_ instead of the original 3D joint coordinates. This operation enables the diffusion model to learn the temporal dynamics of bone length and direction separately, preserving human anatomical priors during the noise addition process.

For a generic data sample *x*_0_, the forward diffusion process gradually adds independent Gaussian noise to *x*_0_ over *T* steps, generating a noisy sample $x_{t}$ at step *t* (1≤t≤T). The process is defined as a Markov chain with a fixed noise schedule (the formula format consis*t*ent with the DDPM), where the conditional probability distribution of $x_{t}$ given *x*_0_ is:


$$\begin{array}{c} q(x_{t}|x_{t-1})=𝒩\left(x_{t};\sqrt{1-\beta_{t}}\;x_{t-1},\;\beta_{t}I\right) \end{array}$$ (3)

$$\begin{array}{c} q(x_{t}|x_{0})=𝒩\left(x_{t};\sqrt{{\bar{\alpha }}_{t}}\;x_{0},\;(1-{\bar{\alpha }}_{t})I\right) \end{array}$$ (4)

where 𝒩(·;μ,Σ) denotes the multivariate Gaussian distribution with mean μ and covariance matrix Σ; ${\bar{\alpha }}_{t}$ is the cumulative product of $\alpha_{s}$ from step 0 to step *t*; $\beta_{s}$ is the noise schedule coefficient^35^ that satisfies $0<\beta_{1}<\beta_{2}<\cdots <\beta_{T}<1$; I is the identity matrix of appropriate dimension, ensuring noise is independently added to each dimension of the data.

At step *t*, the noisy sample $x_{t}$ can be directly derived from *x*_0_ (bypassing intermediate steps) using the reparameterization trick:


$$\begin{array}{c} x_{t}=\sqrt{{\bar{\alpha }}_{t}}\;x_{0}+\sqrt{1-{\bar{\alpha }}_{t}}\;\epsilon \end{array}$$ (5)

where ϵ is a standard Gaussian noise sample independent of *x*_0_.

Applying Equation 5 to the disentangled bone length sequence *L*_0_ and bone direction sequence *D*_0_, the noisy bone length $L_{t}\in {\mathbb{R}}^{N\times B\times 1}$ and noisy bone direction $D_{t}\in {\mathbb{R}}^{N\times B\times 3}$ at step *t* are obtained as:


$$\begin{array}{c} \left\{ \begin{array}{c} L_{t}=\sqrt{{\bar{\alpha }}_{t}}\;L_{0}+\sqrt{1-{\bar{\alpha }}_{t}}\;\epsilon_{l}\\ D_{t}=\sqrt{{\bar{\alpha }}_{t}}\;D_{0}+\sqrt{1-{\bar{\alpha }}_{t}}\;\epsilon_{d} \end{array}\right. \end{array}$$ (6)

where $\epsilon_{l}\sim 𝒩(0,I)$ and $\epsilon_{d}\sim 𝒩(0,I)$ are independent standard Gaussian noise samples for bone length and bone direction, respectively. The use of separate noise samples ensures that the noise dynamics of bone length (a scalar) and bone direction (a 3D vector) are modeled independently, avoiding cross-dimensional interference that would otherwise obscure anatomical priors. As t→T, ${\bar{\alpha }}_{t}\to 0$ and $(1-{\bar{\alpha }}_{t})\to 1$, this means $L_{t}$ and $D_{t}$ converge to pure Gaussian noise. This property is consistent with DDPM and ensures the reverse diffusion process can be trained to recover the original disentangled features from random noise.

### 3.4. Reverse diffusion process

The reverse diffusion process is the inverse of the forward process, aiming to recover the original disentangled bone length *L*_0_ and bone direction *D*_0_ from the noisy samples $L_{t}$ and $D_{t}$ (and ultimately from pure Gaussian noise at inference time). Unlike the forward process, which has a fixed closed-form distribution, the reverse process is a learned conditional distribution $p_{\theta }$, where θ denotes the trainable parameters of the model and *U* is the input 2D pose sequence (the conditional context for 3D HPE). The HSTD (Hierarchical Spatial and Temporal Denoising) module is the core of $p_{\theta }$, which models the hierarchical spatio-temporal correlations of human joints to denoise $L_{t}$ and $D_{t}$ effectively. Our Hierarchical Spatial and Temporal Denoising (HSTD) module, combined with the regression head, explicitly predicts the clean 3D joint coordinate sequence ${\hat{Y}}_{0}$ as its only direct output. It does not predict the Gaussian noise term as in vanilla DDPM, nor does it directly predict the clean disentangled bone length ${\hat{L}}_{0}$ and bone direction ${\hat{D}}_{0}$.

In the training stage, the reverse process takes the concatenated noisy features $[L_{t},D_{t}]\in {\mathbb{R}}^{N\times B\times 4}$ and the input 2D pose sequence *U* as inputs, and outputs the denoised 3D joint sequence ${\hat{Y}}_{0}$ via the HSTD module and a regression head. ${\hat{Y}}_{0}$ is the final direct output of the denoising network, while ${\hat{L}}_{0}$ and ${\hat{D}}_{0}$ are derived by secondary decomposition of ${\hat{Y}}_{0}$ using Equation 2. The conditional distribution of the reverse denoising process is formulated as:


$$\begin{array}{c} p_{\theta }\left({\hat{Y}}_{0}|L_{t},D_{t},U,t\right)=𝒩\left({\hat{Y}}_{0};P_{\theta }\left(L_{t},D_{t},U,t\right),\sigma_{t}^{2}I\right) \end{array}$$ (7)

where $P_{\theta }(\cdot )$ denotes the HSTD network with trainable parameters θ, and $\sigma_{t}$ is the standard deviation of the additional Gaussian noise introduced in the DDIM reverse sampling procedure, with $\sigma_{t}^{2}$ being its corresponding variance. This parameter controls the stochasticity of the sampling process and the variance of the predicted 3D pose distribution. *t* denotes the current diffusion timestep.

**Remark.** Camera intrinsic and extrinsic parameters (*K*, *R*, *g*) are *not* conditions of the denoising network. They are only introduced in the multi-hypothesis selection stage at inference time to compute 2D reprojection errors for hypothesis ranking, and do not participate in the reverse diffusion denoising computation.

The ground-truth 3D pose sequence is denoted as *Y*_0_, and the last dimension corresponds to the 3D Cartesian coordinates (*x*, *y*, *z*) of each joint. The denoised 3D pose sequence predicted by the model is denoted as ${\hat{Y}}_{0}$. For both *Y*_0_ and ${\hat{Y}}_{0}$, we perform disentanglement based on the human skeletal kinematic tree to obtain the ground-truth bone length *L*_0_, ground-truth bone direction *D*_0_ and predicted bone length ${\hat{L}}_{0}$, predicted bone direction ${\hat{D}}_{0}$, respectively. The disentanglement operation for the *i*-th bone (connecting parent joint $p_{i}$ and child joint $c_{i}$) follows the Euclidean norm and unit vector normalization as defined:


$$\begin{array}{c} \left\{ \begin{array}{c} {\hat{L}}_{0}^{i}={\left\|{\hat{Y}}_{0}^{c_{i}}-{\hat{Y}}_{0}^{p_{i}}\right\|}_{2}\\ {\hat{D}}_{0}^{i}=\frac{{\hat{Y}}_{0}^{c_{i}}-{\hat{Y}}_{0}^{p_{i}}}{{\left\|{\hat{Y}}_{0}^{c_{i}}-{\hat{Y}}_{0}^{p_{i}}\right\|}_{2}} \end{array}\right. \end{array}$$ (8)

where ${\hat{Y}}_{0}^{p_{i}}$, ${\hat{Y}}_{0}^{c_{i}}\in {\mathbb{R}}^{3}$ are the corresponding predicted coordinates of the 3D coordinates of the parent and child joints of the *i*-th bone in the ground-truth pose. The disentangled bone length is a scalar (*L*_0_, ${\hat{L}}_{0}$) and the bone direction is a 3D unit vector (*D*_0_, ${\hat{D}}_{0}$).

In the inference stage of the reverse diffusion process, to address the inherent depth ambiguity problem in monocular 2D-to-3D human pose lifting and further improve the accuracy and robustness of 3D pose prediction, our method draws on the multi-hypothesis sampling strategy proposed in D3DP [20], and combines the fast-sampling advantage of DDIM [30] to optimize the entire inference pipeline. Different from the fixed one-to-one denoising mode in the training stage, the inference stage adopts a multi-hypothesis iterative sampling mechanism for the disentangled bone length and direction features, which fully explores the multi-solution space of 2D-to-3D lifting and effectively avoids the prediction deviation caused by single random noise sampling.

Specifically, we first sample *H* independent hypothesis samples of initial noisy bone length and initial noisy bone direction from the standard Gaussian distribution 𝒩(0,I) at the same time, where the number of hypotheses *H* is a tunable hyper-parameter that determines the coverage of the solution space. These *H* groups of initial noisy bone length and direction features are then input into the pre-trained HSTD for the first round of denoising processing. Through the hierarchical spatio-temporal feature modeling and denoising capability of the HSTD, we obtain the denoised bone length set ${\hat{L}}_{0}^{\left(h\right)}$ and denoised bone direction set ${\hat{D}}_{0}^{\left(h\right)}$ corresponding to the *H* hypotheses. Following the standard DDIM sampling paradigm [30], we derive the timestep transition from the current step *t* to the next sampling step $t^{*}$ ($t^{*}<t$) for disentangled bone features. For each hypothesis *h*, we first compute the noise estima*t*es from the current noisy features and the predicted clean bone features:


$$\begin{array}{c} \epsilon_{tl}^{\left(h\right)}=\frac{L_{t}^{\left(h\right)}-\sqrt{{\bar{\alpha }}_{t}}\cdot {\hat{L}}_{0}^{\left(h\right)}}{\sqrt{1-{\bar{\alpha }}_{t}}},\;\epsilon_{td}^{\left(h\right)}=\frac{D_{t}^{\left(h\right)}-\sqrt{{\bar{\alpha }}_{t}}\cdot {\hat{D}}_{0}^{\left(h\right)}}{\sqrt{1-{\bar{\alpha }}_{t}}} \end{array}$$ (9)

where ${\hat{L}}_{0}^{\left(h\right)}$ and ${\hat{D}}_{0}^{\left(h\right)}$ are the predicted clean bone length and bone direction decomposed from ${\hat{Y}}_{0}^{\left(h\right)}$ via Equation 2. The DDIM update rule for noisy bone features from timestep *t* to the next subsampled timestep $t^{*}$ is then given by:


$$\begin{array}{c} \left\{ \begin{array}{cc} L_{t^{*}}^{\left(h\right)} & =\sqrt{{\bar{\alpha }}_{t^{*}}}\cdot {\hat{L}}_{0}^{\left(h\right)}+\sqrt{1-{\bar{\alpha }}_{t^{*}}-\sigma_{t}^{2}}\cdot \epsilon_{tl}^{\left(h\right)}+\sigma_{t}\cdot \epsilon_{l}^{\left(h\right)}\\ D_{t^{*}}^{\left(h\right)} & =\sqrt{{\bar{\alpha }}_{t^{*}}}\cdot {\hat{D}}_{0}^{\left(h\right)}+\sqrt{1-{\bar{\alpha }}_{t^{*}}-\sigma_{t}^{2}}\cdot \epsilon_{td}^{\left(h\right)}+\sigma_{t}\cdot \epsilon_{d}^{\left(h\right)} \end{array}\right. \end{array}$$ (10)

where the stochastic noise standard deviation $\sigma_{t}$ is defined as:


$$\begin{array}{c} \sigma_{t}=\sqrt{\frac{1-{\bar{\alpha }}_{t^{*}}}{1-{\bar{\alpha }}_{t}}}\cdot \sqrt{1-\frac{{\bar{\alpha }}_{t}}{{\bar{\alpha }}_{t^{*}}}} \end{array}$$

and $\epsilon_{l}^{\left(h\right)}\sim 𝒩(0,I)$, $\epsilon_{d}^{\left(h\right)}\sim 𝒩(0,I)$ are independent fresh standard Gaussian noise samples for each hypothesis. For deterministic DDIM sampling, we set $\sigma_{t}=0$ and omit the stochastic noise term.

We adopt the pinhole camera model defined in Definition 2 for 3D-to-2D projection. The projected 2D coordinate $Y^{\prime }\in {\mathbb{R}}^{N\times J\times 2}$ is dimensionally consistent with the input 2D pose *U*. For *H* independently generated 3D pose hypotheses $\left\{Y_{1},Y_{2},...,Y_{H}\right\}$, we first compute the average 2D reprojection error of each hypothesis relative to the input 2D pose sequence *U*:


$$\begin{array}{c} E_{h}=\frac{1}{O}\sum_{n=1}^{N}\sum_{j=1}^{J}{\left\|Y_{n,j}^{\prime h}-U_{n,j}\right\|}_{2} \end{array}$$ (11)

where $Y_{n,j}^{\prime h}$ denotes the 2D projection coordinate of the *j*-th joint in the *n*-th frame of the *h*-th hypothesis, calculated by Equation 1. The hypothesis with the minimum reprojection error is selected as the final output:


$$\begin{array}{c} {\hat{Y}}_{\text{final}}=\text{arg}\min_{h\in \{1,2,...,H\}}E_{h} \end{array}$$ (12)

This selection process relies solely on the input 2D joint sequence and dataset-provided camera calibration parameters, without using any 3D ground truth information, and represents a practical inference pipeline that can be directly deployed.

In summary, the specific implementation in HADD is as follow:

1. Model Forward Pass (Step 1): Input the initial noisy features $L_{t}$, $D_{t}$ and the 2D pose sequence *U*. The HSTD outputs the predicted clean 3D joint coordinates ${\hat{Y}}_{0}$;
2. Secondary Disentanglement (Step 2): Decompose ${\hat{Y}}_{0}$ to obtain the predicted bone length ${\hat{L}}_{0}$ and bone direction ${\hat{D}}_{0}$, as shown in Equation 8;
3. Noise Estimate Derivation (Step 3): According to Equation 6, we substitute $L_{t}$, ${\hat{L}}_{0}$ and $D_{t}$, ${\hat{D}}_{0}$ into the general DDPM formula respectively to obtain the intermediate noise estimates $\epsilon_{tl}$ and $\epsilon_{td}$;
4. Next DDIM Sampling Step (Step 4): According to DDIM formula, we use the derived $\epsilon_{tl}$ and $\epsilon_{td}$ to generate the next-step noisy features (Equation 10).

This closed-loop design of “forward disentanglement for noise injection → reverse prediction of joint coordinates → secondary disentanglement for supervision” ensures the consistency of disentangled features throughout the entire diffusion pipeline.

### 3.5. Hierarchical spatial and temporal denoising module (HSTD)

The Hierarchical Spatial and Temporal Denoising module (HSTD) serves as the core component of the HADD framework for the reverse diffusion process, which is designed to explicitly model the hierarchical spatial-temporal correlations of human skeletal joints and mitigate the hierarchical error accumulation inherent in disentangled 3D human pose estimation. Unlike conventional denoisers that treat joint features as independent tokens or only capture global spatial-temporal context, HSTD leverages the tree-like kinematic structure of the human skeleton to enhance the attention weight of hierarchical adjacent joints (parent-child joints) in both spatial and temporal dimensions. It consists of two complementary sub-modules: the Hierarchical Spatial Denoising Module (HSDM) and the Hierarchical Temporal Denoising Module (HTDM), which are alternately stacked for *d* iterative loops to refine joint feature representations. The overall architecture of HSTD is illustrated in Fig 2, with the core computational logic of HSDM and HTDM formalized in this section.

**Fig 2:**
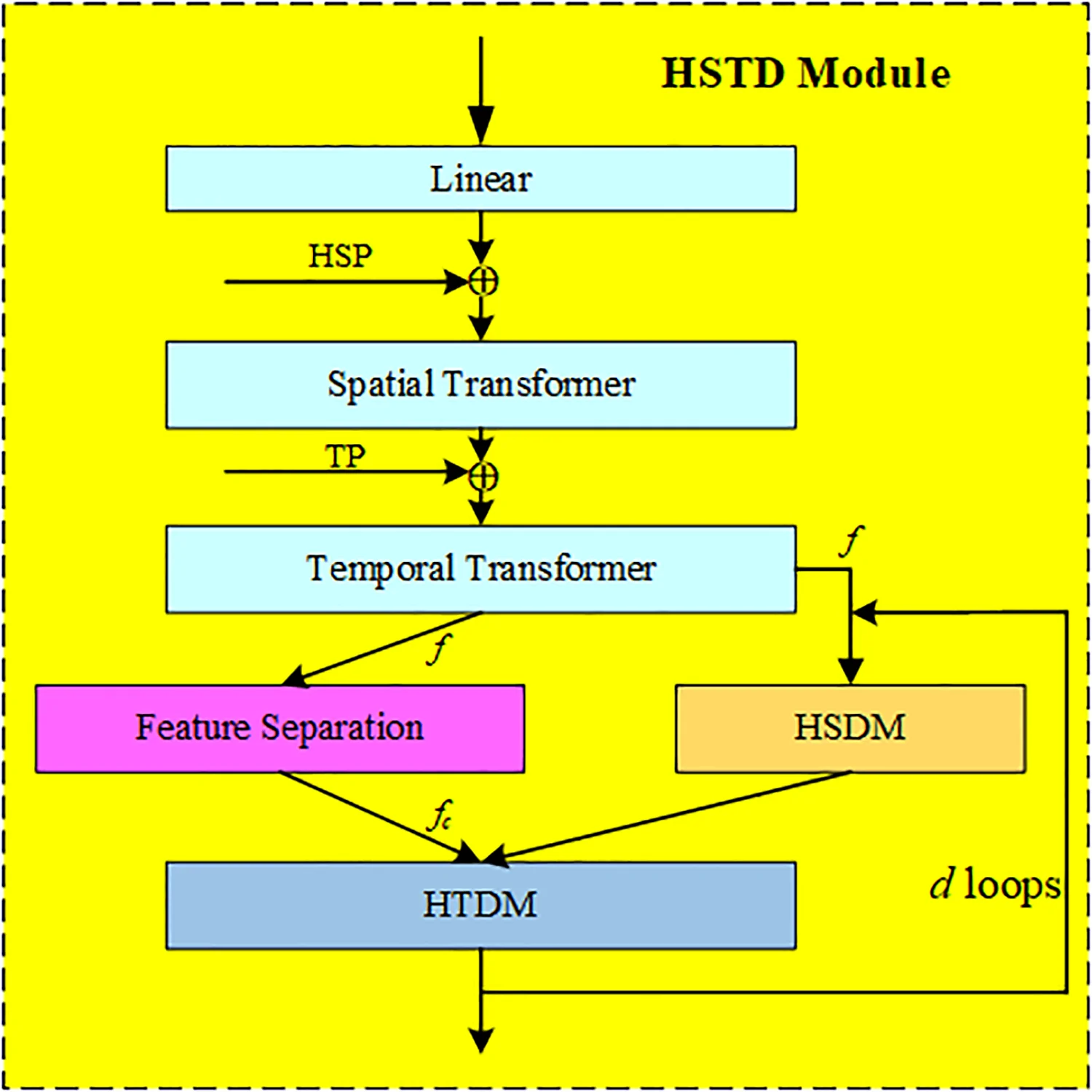
The architecture of HSTD module.

#### 3.5.1. Input feature embedding and initialization.

The input to HSTD is the concatenated feature tensor of noisy bone length $L_{t}$, noisy bone direction $L_{t}$ (generated from the forward diffusion process) and 2D pose sequence *U*, where *N* denotes the number of input video frames, *J* is the total number of human skeletal joints, and J−1 corresponds to the number of bone segments (parent-child joint pairs) in the kinematic tree. First, a linear projection layer Linear(·) is applied to map the concatenated raw features to a high-dimensional latent space with dimension *C*, yielding the initial joint feature tensor $F_{\text{init}}\in {\mathbb{R}}^{N\times J\times C}$:


$$\begin{array}{c} F_{\text{init}}=\text{Linear}(\text{Concat}(L_{t}^{*},D_{t}^{*},U)) \end{array}$$ (13)

where $L_{t}^{*}\in {\mathbb{R}}^{N\times J\times 1}$ are $D_{t}^{*}\in {\mathbb{R}}^{N\times J\times 3}$ the zero-padded versions of $L_{t}$ and $D_{t}$ to match the joint dimension *J*, and Concat(·) denotes the feature concatenation operation along the channel dimension. To explicitly encode the hierarchical and positional information of joints, two dedicated embedding modules are introduced, i.e., Hierarchical Spatial Position (HSP) Embedding $E_{\text{HSP}}\in {\mathbb{R}}^{J\times C}$ and Temporal Position (TP) Embedding $E_{\text{TP}}\in {\mathbb{R}}^{N\times C}$.

The prediction error of the parent joint will be continuously propagated and amplified along the kinematic tree to the terminal joints, making Hierarchy 5 the most severely affected by hierarchical error accumulation in existing methods. Joints in the same hierarchy share the same embedding vector, which is learned during training and encodes both spatial position and hierarchical affiliation of joints. We adopt a sinusoidal positional embedding to encode the temporal order of video frames, capturing the sequential nature of human pose sequences. The embeddings are added to the initial feature tensor to obtain the augmented feature tensor $F\in {\mathbb{R}}^{N\times J\times C}$ for subsequent transformer processing:


$$\begin{array}{c} F=F_{\text{init}}⊕E_{\text{HSP}}⊕E_{\text{TP}} \end{array}$$ (14)

where ⊕ denotes the broadcast addition operation to align the dimensions of spatial and temporal embeddings with *F*_init_. We apply a base spatio-temporal transformer block (Spatio-Temporal Encoder) [19] to *F* to capture global spatial-temporal context, generating the preprocessed joint feature tensor $f\in {\mathbb{R}}^{N\times J\times C}$, which serves as the input to HSDM and HTDM. We do not use flattened joint-frame space for mixed attention calculation. Spatial and temporal attention are executed alternately and strictly separated.

#### 3.5.2. Hierarchical spatial denoising module (HSDM).

HSDM is designed to model the hierarchical spatial dependency between parent and child joints, which is the fundamental spatial characteristic of the human skeletal kinematic tree. For any joint *j*, its spatial position is inherently determined by the position of its parent joint $j_{p}$ and the bone segment (length and direction) connecting them. Conventional spatial self-attention fails to emphasize this parent-child spatial correlation, leading to insufficient modeling of skeletal structural priors. HSDM addresses this by amplifying the attention weight of parent joints on child joints in the spatial attention matrix, formalizing the kinematic influence of parent joints on child joints in the attention mechanism. Fig 3 shows the architecture of HSDM.

**Fig 3:**
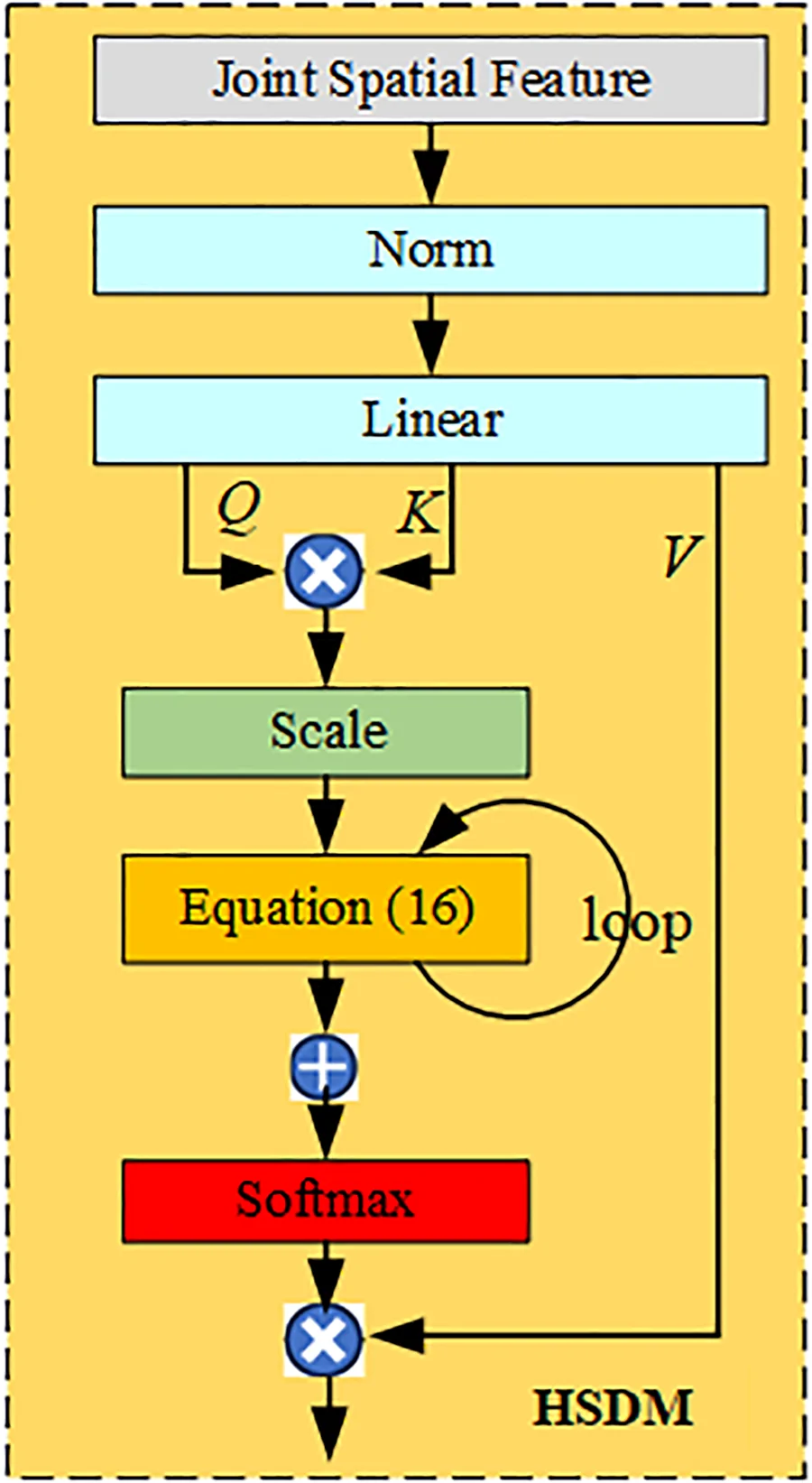
The architecture of HSDM.

For the preprocessed feature tensor *f*, the spatial dimension (joint dimension) is decoupled from the temporal dimension (frame dimension) by reshaping *f* to $f_{\text{spatial}}\in {\mathbb{R}}^{O\times C}$, where each row represents the feature of a single joint within one frame. We construct a frame-index-based binary mask. The attention scores between joints from different frames are set to −∞, so that HSDM only calculates valid spatial attention for joints within the same frame. The query *Q*, key *K*, and value *V* matrices for spatial self-attention are generated via linear projections with weight matrices $W_{Q},W_{K},W_{V}\in {\mathbb{R}}^{C\times C}$:


$$\begin{array}{c} Q=f_{\text{spatial}}W_{Q},\;K=f_{\text{spatial}}W_{K},\;V=f_{\text{spatial}}W_{V} \end{array}$$ (15)

The initial unnormalized spatial attention matrix $A\in {\mathbb{R}}^{O\times O}$ is computed as:


$$\begin{array}{c} A=\frac{QK^{⊤}}{\sqrt{d_{m}}} \end{array}$$ (16)

where $d_{m}=C/h$ is the dimension of each head in the multi-head attention (we set *h* = 8 for multi-head split), and $d_{m}$ is the scaling factor to mitigate the gradient vanishing problem caused by high-dimensional dot products.

Based on the predefined human skeletal kinematic tree, we define the set of hierarchical-related joint triplets as $\left\{J_{p},J,J_{c}\right\}$, where $J_{p}$ is the set of parent joints, *J* is the set of target joints, and $J_{c}$ is the set of child joints. For each triplet $\left(j_{p},j,j_{c})\in \{J_{p},J,J_{c}\right\}$, the attention weight from the parent joint $j_{p}$ to the target joint *j* ($A[j][j_{p}]$) is propagated to the attention weights between the target joint *j* and its child joint $j_{c}$ ($A[j][j_{c}]$) and between the child joint $j_{c}$ and the target joint *j* ($A[j_{c}][j]$). This propagation formalizes the spatial influence of the parent joint on the entire parent-child joint chain, and the refined attention matrix elements are computed as:


$$\begin{array}{c} A_{\text{HSDM}}[j][j_{c}]=\frac{A[j][j_{c}]+A[j][j_{p}]}{\lambda },\;A_{\text{HSDM}}[j_{c}][j]=\frac{A[j_{c}][j]+A[j_{p}][j]}{\lambda } \end{array}$$ (17)

The above attention propagation rule is derived from the inherent tree-like kinematic structure of the human skeleton. The spatial position of a child joint is physically determined by its parent joint and connected bone segments, so the attention correlation between target and child joints should inherit the parent-to-target attention weights. Direct superposition of attention weights will lead to gradient explosion and unstable training. Therefore, we adopt average normalization with a factor λ of 2 to constrain the amplitude of attention values. The selection of normalization factor is verified by ablation experiments, which proves that the factor λ of 2 achieves the best trade-off between constraint effect and training stability.

This refinement operation is applied to all hierarchical-related joint triplets in the skeletal tree), yielding the hierarchical spatial attention matrix *A*_HSDM_. The normalized hierarchical spatial attention matrix is obtained by applying the Softmax function to *A*_HSDM_, and the final hierarchical spatial feature tensor $f_{\text{HSDM}}\in {\mathbb{R}}^{O\times C}$ is computed as:


$$\begin{array}{c} f_{\text{HSDM}}=\text{Softmax}(A_{\text{HSDM}})V \end{array}$$ (18)

We reshape the tensor back to ${\mathbb{R}}^{N\times J\times C}$ to restore the temporal-frame and joint dimension, serving as the input to the subsequent HTDM module. We add a residual connection between the input *f* and the output *f*_HSDM_ of HSDM, followed by layer normalization LN(·) to stabilize training:


$$\begin{array}{c} f_{\text{out}}=\text{LN}(f+f_{\text{HSDM}}) \end{array}$$ (19)

#### 3.5.3. Hierarchical temporal denoising module (HTDM).

HTDM builds on the spatial hierarchical features from HSDM to model the hierarchical temporal correlation between target joints and their child joints. Human motion is a coherent hierarchical dynamic process: the movement of a parent joint drives the correlated movement of its child joints, leading to strong temporal dependencies between hierarchical adjacent joints. Conventional temporal self-attention only captures global temporal context across frames but neglects these fine-grained hierarchical temporal correlations. HTDM addresses this by introducing a residual cross-attention branch that explicitly models the temporal correlation between target joints and their child joints, fusing it with the global temporal self-attention to refine the temporal feature representation. Fig 4 shows the architecture of HTDM.

**Fig 4:**
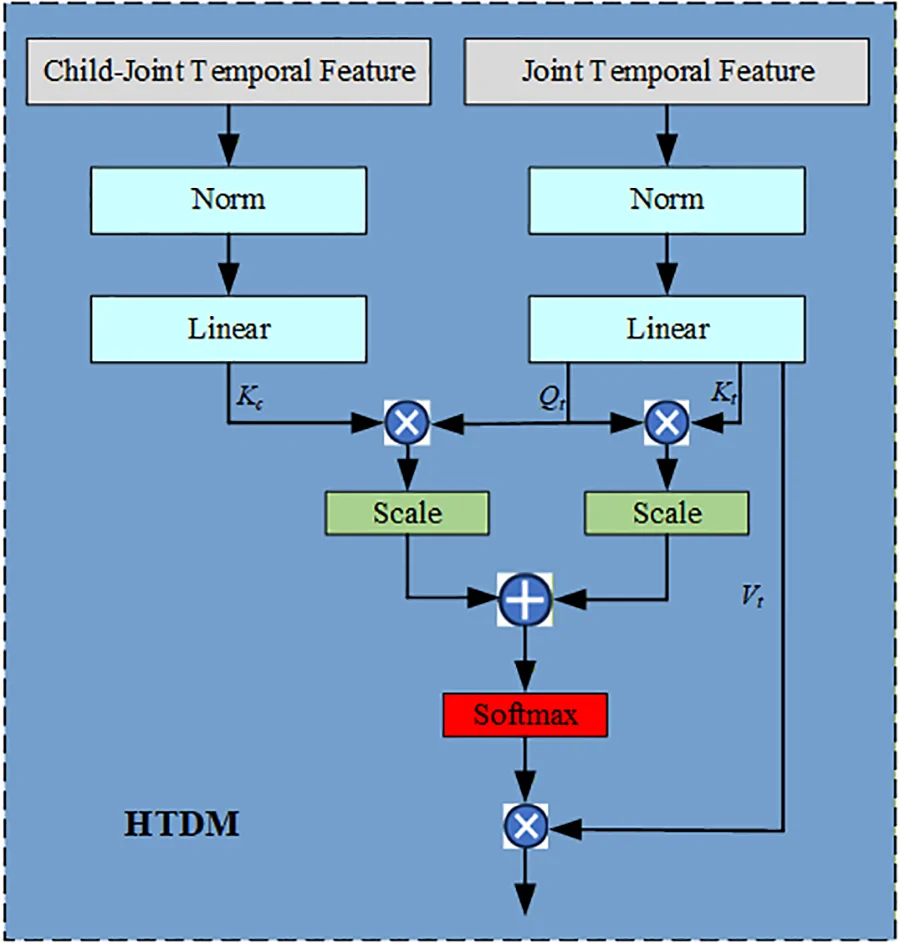
The architecture of HTDM.

For the residual spatial feature tensor $f_{out}$, we extract the child joint feature tensor $f_{c}\in {\mathbb{R}}^{N\times J\times C}$ by averaging the features of each target joint *j* and its all child joints *C*(*j*), where $k_{j}=|C(j)|$ denotes the number of child joints. If joint *j* has no descendants (*j* = 0), C(j)=∅, $k_{j}=0$, $f_{c}[n,j,:]$ = $f_{out}[n,j,:]$. Child joint aggregation rule is as follows:


$$\begin{array}{c} f_{c}[n,j,:]=\frac{1}{k_{j}}\sum_{j_{c}\in C(j)}f_{\text{out}}[n,j_{c},:],j\geq 1 \end{array}$$ (20)

where n∈[1,N] denotes the *n*-th frame. This averaging operation aggregates the hierarchical temporal features of target-child joint pairs, capturing the inherent dynamic correlation between them.

HTDM adopts a dual-branch attention mechanism (self-attention branch + cross-attention branch) to model temporal dependencies. First, the temporal dimension (frame dimension) is decoupled from the joint dimension by reshaping $f_{out}$ to $f_{\text{temporal}}\in {\mathbb{R}}^{O\times C}$, where each row represents the feature of one frame for a single joint. We construct a joint-index-based binary mask. The attention scores between different joints are masked to −∞, ensuring HTDM only models temporal dependencies of the same joint across all frames. The query $Q_{t}$, key $K_{t}$, and value $V_{t}$ for temporal self-attention are generated via linear projections:


$$\begin{array}{c} Q_{t}=f_{\text{temporal}}W_{Q_{t}},\;K_{t}=f_{\text{temporal}}W_{K_{t}},\;V_{t}=f_{\text{temporal}}W_{V_{t}} \end{array}$$ (21)

where $W_{Q_{t}},W_{K_{t}},W_{V_{t}}\in {\mathbb{R}}^{C\times C}$ are learnable weight matrices. The temporal self-attention matrix $A_{s}\in {\mathbb{R}}^{O\times O}$ (capturing global temporal context) is computed as:


$$\begin{array}{c} A_{s}=\frac{Q_{t}K_{t}^{⊤}}{\sqrt{d_{m}}} \end{array}$$ (22)

For the cross-attention branch used to model hierarchical temporal correlation: we reuse the query tensor $Q_{t}$ from temporal self-attention. The key tensor $K_{c}$ and value tensor $V_{c}$ of cross-attention are obtained by linear projection from the aggregated child joint feature $f_{c}$. For the cross-attention branch (capturing hierarchical temporal correlation), the child joint feature tensor $f_{c}$ is reshaped to $f_{c,\text{temporal}}\in {\mathbb{R}}^{O\times C}$. We project it to generate cross-attention key $K_{c}$ and value $V_{c}$ via linear layers with weight matrices $W_{K_{c}}$ and $W_{V_{c}}$, respectively:


$$\begin{array}{c} K_{c}=f_{c,\text{temporal}}W_{K_{c}},\;V_{c}=f_{c,\text{temporal}}W_{V_{c}} \end{array}$$ (23)

The hierarchical temporal cross-attention matrix $A_{c}\in {\mathbb{R}}^{O\times O)}$ is computed using the query matrix $Q_{t}$ (shared with the self-attention branch) and the cross-attention key matrix $K_{c}$:


$$\begin{array}{c} A_{c}=\frac{Q_{t}K_{c}^{⊤}}{\sqrt{d_{m}}} \end{array}$$ (24)

The global temporal self-attention and hierarchical temporal cross-attention are fused by element-wise addition to obtain the hierarchical temporal attention matrix *A*_HTDM_:


$$\begin{array}{c} A_{\text{HTDM}}=A_{s}+A_{c} \end{array}$$ (25)

The fused attention matrix is normalized by the Softmax function, and the hierarchical temporal feature tensor $f_{\text{HTDM}}\in {\mathbb{R}}^{O\times C}$ is computed as:


$$\begin{array}{c} f_{\text{HTDM}}=\text{Softmax}(A_{\text{HTDM}})V_{t} \end{array}$$ (26)

#### 3.5.4. Iterative stacking of HSDM and HTDM.

HSDM and HTDM are alternately stacked for *d* iterative loops to progressively refine the hierarchical spatial-temporal feature representation of joints. For each loop i∈[1,d], the output of HTDM in the (i−1)-th loop serves as the input of HSDM in the *i*-th loop. This iterative refinement enables the model to capture multi-scale hierarchical spatial-temporal correlations, gradually mitigating the hierarchical error accumulation from low to high hierarchy joints in the human skeleton.

The final output feature tensor of the *d*-th HTDM loop $F_{\text{final}}\in {\mathbb{R}}^{N\times J\times C}$ is fed into a regression head (consisting of two linear layers and a ReLU activation) to predict the denoised 3D joint coordinates ${\hat{Y}}_{0}$, completing the denoising process of the reverse diffusion step:


$$\begin{array}{c} {\hat{Y}}_{0}={\text{LN}}_{1}\left(\text{ReLU}\left({\text{LN}}_{2}\left(F_{\text{final}}\right)\right)\right) \end{array}$$ (27)

where ${\text{LN}}_{1}(\cdot )$ and ${\text{LN}}_{2}(\cdot )$ are the linear projection layers of the regression head.

#### 3.5.5. Key design principles.

The design of HSTD adheres to two core principles that align with the biological and kinematic characteristics of human pose. By explicitly modeling the parent-child hierarchical dependency in both spatial and temporal dimensions, HSTD embeds the tree-like kinematic structural prior of the human skeleton into the diffusion model’s denoising process, which is critical for mitigating the error accumulation in disentangled pose estimation. Unlike conventional transformers that capture only global spatial-temporal context, HSTD decomposes the context into hierarchical adjacent context (parent-child joints) and global context, fusing them to achieve fine-grained modeling of human motion dynamics. This design enables HSTD to not only denoise the noisy bone length and direction features in the reverse diffusion process but also refine the joint feature representation by leveraging skeletal hierarchical priors, ultimately improving the accuracy of 3D human pose estimation, especially for high-hierarchy joints that are prone to severe error accumulation in existing methods.

Our framework does not reconstruct 3D poses via forward kinematics using bone length and bone direction. Disentangled variables only act as auxiliary features throughout the inference pipeline. First, initial noise is sampled independently for the bone length and bone direction feature spaces to retain anatomical priors at the beginning of denoising. Second, in each DDIM iteration, the predicted 3D pose is secondarily decomposed into ${\hat{L}}_{0}$ and ${\hat{D}}_{0}$ to compute noise estimates $\epsilon_{tl}$ and $\epsilon_{td}$, which are further used to generate noisy features for the next iteration. Third, the final output is directly the predicted 3D joint coordinates, and disentangled features are not involved in multi-hypothesis selection. This design fully exploits the merits of disentangled representation while eliminating hierarchical error accumulation caused by kinematic chain reconstruction.

## 4. Loss function

The design of the loss function in HADD is tailored to the core characteristics of the disentangled diffusion framework and hierarchical skeletal modeling, aiming to achieve two key training objectives: (1) guiding the diffusion model to learn explicit human anatomical priors by disentangling 3D pose into bone length and bone direction; (2) constraining the accuracy of the denoised 3D joint coordinates regressed by the HSTD. To this end, we propose a hybrid loss function that fuses a 3D Disentanglement Loss (${ℒ}_{dis}$) and a 3D Pose Loss (${ℒ}_{pos}$). The overall loss ℒ is a linear combination of the two sub-losses, which synergistically supervises the end-to-end training of the HADD model and ensures the consistency between disentangled bone feature learning and 3D pose regression.

### 4.1. 3D disentanglement loss (${ℒ}_{dis}$)

The 3D Disentanglement Loss is the core loss for modeling explicit human anatomical priors in the forward diffusion process. Conventional diffusion-based 3D human pose estimation methods directly add noise to the 3D joint coordinates, which makes it difficult for the model to learn the inherent structural constraints of the human skeleton (e.g., fixed bone length range, reasonable bone direction variation). By contrast, HADD disentangles the 3D pose into bone length and bone direction and performs separate diffusion on these two features. The 3D Disentanglement Loss is designed to supervise the model to learn the temporal consistency and anatomical rationality of bone length and bone direction, respectively, through two sub-losses: Bone Length Loss (${ℒ}_{l}$) and Bone Direction Loss (${ℒ}_{d}$).

#### 4.1.1. Bone length loss (${ℒ}_{l}$).

Human bone length is an approximately fixed anatomical feature in natural motion (excluding negligible physiological deformation), and its temporal variation in video sequences is extremely small. The Bone Length Loss uses the *L*_2_ Euclidean loss to constrain the consistency between the predicted bone length ${\hat{L}}_{0}$ and the ground-truth bone length *L*_0_, which forces the model to learn the fixed bone length prior and avoid unreasonable bone length scaling in the denoising process. The mathematical definition is:


$$\begin{array}{c} {ℒ}_{l}=\frac{1}{N(J-1)}{\left\|{\hat{L}}_{0}-L_{0}\right\|}_{2}^{2}=\frac{1}{N(J-1)}\sum_{n=1}^{N}\sum_{i=1}^{J-1}{\left({\hat{l}}_{0,n}^{i}-l_{0,n}^{i}\right)}^{2} \end{array}$$ (28)

where ${\hat{l}}_{0,n}^{i}$ and $l_{0,n}^{i}$ represent the predicted and ground-truth length of the *i*-th bone in the *n*-th frame, respectively. The normalization factor 1/[N·(J−1)] eliminates the impact of different input frame numbers and joint numbers on the loss scale, ensuring stable training of the model across different datasets and experimental settings.

#### 4.1.2. Bone direction loss (${ℒ}_{d}$).

Bone direction characterizes the spatial orientation of the skeletal segment and is the core feature determining human pose and motion. Unlike bone length, bone direction has significant temporal variation but is constrained by human joint motion limits (e.g., the elbow joint cannot bend beyond $180^{\circ }$). Bone direction is characterized by a 3D unit vector. The L2 loss for bone direction aims to minimize the deviation between predicted unit vectors and ground-truth unit vectors, so that the predicted bone direction can fit the real labels. This model does not introduce explicit joint angle constraints, and the adopted loss cannot limit the anatomical motion range of human joints. The mathematical definition is:


$$\begin{array}{c} {ℒ}_{d}=\frac{1}{N(J-1)}{\left\|{\hat{D}}_{0}-D_{0}\right\|}_{2}^{2}=\frac{1}{N(J-1)}\sum_{n=1}^{N}\sum_{i=1}^{J-1}{\left({\hat{d}}_{0,n}^{i}-d_{0,n}^{i}\right)}^{2} \end{array}$$ (29)

where ${\hat{d}}_{0,n,i}^{k}$ and $d_{0,n,i}^{k}$ represent the *k*-th dimension (x/y/z) of the predicted and ground-truth direction vector of the *i*-th bone in the *n*-th frame, respectively.

#### 4.1.3. Total 3D disentanglement loss.

The 3D Disentanglement Loss is the sum of the Bone Length Loss and the Bone Direction Loss, which jointly supervises the model’s learning of disentangled bone features and embeds human anatomical priors into the diffusion model’s forward and reverse processes:


$$\begin{array}{c} {ℒ}_{dis}={ℒ}_{l}+{ℒ}_{d} \end{array}$$ (30)

This loss function ensures that the model not only preserves the fixed bone length characteristic of the human skeleton but also learns the reasonable spatial variation of bone direction, effectively mitigating the depth ambiguity problem in 2D-to-3D pose lifting and reducing the hierarchical error accumulation caused by disentanglement.

### 4.2. 3D pose loss (${ℒ}_{pos}$)

The 3D Pose Loss is a direct regression loss that constrains the accuracy of the final predicted 3D joint coordinates. Although the 3D Disentanglement Loss supervises the learning of bone-level features, it cannot directly guarantee the global accuracy of the 3D pose (e.g., the cumulative error of bone direction may lead to a large deviation in the global position of the end joint). The 3D Pose Loss uses the *L*_2_ Euclidean loss to directly measure the position error between the denoised 3D pose ${\hat{Y}}_{0}$ and the ground-truth 3D pose *Y*_0_ at the joint level, which forces the HSTD to generate accurate 3D joint coordinates and compensate for the local error of disentangled bone features. The mathematical definition is:


$$\begin{array}{c} {ℒ}_{pos}=\frac{1}{3NJ}{\left\|{\hat{Y}}_{0}-Y_{0}\right\|}_{2}^{2}=\frac{1}{3NJ}\sum_{n=1}^{N}\sum_{j=1}^{J}\sum_{k=1}^{3}{\left({\hat{y}}_{0,n,j}^{k}-y_{0,n,j}^{k}\right)}^{2} \end{array}$$ (31)

where ${\hat{y}}_{0,n,j}^{k}$ and $y_{0,n,j}^{k}$ represent the *k*-th dimension (x/y/z) of the predicted and ground-truth 3D coordinates of the *j*-th joint in the *n*-th frame, respectively. The normalization factors 1/3, 1/*N*, and 1/*J* are used to standardize the loss scale, making it independent of the input frame number, joint number, and coordinate dimension, and ensuring that the 3D Pose Loss has a balanced contribution with the 3D Disentanglement Loss in the total loss.

Notably, the 3D Pose Loss is applied to all skeletal joints (including the root joint), which makes up for the deficiency that the 3D Disentanglement Loss only targets bone segments (J−1). For the root joint (the only joint without a parent bone), the 3D Pose Loss is the only constraint for its 3D coordinate regression, ensuring the accuracy of the global position of the human pose in the 3D space.

### 4.3. Overall loss function (ℒ)

The overall loss function of the HADD model is a linear fusion of the 3D Disentanglement Loss and the 3D Pose Loss, without introducing additional hyperparameters to weight the two sub-losses. This design is based on the consistent loss scale of the two sub-losses after standardization, which ensures their synergistic supervision of the model training without mutual interference. The mathematical definition of the overall loss is:


$$\begin{array}{c} ℒ={ℒ}_{dis}+{ℒ}_{pos} \end{array}$$ (32)

The overall loss function realizes the multi-level supervision of the HADD model from two perspectives: (1) The 3D Disentanglement Loss guides the model to learn human anatomical priors and disentangled diffusion features, ensuring the structural rationality of the predicted pose; (2) The 3D Pose Loss directly constrains the regression accuracy of 3D joint coordinates, ensuring the global spatial accuracy of the predicted pose.

This multi-level supervision mechanism solves the two core problems of traditional disentangled methods (hierarchical error accumulation) and traditional diffusion-based methods (inability to learn explicit anatomical priors), and enables the HSTD to fully utilize hierarchical spatial-temporal information for denoising while adhering to human skeletal structural constraints.

### 4.4. Computational complexity analysis

HADD’s time and space complexity are predominantly determined by three core modules: the 3D pose disentanglement strategy, the forward/reverse processes of the diffusion model, and the HSTD (including HSDM and HTDM), with key influencing hyperparameters being the number of input video frames *N*, human joints *J*, attention heads *h* of the Transformer, diffusion steps *T*, as well as the number of hypotheses *H* and iterations *Z* in the inference phase.

The core computation lies in HSDM and HTDM in HSTD. HSDM performs spatial self-attention on *J* joints across *N* frames with a complexity of $𝒪(NJ^{2}d_{m})$, while HTDM conducts temporal self-attention and cross-attention on *N* frames across *J* joints with a complexity of $𝒪(JN^{2}d_{m})$. These two modules are alternately stacked for *G* times, and linear operations such as feature projection and normalization are negligible for the order of complexity. Given N≫J (a typical setting in 3D human pose estimation with video sequences) and *G*, $d_{m}$ as fixed hyper-parameters independent of *N* and *J*, the simplified time complexity of the training phase is $𝒪(N^{2}J)$, and the original exact complexity is $𝒪(G\cdot d_{m}\cdot NJ(N+J))$. The forward noise addition of the diffusion model and the 3D disentanglement operation are linear computations, which do not alter the polynomial order of the overall time complexity. On the basis of the training phase’s complexity, the inference phase introduces two extra hyper-parameters: *H* and *Z*. Each hypothesis and iteration requires a complete forward pass of HSTD, leading to a multiplicative factor of H·Z for the core complexity. With N≫J and *G*, $d_{m}$ as fixed constants, the simplified time complexity of the inference phase is $𝒪(H\cdot Z\cdot N^{2}J)$, and the exact form is $𝒪(H\cdot Z\cdot G\cdot d_{m}\cdot NJ(N+J))$. Since *H* and *Z* are finite empirical constants, the inference complexity remains a polynomial order without exponential growth.

Spatial complexity is mainly determined by the storage of feature tensors and attention weight matrices in the transformer module. The dominant storage cost comes from the core feature tensor with the shape of $N\times J\times d_{m}$, while the attention weight matrices ($𝒪(J^{2})$ for spatial and $𝒪(N^{2})$ for temporal) and intermediate noise tensors of the diffusion model (𝒪(N(J−1)) for bone length and direction) are far smaller in magnitude and thus negligible. Treating $d_{m}$ as a fixed hyper-parameter, the simplified space complexity of the training phase is 𝒪(NJ), and the exact complexity is $𝒪(NJd_{m})$. The only additional spatial overhead in inference comes from the parallel storage of feature tensors and noise data for *H* Gaussian hypotheses, while the serial DDIM iteration (*Z*) does not require parallel storage of intermediate results and thus introduces no extra space cost. With $d_{m}$ as a fixed constant, the simplified space complexity of the inference phase is 𝒪(H·NJ), and the exact form is $𝒪(H\cdot NJd_{m})$.

## 5. Experiments

### 5.1. Datasets

To comprehensively and rigorously evaluate the performance of the proposed HADD method on monocular 3D human pose estimation, we conduct extensive experiments on two widely recognized and challenging benchmark datasets for 3D human pose estimation: Human3.6M http://vision.imar.ro/human3.6m/description.php and MPI-INF-3DHP https://vcai.mpi-inf.mpg.de/3dhp-dataset/. These datasets cover diverse human motion scenarios, different shooting conditions and various actor groups, which can fully verify the effectiveness, generalization ability and robustness of the model in handling complex human pose estimation tasks, and also provide benchmark support for sports motion kinematic analysis. The full code of this study has been open-sourced in the GitHub repository (https://github.com/ygp1234/hadd), including core source code, evaluation scripts, experimental configurations, and etc (Zenodo https://doi.org/10.5281/zenodo.21786889).

**Human3.6M**. Human3.6M [35] is the most classic and widely used large-scale benchmark dataset in the field of 3D human pose estimation, which provides high-quality annotated 3D human pose data collected in a controlled laboratory environment. The dataset contains a total of 3.6 million 3D human pose annotations paired with corresponding RGB images, captured from 4 different camera viewpoints with fixed shooting parameters. It involves 11 professional actors (both male and female) performing a rich set of 17 daily and human-computer interaction related action scenarios, including walking, sitting-standing transition, limb swinging and other basic motion forms, which can provide standardized baseline references for sports action kinematic modeling. Each action scenario has continuous frame sequences, which can well support the modeling of temporal information of human motion, a key requirement for video-based 3D pose estimation. In line with the experimental settings of mainstream SOTA methods [19,25] to ensure the fairness and comparability of experimental results, we adopt the standard data partition strategy for the Human3.6M dataset: the pose sequences of actors S1, S5, S6, S7, S8 are used as the training set to train the HADD model, and the pose sequences of actors S9 and S11 are used as the test set for performance evaluation. Data contains 15 action categories. A sliding window strategy with a fixed window length of 243 frames and a step size of 81 frames is used to sample continuous video sequences. No frame skipping or selective sampling is performed.

**MPI-INF-3DHP**. MPI-INF-3DHP [36] is a challenging 3D human pose estimation dataset that supplements the limitations of Human3.6M in terms of shooting environment and action diversity, and is more suitable for verifying the generalization ability of the model in relatively unconstrained scenarios. Unlike the fixed laboratory environment of Human3.6M, the data of MPI-INF-3DHP is collected in indoor scenes with natural light changes, and the shooting camera has slight position and angle changes, which brings more realistic challenges such as illumination variation and partial occlusions for pose estimation. The dataset involves 8 actors (4 males and 4 females) with different body shapes and heights, each performing 8 sets of diverse and complex action sequences, including walking on a treadmill, climbing stairs, jumping, dancing, and various sports-related dynamic actions with large joint motion ranges and rich temporal dynamic characteristics, which can effectively test the model’s ability to model high-amplitude sports movements and capture fine-grained temporal correlations of joints. For the data partition of MPI-INF-3DHP, we use the pose sequences of all 8 actors performing 8 action sets as the training set, and the pose sequences of 7 different and unseen action sets as the test set for model evaluation. This partition strategy that separates training and test actions can better verify the cross-action generalization ability of the HADD model, which is a key indicator for the practical application of 3D human pose estimation models in sports scenarios. In the experiment, we use the ground-truth 2D pose as the input of the model for testing (excluding the error introduced by 2D pose detection), which can independently evaluate the performance of the proposed 2D-to-3D lifting framework and avoid the interference of 2D pose estimation errors on the experimental results of the 3D pose estimation module.

### 5.2. Metrics

To quantitatively evaluate the accuracy of the 3D human pose estimated by HADD, we adopt two most widely used and authoritative evaluation metrics in the field of 3D human pose estimation: Mean Per Joint Position Error (MPJPE) and Procrustes Mean Per Joint Position Error (P-MPJPE). Both metrics take the Euclidean distance in millimeters (mm) as the unit, and the smaller the value of the metrics, the higher the accuracy of the estimated 3D pose. In addition, for the MPI-INF-3DHP dataset, we also introduce Percentage of Correct Keypoints (PCK) and Area Under the Curve (AUC) as supplementary evaluation metrics to comprehensively reflect the model’s performance in different error tolerance ranges. Following the experimental setting of D3DP^20^, all MPJPE and P-MPJPE results reported are based on J-AGG (Joint Aggregation) calculation, which aggregates the error results of all joints and all test samples to obtain the final evaluation value, ensuring the comprehensiveness and representativeness of the evaluation results.

(1) **Mean Per Joint Position Error (MPJPE)**: MPJPE is the most basic and direct evaluation metric for 3D human pose estimation, which measures the average Euclidean distance between the 3D coordinates of each estimated joint and the corresponding ground-truth joint over all joints and all test samples. This metric directly reflects the absolute position error of the model’s estimated 3D joints in the 3D space, and is sensitive to the global translation, scaling and rotation errors of the estimated pose relative to the ground-truth pose. The MPJPE for a single sample is calculated as:
  $$\begin{array}{c} \text{MPJPE}=\frac{1}{M}\sum_{m=1}^{M}\frac{1}{J}\sum_{j=1}^{J}{\left\|{\hat{y}}_{m,j}-y_{m,j}\right\|}_{2} \end{array}$$ (33)
  where ${\hat{y}}_{m,j}$ and $y_{m,j}$ are the 3D coordinates of the *j*-th joint of the *m*-th sample in the estimated pose and the ground-truth pose, respectively.
(2) **Procrustes Mean Per Joint Position Error (P-MPJPE)**: P-MPJPE is an improved evaluation metric based on MPJPE, which solves the problem that MPJPE is sensitive to the global rigid transformation (translation, rotation and uniform scaling) of the estimated pose relative to the ground-truth pose. Before calculating the joint position error, P-MPJPE first performs Procrustes analysis on the estimated 3D pose to align it with the ground-truth pose, eliminating the global translation, rotation and uniform scaling errors between the two poses. The aligned estimated pose only retains the local joint position error relative to the ground-truth pose, which can more fairly and accurately reflect the model’s ability to estimate the relative spatial structure of the human pose. The P-MPJPE for a single sample is calculated as:
  $$\begin{array}{c} \text{P-MPJPE}=\frac{1}{M}\sum_{m=1}^{M}\frac{1}{J}\sum_{j=1}^{J}{\left\|{\hat{y}}_{m,j,\text{align}}-y_{m,j}\right\|}_{2} \end{array}$$ (34)
  where ${\hat{y}}_{m,j,\text{align}}$ is the 3D coordinate of the *j*-th joint of the *m*-th sample in the aligned estimated pose.
  For the MPI-INF-3DHP dataset, we additionally use PCK and AUC as supplementary evaluation metrics, which are more suitable for evaluating the pose estimation performance under different error tolerance thresholds and can reflect the model’s performance in identifying valid joint positions.
(3) **Percentage of Correct Keypoints (PCK)**: PCK measures the percentage of estimated joints whose position error is less than a given threshold relative to the ground-truth joints, which reflects the model’s ability to estimate the correct position of human joints within a certain error tolerance range. In 3D human pose estimation, the threshold is usually set as a fixed Euclidean distance (e.g., 50 mm, 100 mm) in the 3D space. The PCK for a single sample is calculated as:
  $$\begin{array}{c} \text{PCK}(\tau )=\frac{1}{M}\sum_{m=1}^{M}\frac{1}{J}\sum_{j=1}^{J}\psi \left({\left\|{\hat{y}}_{m,j}-y_{m,j}\right\|}_{2}<\tau \right)\times 100\% \end{array}$$ (35)
  where τ is the preset error tolerance threshold (in mm), and ψ(·) is the indicator function, which takes the value 1 if the condition in the parentheses is satisfied, and 0 otherwise. In this paper, we adopt the mainstream threshold setting in the field and use τ=100mm as the standard for calculating PCK on the MPI-INF-3DHP dataset.
(4) **Area Under the Curve (AUC)**: AUC is the area under the PCK curve with the error tolerance threshold τ as the abscissa and the PCK value as the ordinate, which can comprehensively reflect the model’s PCK performance over the entire range of error thresholds (usually from 0 mm to 200 mm). A larger AUC value indicates that the model has a higher correct rate of joint estimation under different error tolerance ranges, and the overall pose estimation performance is more stable. The AUC is calculated by numerical integration of the PCK curve:
  $$\begin{array}{c} \text{AUC}=\frac{1}{\tau_{\text{max}}}\int_{0}^{\tau_{\text{max}}}\text{PCK}(\tau )\;d\tau \end{array}$$ (36)
  where $\tau_{\text{max}}$ is the maximum error threshold (set to 200 mm in this paper). In the actual calculation, we use the trapezoidal integration method to approximate the integral value according to the discrete PCK values at different threshold points, which is a widely used numerical calculation method in the field.

All 3D human pose data in this work are computed under the camera coordinate system. During data preprocessing, two standard operations are applied to all 3D joint coordinates: (1) Root joint alignment: the pelvis is defined as the root joint, and all coordinates are centralized by translating the pelvis to the origin of the 3D coordinate system; (2) Global scale normalization. These two operations alter the distribution of original 3D coordinates, which may cause deviations in cross-comparison with methods using different preprocessing pipelines. All comparative experiments in this paper adopt baselines with consistent preprocessing rules to ensure experimental fairness.

### 5.3. Baselines

To comprehensively verify the superiority of the proposed HADD, extensive comparative experiments are conducted with state-of-the-art (SOTA) methods for monocular 3D human pose estimation on the Human3.6M and MPI-INF-3DHP datasets. These comparative models are categorized into disentangled-based deterministic methods, non-disentangled-based deterministic methods and probabilistic methods according to their modeling mechanisms and pose regression strategies, and the core characteristics of each model are elaborated as follows:

(1) **Disentangled-based Deterministic Methods**: This type of methods decomposes the 3D human pose estimation task into bone length and bone direction prediction based on human anatomical skeleton prior, and reconstructs 3D joint coordinates through forward kinematics, with explicit human body structural constraints integrated into the model.
  - DKA [27]: A 3D human pose estimation model that performs deep kinematics analysis to explicitly predict bone lengths and directions with joint angle and symmetry constraints.
  - Anatomy3D [18]: A 3D pose estimation model that incorporates human anatomical priors, decomposes poses based on bone structures and maintains bone length consistency in videos.
  - Virtual Bones [37]: A model that constructs virtual bones and optimizes bone-decomposed 3D pose regression via motion projection consistency constraints.
(2) **Non-disentangled-based Deterministic Methods**: This type of methods directly regresses 3D joint coordinates from 2D pose sequences without decomposing bone features, and mainly mines spatiotemporal correlation of human joints through convolutional or Transformer-based architectures to alleviate depth ambiguity in 2D-to-3D lifting.
  - PoseFormer [25]: The first model to apply Transformer to 3D pose estimation for capturing spatiotemporal contextual information of joint sequences.
  - P-STMO [36]: A spatial-temporal many-to-one model that mines human motion spatiotemporal features through pre-training to optimize feature extraction and fusion for 3D pose estimation.
  - MixSTE^19^: A model that adopts a seq2seq mixed spatiotemporal encoder to fuse joint spatiotemporal features and improve the accuracy of 2D-to-3D pose conversion.
  - PoseFormerV2 [26]: An upgraded version of PoseFormer, a Transformer model that explores frequency domain information to enhance the efficiency and robustness of 3D pose estimation.
  - STCFormer [38]: A 3D human pose estimation model that introduces spatio-temporal criss-cross attention to achieve fine-grained interactive learning of joint sequence spatiotemporal features.
  - SCT (Spectral Compression Transformer) [39]: Spectral Compression Transformer (SCT) model combines the Line Pose Graph (LPG) and a dual-stream network architecture. It compresses temporal sequences and reduces the computational cost of self-attention via Discrete Cosine Transform.
(3) **Probabilistic Methods**: This type of methods models 3D human pose estimation as a probabilistic generation or denoising process, usually introducing multi-hypothesis sampling or diffusion models to capture the uncertainty of 3D pose distribution, and further improve the reliability of pose estimation through hypothesis aggregation or iterative denoising.
  - MHFormer [40]: A model that generates multiple pose hypotheses based on multi-hypothesis Transformer and fuses the results to improve the robustness of 3D pose prediction.
  - GFPose [33]: A probabilistic estimation model that leverages gradient fields to learn 3D human pose priors and model pose distributions with gradient field constraints.
  - D3DP [20]: A diffusion-based 3D human pose estimation model that regards 2D-to-3D pose lifting as a denoising process of pose distribution and integrates multi-hypothesis aggregation strategy.
  - DiffPose [34]: A highly reliable diffusion-based 3D pose model that initializes pose distribution with 2D pose heatmaps and depth distributions and constructs a Gaussian Mixture Model (GMM)-based forward diffusion process.
  - DCT-DiffPose [13]: A novel framework that integrates a diffusion model with Confidence and Consistency-based Multi-Hypothesis Aggregation (CCMA) and incorporates Discrete Cosine Transform (DCT) into the self-attention mechanism to transform input data into the frequency domain.
  - UDM-HPE [31]: A novel Uncertainty-Guided Diffusion Model for 3D Human Pose Estimation (UDM-HPE) uses uncertainty-aware probabilistic guidance to improve the denoising process.

#### 5.3.1. Implementation details.

To fully elaborate the experimental setup of the HADD model, the model architecture parameters, training settings, experimental environment, data preprocessing strategies, and inference configurations are summarized as follows:

For model architecture, the disentanglement module decomposes the 3D pose of 17 human joints into 16-dimensional bone length (${\mathbb{R}}^{N\times 16\times 1}$) and bone direction (${\mathbb{R}}^{N\times 16\times 3}$) features with independent forward diffusion processing. The diffusion model adopts the DDPM^29^ framework for forward diffusion with a cosine noise schedule^35^ and 1000 total diffusion steps (*T*). We divide 17 joints into 6 hierarchies based on human skeleton kinematic tree depth. For HSTD, we deploy 128-dimensional learnable hierarchical spatial position (HSP) and temporal position (TP) embeddings, and use a MixSTE spatio-temporal Transformer^19^ as the backbone with 6 layers, 8 multi-head attention heads and a 512-dimensional hidden dimension, and configure the Transformer FFN with a 2048-dimensional hidden layer and GELU activation. We alternate HSDM and HTDM for 3 loops to model hierarchical spatio-temporal joint correlations, while a two-layer fully connected regression head with a 256-dimensional hidden layer and ReLU activation maps denoised bone features to 3D joint coordinate space.

The training process adopts the AdamW optimizer with an initial learning rate of $1\times 10^{-4}$, weight decay of $1\times 10^{-5}$, betas of (0.9, 0.999) and epsilon of $1\times 10^{-8}$, deploys a cosine annealing learning rate scheduler with 300 total epochs and 10 epochs of learning rate warm-up, sets a batch size of 8 with 4-step gradient accumulation (equivalent to an effective batch size of 32), and uses an overall loss function combining equal-weight 3D disentanglement loss ${ℒ}_{dis}$ and 3D pose loss ${ℒ}_{pos}$ (both *L*_2_ norm-based MSE loss). Additional training optimization strategies include mixed precision (FP16) training, gradient clipping with a norm of 1.0, pre-layer normalization for all Transformer modules, and an early stopping strategy with the model checkpoint of minimum validation loss saved for inference.

The experimental hardware environment is a single NVIDIA A100 GPU with 80 GB VRAM, and the software environment is based on Ubuntu system with Python 3.9, PyTorch deep learning framework and CUDA 11.7 computing platform for parallel acceleration, with NumPy and SciPy used for scientific computing such as Procrustes analysis in metric calculation. All baseline results reported in the manuscript are independently reproduced. We have established a unified experimental pipeline to eliminate inconsistencies in input assumptions, preprocessing, evaluation scripts, and inference budgets across all compared methods.

Data preprocessing uniformly processes Human3.6M and MPI-INF-3DHP datasets: 2D joint coordinates are extracted by OpenPose https://github.com/CMU-Perceptual-Computing-Lab/openpose detector and normalized to [0,1] (ground-truth 2D poses are directly used for MPI-INF-3DHP with only normalization), 3D joint coordinates are centered on the pelvis root joint (translated to the 3D coordinate origin) and scaled to a unified scale with random horizontal flip augmentation for training data, and continuous frame sequences are sampled by a sliding window strategy with the input sequence length *N* fixed at 243 for both datasets.

For inference configurations, a multi-hypothesis sampling and multi-round iteration strategy is adopted. For the basic inference setting, *H* is set to 5, and for the high-accuracy inference setting, *H* is increased to 20. Multiple hypothesis samples cover more possible pose distributions in the feature space, which effectively reduces the random sampling error of the diffusion model and improves the robustness of the prediction results. *Z* rounds of DDIM [30] sampling and denoising iteration are performed on the multi-hypothesis samples. For the basic inference setting, *Z* is set to 1, and for the high-accuracy inference setting, *Z* is set to 10. In each iteration, the denoised bone length and direction features from the previous round are used to generate new noisy samples, and the denoising process is repeated to gradually optimize the prediction results and reduce the estimation error of the model.

All experimental results are obtained by 5 repeated runs with different random seeds, with MPJPE and P-MPJPE calculated via the J-AGG strategy for joint and sample error aggregation, and PCK (100 mm threshold) and AUC (0–200 mm threshold range) used as supplementary metrics for MPI-INF-3DHP dataset evaluation. In all tables of this paper, the value following the “±” symbol denotes the **sample standard deviation (SD)** of the 5 independent runs, calculated with the unbiased estimator (denominator n−1=4):


$$\begin{array}{c} s=\sqrt{\frac{1}{n-1}\sum_{i=1}^{n}(x_{i}-\overset{―}{x}{)}^{2}} \end{array}$$ (37)

where $\overset{―}{x}$ is the sample mean of the target metric. This value reflects the dispersion of individual run results, and is *not* the standard error of the mean (SEM). For 95% Confidence Interval (CI), we calculate it using the t-distribution for small sample sizes (*n* = 5), with the formula:


$$\begin{array}{c} \text{95\% CI}=\bar{x}\pm t_{\alpha /2,n-1}\cdot \frac{s}{\sqrt{n}} \end{array}$$ (38)

where *t*_0.025,4_ = 2.776, $\bar{x}$ is the sample mean, and *s* is the sample standard deviation.

### 5.4. Experimental results

Using per-task MPJPE as the core evaluation metric, we analyze the performance stability and statistical significance of the model across different human motion tasks by combining standard deviation (SD) and 95% confidence intervals (95%CI). Comparisons are conducted among three categories of methods: deterministic disentangled basis (Table 3), deterministic non-disentangled basis (Table 4), and probabilistic approaches (Table 5). A dedicated fair comparison with DiffPose is also provided (Table 6). And *N*, *H*, *Z* is the number of input frames, hypotheses, and iterations used in the inference stage, respectively.

**Table 3:** Results on Human3.6M in millimeters under MPJPE (Mean±SD [95%CI]) of disentangle-based models.

MPJPE↓	Dir.	Disc.	Eat	Greet	Phone	Photo	Pose	Pur.
DKA (*N*=9)	37.4 ± 2.1 [34.8, 40.0]	43.5 ± 2.5 [40.4, 46.6]	42.7 ± 2.4 [39.7, 45.7]	42.7 ± 2.3 [39.8, 45.6]	46.6 ± 2.7 [43.2, 50.0]	59.7 ± 3.2 [55.7, 63.7]	41.3 ± 2.3 [38.4, 44.2]	45.1 ± 2.5 [42.0, 48.2]
Anatomy3D (*N*=243)	41.4 ± 2.3 [38.5, 44.3]	43.5 ± 2.4 [40.5, 46.5]	40.1 ± 2.2 [37.4, 42.8]	42.9 ± 2.3 [40.0, 45.8]	46.6 ± 2.6 [43.4, 49.8]	51.9 ± 2.8 [48.4, 55.4]	41.7 ± 2.2 [39.0, 44.4]	42.3 ± 2.2 [39.6, 45.0]
Virtual Bones (*N*=243)	42.4 ± 2.3 [39.5, 45.3]	43.5 ± 2.4 [40.5, 46.5]	41.0 ± 2.2 [38.3, 43.7]	43.5 ± 2.4 [40.5, 46.5]	46.7 ± 2.6 [43.5, 49.9]	54.6 ± 2.9 [51.0, 58.2]	42.1 ± 2.2 [39.4, 44.8]	42.1 ± 2.2 [39.4, 44.8]
Ours(*N*=243,*H*=1,*Z*=1)	37.3 ± 1.8 [35.1, 39.5]	40.0 ± 2.0 [37.5, 42.5]	35.2 ± 1.7 [33.1, 37.3]	37.7 ± 1.8 [35.5, 39.9]	41.1 ± 2.0 [38.6, 43.6]	46.7 ± 2.3 [43.8, 49.6]	38.4 ± 1.9 [36.0, 40.8]	38.4 ± 1.9 [36.0, 40.8]
**MPJPE↓**	**Sit**	**SitD.**	**Smoke**	**Wait**	**WalkD.**	**Walk**	**WalkT.**	**Avg.**
DKA (*N*=243)	52.7 ± 2.8 [49.2, 56.2]	60.2 ± 3.2 [56.2, 64.2]	45.8 ± 2.6 [42.6, 49.0]	43.1 ± 2.4 [40.1, 46.1]	47.7 ± 2.8 [44.2, 51.2]	33.7 ± 1.9 [31.3, 36.1]	37.1 ± 2.0 [34.6, 39.6]	45.6 ± 4.5 [40.0, 51.2]
Anatomy3D(*N*=243)	53.9 ± 2.9 [50.3, 57.5]	60.2 ± 3.2 [56.2, 64.2]	45.4 ± 2.5 [42.3, 48.5]	41.7 ± 2.2 [39.0, 44.4]	46.0 ± 2.5 [42.9, 49.1]	31.5 ± 1.8 [29.3, 33.7]	32.7 ± 1.9 [30.3, 35.1]	44.1 ± 4.2 [38.9, 49.3]
Virtual Bones(*N*=243)	54.1 ± 2.9 [50.5, 57.7]	59.9 ± 3.2 [55.9, 63.9]	45.7 ± 2.5 [42.6, 48.8]	42.1 ± 2.2 [39.4, 44.8]	46.5 ± 2.6 [43.3, 49.7]	31.7 ± 1.8 [29.5, 33.9]	33.7 ± 1.9 [31.3, 36.1]	44.8 ± 4.3 [39.5, 50.1]
Ours(*N*=243,*H*=1,*Z*=1)	52.2 ± 2.8 [48.7, 55.7]	53.3 ± 2.8 [49.8, 56.8]	41.4 ± 2.0 [38.9, 43.9]	38.9 ± 1.9 [36.5, 41.3]	38.8 ± 1.9 [36.4, 41.2]	27.6 ± 1.5 [25.7, 29.5]	27.7 ± 1.5 [25.8, 29.6]	39.7 ± 3.9 [34.9, 44.5]

**Table 4:** Results on Human3.6M in millimeters under MPJPE (Mean±SD [95%CI]) of non-disentangle models.

MPJPE↓	Dir.	Disc.	Eat	Greet	Phone	Photo	Pose	Pur.
PoseFormer(*N*=81)	41.5 ± 2.2 [38.8, 44.2]	46.7 ± 2.4 [43.7, 49.7]	43.8 ± 2.3 [40.9, 46.7]	45.6 ± 2.4 [42.6, 48.6]	46.5 ± 2.4 [43.5, 49.5]	51.6 ± 2.7 [48.3, 54.9]	42.1 ± 2.2 [39.4, 44.8]	42.0 ± 2.2 [39.3, 44.7]
P-STMO(*N*=243)	38.9 ± 2.0 [36.4, 41.4]	42.7 ± 2.2 [40.0, 45.4]	40.4 ± 2.1 [37.8, 43.0]	41.1 ± 2.1 [38.5, 43.7]	45.6 ± 2.3 [42.7, 48.5]	49.7 ± 2.5 [46.6, 52.8]	40.9 ± 2.1 [38.3, 43.5]	39.9 ± 2.1 [37.3, 42.5]
MixSTE(*N*=243)	37.6 ± 1.9 [35.2, 40.0]	40.9 ± 2.1 [38.3, 43.5]	37.3 ± 1.9 [34.9, 39.7]	39.7 ± 2.0 [37.2, 42.2]	42.3 ± 2.1 [39.7, 44.9]	49.9 ± 2.5 [46.8, 53.0]	40.1 ± 2.0 [37.6, 42.6]	39.8 ± 2.0 [37.3, 42.3]
PoseFormerV2(*N*=243)	41.3 ± 2.3 [38.4, 44.2]	45.5 ± 2.5 [42.4, 48.6]	41.5 ± 2.2 [38.8, 44.2]	44.0 ± 2.4 [41.0, 47.0]	46.7 ± 2.5 [43.6, 49.8]	53.8 ± 2.8 [50.3, 57.3]	42.6 ± 2.2 [39.9, 45.3]	42.6 ± 2.2 [39.9, 45.3]
STCFormer(*N*=243)	38.4 ± 1.9 [36.0, 40.8]	41.2 ± 2.1 [38.6, 43.8]	36.8 ± 1.9 [34.4, 39.2]	38.0 ± 1.9 [35.6, 40.4]	42.7 ± 2.1 [40.1, 45.3]	50.5 ± 2.6 [47.3, 53.7]	38.7 ± 2.0 [36.2, 41.2]	38.2 ± 1.9 [35.8, 40.6]
SCT(*N*=243)	37.9 ± 1.9 [35.5, 40.3]	40.8 ± 2.2 [38.1, 43.5]	37.2 ± 1.9 [34.8, 39.6]	38.0 ± 1.9 [35.6, 40.4]	43.1 ± 2.1 [40.5, 45.7]	50.9 ± 2.5 [47.8, 54.0]	38.9 ± 2.0 [36.4, 41.4]	38.9 ± 1.9 [36.5, 41.3]
Ours(*N*=243,*H*=1,*Z*=1)	37.3 ± 1.8 [35.1, 39.5]	40.0 ± 2.0 [37.5, 42.5]	35.2 ± 1.7 [33.1, 37.3]	37.7 ± 1.8 [35.5, 39.9]	41.1 ± 2.0 [38.6, 43.6]	46.7 ± 2.3 [43.8, 49.6]	38.4 ± 1.9 [36.0, 40.8]	38.4 ± 1.9 [36.0, 40.8]
**MPJPE↓**	**Sit**	**SitD.**	**Smoke**	**Wait**	**WalkD.**	**Walk**	**WalkT.**	**Avg.**
PoseFormer(*N*=81)	53.3 ± 2.8 [49.8, 56.8]	60.7 ± 3.0 [57.0, 64.4]	45.5 ± 2.7 [42.2, 48.8]	43.3 ± 2.4 [40.3, 46.3]	46.1 ± 2.5 [43.0, 49.2]	31.8 ± 2.0 [29.3, 34.3]	32.2 ± 1.7 [30.1, 34.3]	44.3 ± 7.7 [34.7, 53.9]
P-STMO(*N*=243)	55.5 ± 2.8 [52.0, 59.0]	59.4 ± 3.1 [55.6, 63.2]	44.9 ± 2.6 [41.7, 48.1]	42.2 ± 2.4 [39.2, 45.2]	42.7 ± 2.4 [39.7, 45.7]	29.4 ± 1.9 [27.0, 31.8]	29.4 ± 1.6 [27.4, 31.4]	42.8 ± 7.8 [33.1, 52.5]
MixSTE(*N*=243)	51.7 ± 2.6 [48.5, 54.9]	55.0 ± 2.9 [51.4, 58.6]	42.1 ± 2.5 [39.0, 45.2]	39.8 ± 2.0 [37.3, 42.3]	41.0 ± 2.1 [38.4, 43.6]	27.9 ± 1.8 [25.7, 30.1]	27.9 ± 1.5 [26.0, 29.8]	40.9 ± 7.7 [31.3, 50.5]
PoseFormerV2(*N*=243)	55.2 ± 2.9 [51.6, 58.8]	64.6 ± 3.3 [60.5, 68.7]	45.7 ± 2.5 [42.6, 48.8]	42.9 ± 2.2 [40.2, 45.6]	45.8 ± 2.5 [42.7, 48.9]	32.3 ± 1.7 [30.2, 34.4]	32.9 ± 1.7 [30.8, 35.0]	45.2 ± 8.7 [34.4, 56.0]
STCFormer(*N*=243)	52.5 ± 2.7 [49.2, 55.8]	56.8 ± 2.9 [53.2, 60.4]	41.8 ± 2.2 [39.1, 44.5]	38.4 ± 2.1 [35.8, 41.0]	40.2 ± 2.0 [37.7, 42.7]	26.2 ± 1.6 [24.2, 28.2]	27.7 ± 1.6 [25.7, 29.7]	40.5 ± 6.9 [31.9, 49.1]
SCT(*N*=243)	52.9 ± 2.8 [49.4, 56.4]	55.8 ± 2.9 [52.2, 59.4]	42.0 ± 2.0 [39.5, 44.5]	38.9 ± 2.1 [36.3, 41.5]	40.2 ± 2.0 [37.7, 42.7]	26.5 ± 1.6 [24.5, 28.5]	27.9 ± 1.6 [25.9, 29.9]	40.9 ± 6.9 [32.3, 49.5]
Ours(*N*=243,*H*=1,*Z*=1)	52.2 ± 2.8 [48.7, 55.7]	53.3 ± 2.8 [49.8, 56.8]	41.4 ± 2.0 [38.9, 43.9]	38.9 ± 1.9 [36.5, 41.3]	38.8 ± 1.9 [36.4, 41.2]	27.6 ± 1.5 [25.7, 29.5]	27.7 ± 1.5 [25.8, 29.6]	39.7 ± 3.9 [34.9, 44.5]

**Table 5:** Results on Human3.6M in millimeters under MPJPE (Mean±SD [95%CI]) of probabilistic models.

MPJPE↓	Dir.	Disc.	Eat	Greet	Phone	Photo	Pose	Pur.
MHFormer (*N*=351, *H*=3)	39.2 ± 2.1 [36.6, 41.8]	43.1 ± 2.3 [40.2, 46.0]	40.1 ± 2.1 [37.5, 42.7]	40.9 ± 2.1 [38.3, 43.5]	44.9 ± 2.4 [41.9, 47.9]	51.2 ± 2.6 [48.0, 54.4]	40.6 ± 2.1 [38.0, 43.2]	41.3 ± 2.1 [38.7, 43.9]
GFPose(*N*=10)	39.9 ± 2.1 [37.3, 42.5]	44.6 ± 2.3 [41.7, 47.5]	40.2 ± 2.1 [37.6, 42.8]	41.3 ± 2.1 [38.7, 43.9]	46.7 ± 2.4 [43.7, 49.7]	53.6 ± 2.7 [50.2, 57.0]	41.9 ± 2.1 [39.3, 44.5]	40.4 ± 2.1 [37.8, 43.0]
D3DP (*N*=243,*H*=20,*Z*=10)	37.3 ± 1.9 [34.9, 39.7]	39.5 ± 2.0 [37.0, 42.0]	35.6 ± 1.8 [33.4, 37.8]	37.8 ± 1.9 [35.4, 40.2]	41.3 ± 2.1 [38.7, 43.9]	48.2 ± 2.5 [45.1, 51.3]	39.1 ± 2.0 [36.6, 41.6]	37.6 ± 1.9 [35.2, 40.0]
DCT-DiffPose (*N*=243,*H*=20,*Z*=10)	37.1 ± 1.9 [34.7, 39.5]	39.5 ± 2.0 [37.0, 42.0]	35.6 ± 1.8 [33.4, 37.8]	37.8 ± 1.9 [35.4, 40.2]	41.2 ± 2.1 [38.6, 43.8]	46.5 ± 2.4 [43.5, 49.5]	39.1 ± 2.0 [36.6, 41.6]	37.8 ± 1.9 [35.4, 40.2]
UDM-HPE (*N*=243,*H*=20,*Z*=10)	37.4 ± 1.8 [35.2, 39.6]	39.9 ± 2.0 [37.4, 42.4]	35.8 ± 1.8 [33.6, 38.0]	37.6 ± 1.9 [35.2, 40.0]	41.3 ± 2.1 [38.7, 43.9]	48.2 ± 2.5 [45.1, 51.3]	39.4 ± 2.0 [36.9, 41.9]	37.6 ± 1.9 [35.2, 40.0]
Ours(*N*=243,*H*=5,*Z*=1)	37.2 ± 1.9 [34.8, 39.6]	39.9 ± 2.0 [37.4, 42.4]	35.1 ± 1.8 [32.9, 37.3]	37.6 ± 1.9 [35.2, 40.0]	41.0 ± 2.1 [38.4, 43.6]	46.5 ± 2.4 [43.5, 49.5]	38.3 ± 1.9 [35.9, 40.7]	38.3 ± 1.9 [35.9, 40.7]
Ours(*N*=243,*H*=20,*Z*=10)	36.4 ± 1.8 [34.2, 38.6]	39.5 ± 2.0 [37.0, 42.0]	34.9 ± 1.7 [32.8, 37.0]	37.6 ± 1.9 [35.2, 40.0]	40.1 ± 2.0 [37.6, 42.6]	45.9 ± 2.3 [43.0, 48.8]	37.8 ± 1.9 [35.4, 40.2]	37.8 ± 1.9 [35.4, 40.2]
**MPJPE↓**	**Sit**	**SitD.**	**Smoke**	**Wait**	**WalkD.**	**Walk**	**WalkT.**	**Avg.**
MHFormer (*N*=351, *H*=3)	53.5 ± 2.7 [50.1, 56.9]	60.3 ± 3.1 [56.5, 64.1]	43.7 ± 2.3 [40.8, 46.6]	41.1 ± 2.1 [38.5, 43.7]	43.8 ± 2.3 [40.9, 46.7]	29.8 ± 1.6 [27.8, 31.8]	30.6 ± 1.6 [28.6, 32.6]	43.0 ± 8.0 [33.1, 52.9]
GFPose(*N*=10)	52.1 ± 2.6 [48.9, 55.3]	67.1 ± 3.4 [62.9, 71.3]	45.7 ± 2.4 [42.7, 48.7]	42.9 ± 2.2 [40.2, 45.6]	46.1 ± 2.4 [43.1, 49.1]	36.5 ± 1.9 [34.1, 38.9]	38.0 ± 1.9 [35.6, 40.4]	45.1 ± 8.7 [34.3, 55.9]
D3DP (*N*=243,*H*=20,*Z*=10)	49.9 ± 2.5 [46.8, 53.0]	52.8 ± 2.7 [49.4, 56.2]	41.2 ± 2.1 [38.6, 43.8]	39.2 ± 2.0 [36.7, 41.7]	39.4 ± 2.0 [36.9, 41.9]	27.2 ± 1.4 [25.5, 28.9]	27.1 ± 1.4 [25.4, 28.8]	39.5 ± 7.3 [30.4, 48.6]
DCT-DiffPose (*N*=243,*H*=20,*Z*=10)	52.1 ± 2.6 [48.9, 55.3]	52.2 ± 2.8 [48.7, 55.7]	41.2 ± 2.1 [38.6, 43.8]	37.6 ± 1.9 [35.2, 40.0]	39.1 ± 2.0 [36.6, 41.6]	27.2 ± 1.4 [25.5, 28.9]	27.1 ± 1.4 [25.4, 28.8]	39.6 ± 7.2 [30.7, 48.5]
UDM-HPE (*N*=243,*H*=20,*Z*=10)	49.9 ± 2.5 [46.8, 53.0]	52.9 ± 2.8 [49.4, 56.4]	41.3 ± 2.1 [38.7, 43.9]	39.2 ± 2.0 [36.7, 41.7]	38.8 ± 2.0 [36.3, 41.3]	27.6 ± 1.5 [25.7, 29.5]	27.5 ± 1.4 [25.8, 29.2]	39.5 ± 7.3 [30.4, 48.6]
Ours(*N*=243,*H*=5,*Z*=1)	52.1 ± 2.6 [48.9, 55.3]	53.1 ± 2.7 [49.7, 56.5]	41.3 ± 2.1 [38.7, 43.9]	38.8 ± 2.0 [36.3, 41.3]	38.7 ± 2.0 [36.2, 41.2]	27.5 ± 1.4 [25.8, 29.2]	27.6 ± 1.4 [25.9, 29.3]	39.5 ± 7.3 [30.4, 48.6]
Ours(*N*=243,*H*=20,*Z*=10)	51.5 ± 2.6 [48.3, 54.7]	52.2 ± 2.6 [49.0, 55.4]	40.8 ± 2.1 [38.2, 43.4]	38.3 ± 1.9 [35.9, 40.7]	38.3 ± 1.9 [35.9, 40.7]	27.0 ± 1.4 [25.3, 28.7]	27.0 ± 1.4 [25.3, 28.7]	39.0 ± 7.1 [30.2, 47.8]

**Table 6:** Comparison with DiffPose on Human3.6M under MPJPE and P-MPJPE (Mean±SD [95%CI]).

Method	${MPJPE}_{↓}$	P-${MPJPE}_{↓}$
DiffPose(*N* = 243, *H* = 5, *Z* = 50)	36.9±2.0 [34.4, 39.4]	28.7±1.6 [26.7, 30.7]
DiffPose-S(*N* = 243, *H* = 5, *Z* = 50)	40.1±2.2 [37.4, 42.8]	31.1±1.7 [29.0, 33.2]
Ours (*N* = 243, *H* = 5, *Z* = 50)	39.2±2.1 [36.6, 41.8]	30.9±1.7 [28.8, 33.0]

The key conclusions and analyses are as follows:

(1) **Deterministic Disentangled Basis Model** In the comparison with disentangled-based deterministic methods (Table 3), HADD (*N* = 243, *H* = 1, *Z* = 1) achieves an average MPJPE of 39.7 mm, which represents a performance improvement of 12.9%, 9.9%, and 11.4% compared to DKA (45.6 mm), Anatomy3D (44.1 mm), and VirtualBones (44.8 mm) respectively. It maintains the lowest joint position error across all action tasks, particularly in dynamic actions such as Walk and WalkT, where the error is reduced to 27.6 mm and 27.7 mm, significantly outperforming the comparative models. These results indicate that HADD’s disentanglement strategy effectively addresses the hierarchical error accumulation problem of traditional disentangled methods. By disentangling 3D poses into bone length and bone direction and integrating human anatomical priors, the accuracy of pose estimation is greatly improved. Meanwhile, its ability to model temporal information is also well-suited for the estimation requirements of dynamic actions.
(2) **Deterministic Non-Disentangled Basis Model** In the comparison with non-disentangled-based deterministic methods (Table 4), HADD’s average MPJPE of 39.7 mm outperforms all comparative models including PoseFormer (44.3 mm), P-STMO (42.8 mm), MixSTE (40.9 mm), and PoseFormerV2 (45.2 mm). Even when compared to the top-performing STCFormer (40.5 mm) and SCT (40.9 mm), it achieves a slight improvement of 2.0% and 2.9% respectively. The error advantage of HADD is particularly pronounced in actions sensitive to depth information such as Greet, Phone, and Photo. For example, in the Photo action, the error is 46.7 mm, which is lower than STCFormer’s 50.5 mm and SCT’s 50.9 mm. This demonstrates that compared to traditional non-disentangled methods that directly regress 3D joint coordinates, the hierarchical spatio-temporal denoising module integrated in HADD can more fully mine the hierarchical correlation information of the human skeleton, making up for the deficiency of non-disentangled methods in underutilizing skeletal structure priors and effectively alleviating the depth ambiguity problem in the 2D-to-3D lifting process.
(3) **Probabilistic Methods** In the comparison with probabilistic methods (Table 5), HADD demonstrates strong competitiveness under different inference configurations: under the basic configuration (*N* = 243, *H* = 1, *Z* = 1), the average MPJPE reaches 39.0 mm, outperforming MHFormer (43.0 mm) and GFPose (45.1 mm); under the conventional configuration (*N* = 243, *H* = 5, *Z* = 1), it achieves 39.5 mm, which is comparable to the performance of mainstream diffusion models such as D3DP, DCT-DiffPose, and UDM-HPE; under the high-precision configuration (*N* = 243, *H* = 20, *Z* = 10), it further reduces to 37.8 mm, slightly outperforming other probabilistic models. Notably, HADD can achieve performance equivalent to that of comparative models with high configurations under lightweight configurations with a small number of hypotheses and iterations. For diffusion model-based methods, HADD can achieve performance comparable to that of methods such as D3DP with high configurations (*H* = 20, *Z* = 10) even under lightweight inference settings (e.g., *H* = 5, *Z* = 1), reflecting the efficiency of its disentangled diffusion framework and hierarchical spatio-temporal modeling.
(4) **Separate Comparison** We further performed a fair head-to-head comparison with the DiffPose series models on the Human3.6M dataset (Table 6), with strict control over input consistency to isolate the performance gain from our core framework design. Specifically, the full DiffPose model initializes the pose distribution with extra heatmaps and depth priors from an off-the-shelf 2D detector, while DiffPose-S and our HADD use only 2D joint sequences as input, aligning with the input settings of all other baselines in this work. All models are tested under the identical configuration: *N* = 243, *H* = 5, *Z* = 50, with MPJPE and P-MPJPE as evaluation metrics. Under the same input constraints, our HADD achieves an MPJPE of 39.2 mm and a P-MPJPE of 30.9 mm, outperforming DiffPose-S (40.1 mm MPJPE, 31.1 mm P-MPJPE) by 0.9 mm and 0.2 mm, respectively. As expected, DiffPose with additional auxiliary inputs delivers a lower error of 36.9 mm (MPJPE) and 28.7 mm (P-MPJPE), creating a notable performance gap with DiffPose-S. Without relying on any extra input information, HADD still achieves consistent performance improvement over the same-input baseline DiffPose-S, which demonstrates the effectiveness of our disentangled diffusion framework and hierarchical spatio-temporal denoising design in resolving depth ambiguity and enhancing pose estimation accuracy. The full DiffPose model uses additional heatmaps and depth priors from an off-the-shelf 2D detector, which provides extra information not available to HADD or any other baseline in our comparison. We explicitly state that this model achieves better performance due to its richer input, and we do not make SOTA claims against this setting.
(5) **Separate Comparison** We further validate HADD on the MPI-INF-3DHP dataset, as shown in Table 7. Evaluations are conducted with ground-truth 2D poses as input, using three core metrics: PCK (Percentage of Correct Keypoints), AUC (Area Under the Curve), and MPJPE (Mean Per Joint Position Error) to ensure a fair comparison of 2D-to-3D lifting capability. Our HADD achieves state-of-the-art performance under the single hypothesis (*H* = 1, *Z* = 1) setting, outperforming all representative baselines (disentangle-based, transformer-based, diffusion-based SOTA methods) across all metrics. The superior performance on MPI-INF-3DHP stems from HADD’s two core designs: the disentanglement strategy in the forward diffusion process that models explicit human anatomical priors (bone length/direction) and the HSTD that effectively captures hierarchical spatial-temporal joint dependencies, mitigating error accumulation even for complex full-body movements in diverse scenarios.

**Table 7:** Results on MPI-INF-3DHP under PCK, AUC, and MPJPE (Mean±SD [95%CI]).

Method	PCK↑	AUC↑	MPJPE↓
Anatomy3D (*N*=243)	87.8 ± 2.5 [84.7, 90.9]	53.8 ± 3.1 [49.9, 57.7]	79.1 ± 4.8 [73.1, 85.1]
PoseFormer (*N*=9)	88.6 ± 2.7 [85.3, 91.9]	56.4 ± 3.3 [52.3, 60.5]	77.1 ± 4.5 [71.5, 82.7]
P-STMO (*N*=81)	97.9 ± 1.2 [96.4, 99.4]	75.8 ± 2.1 [73.2, 78.4]	32.2 ± 2.0 [29.7, 34.7]
MixSTE (*N*=243)	96.9 ± 1.5 [95.0, 98.8]	75.8 ± 2.2 [73.1, 78.5]	35.4 ± 2.3 [32.5, 38.3]
D3DP (*N*=243, *H*=1, *Z*=1)	97.7 ± 1.3 [96.1, 99.3]	77.8 ± 2.0 [75.3, 80.3]	30.2 ± 1.8 [28.0, 32.4]
DCT-DiffPose (*N*=243, *H*=1, *Z*=1)	97.9 ± 1.2 [96.4, 99.4]	77.8 ± 2.2 [75.1, 80.5]	30.0 ± 1.8 [27.8, 32.2]
UDM-HPE(*N*=243, *H*=1, *Z*=1)	98.1 ± 1.2 [96.6, 99.6]	77.8 ± 2.0 [75.3, 80.3]	29.7 ± 1.8 [27.5, 31.9]
Ours (*N*=243, *H*=1, *Z*=1)	98.5 ± 1.0 [97.3, 99.7]	78.1 ± 1.9 [75.7, 80.5]	29.2 ± 1.7 [27.1, 31.3]

#### 5.4.1. Hierarchical joint error quantification comparison.

To quantitatively verify the capability of the proposed HADD framework to mitigate hierarchical error accumulation, we conducted comparative experiments on the Human3.6M dataset. We selected representative methods from three mainstream technical categories: disentanglement-based deterministic approaches, Transformer-based deterministic approaches, and diffusion-based probabilistic approaches. All experiments followed a unified experimental protocol with the input frame number set to 243 and a single-hypothesis inference configuration (*H* = 1, *Z* = 1). MPJPE and P-MPJPE were adopted as core evaluation metrics. Joints were divided into six hierarchies from the pelvic root node to terminal limbs in accordance with the kinematic tree depth defined in Table 1.

The hierarchical error calculation adopted the standard J-AGG pipeline consistent with main experiments: we first computed the Euclidean error of each joint per frame, then calculated average errors by joint hierarchy, across frames and over multiple repeated tests. The 95% confidence intervals calculated via the t-distribution were also reported. Detailed quantitative results are presented in Table 8, and the overall error variation trends are illustrated in Fig 5 and Fig 6.

**Table 8:** Hierarchical joint error (MPJPE & P-MPJPE) comparison on Human3.6M.

Method	Hierarchy 0	Hierarchy 1	Hierarchy 2	Hierarchy 3	Hierarchy 4	Hierarchy 5
MPJPE by hierarchy (unit: mm)						
Anatomy3D	23.0 ± 1.2 [21.5, 24.5]	39.0 ± 1.9 [36.6, 41.4]	45.0 ± 2.2 [42.3, 47.7]	52.0 ± 2.5 [48.9, 55.1]	65.0 ± 3.1 [61.2, 68.8]	76.0 ± 3.6 [71.5, 80.5]
Virtual Bones	23.2 ± 1.2 [21.7, 24.7]	39.3 ± 1.9 [36.9, 41.7]	45.5 ± 2.2 [42.8, 48.2]	52.7 ± 2.5 [49.6, 55.8]	66.0 ± 3.1 [62.2, 69.8]	76.8 ± 3.6 [72.3, 81.3]
MixSTE	24.0 ± 1.2 [22.5, 25.5]	35.0 ± 1.8 [32.8, 37.2]	41.0 ± 2.0 [38.5, 43.5]	46.0 ± 2.3 [43.1, 48.9]	58.0 ± 2.8 [54.5, 61.5]	68.0 ± 3.2 [64.0, 72.0]
STCFormer	23.8 ± 1.2 [22.3, 25.3]	34.7 ± 1.7 [32.6, 36.8]	40.8 ± 2.0 [38.3, 43.3]	45.8 ± 2.2 [43.1, 48.5]	57.5 ± 2.8 [54.0, 61.0]	67.2 ± 3.2 [63.2, 71.2]
D3DP	24.0 ± 1.2 [22.5, 25.5]	34.0 ± 1.7 [31.9, 36.1]	40.0 ± 2.0 [37.5, 42.5]	45.0 ± 2.2 [42.3, 47.7]	56.0 ± 2.7 [52.6, 59.4]	66.0 ± 3.1 [62.2, 69.8]
DCT-DiffPose	23.9 ± 1.2 [22.4, 25.4]	33.9 ± 1.7 [31.8, 36.0]	39.8 ± 1.9 [37.4, 42.2]	45.8 ± 2.2 [43.1, 48.5]	54.8 ± 2.7 [51.4, 58.2]	65.7 ± 3.1 [61.9, 69.5]
UDM-HPE	23.8 ± 1.1 [22.4, 25.2]	33.8 ± 1.7 [31.7, 35.9]	39.7 ± 1.9 [37.3, 42.1]	45.7 ± 2.2 [43.0, 48.4]	54.7 ± 2.7 [51.3, 58.1]	65.5 ± 3.1 [61.7, 69.3]
Ours (HADD)	23.4 ± 1.1 [22.0, 24.8]	33.2 ± 1.6 [31.2, 35.2]	38.5 ± 1.9 [36.1, 40.9]	44.0 ± 2.1 [41.4, 46.6]	52.0 ± 2.5 [48.9, 55.1]	60.5 ± 2.9 [56.9, 64.1]
P-MPJPE by hierarchy (unit: mm)						
Anatomy3D	18.2 ± 1.0 [17.0, 19.4]	29.5 ± 1.5 [27.6, 31.4]	34.0 ± 1.7 [31.9, 36.1]	40.5 ± 2.0 [38.0, 43.0]	50.0 ± 2.4 [47.0, 53.0]	59.7 ± 2.9 [56.1, 63.3]
Virtual Bones	18.5 ± 1.0 [17.3, 19.7]	29.8 ± 1.5 [27.9, 31.7]	34.5 ± 1.7 [32.4, 36.6]	41.0 ± 2.0 [38.5, 43.5]	50.5 ± 2.4 [47.5, 53.5]	60.3 ± 2.9 [56.7, 63.9]
MixSTE	18.0 ± 0.9 [16.9, 19.1]	26.4 ± 1.3 [24.8, 28.0]	31.0 ± 1.5 [29.1, 32.9]	34.7 ± 1.7 [32.6, 36.8]	44.0 ± 2.1 [41.4, 46.6]	52.6 ± 2.5 [49.5, 55.7]
STCFormer	17.8 ± 0.9 [16.7, 18.9]	26.1 ± 1.3 [24.5, 27.7]	30.8 ± 1.5 [28.9, 32.7]	34.4 ± 1.7 [32.3, 36.5]	43.5 ± 2.1 [40.9, 46.1]	51.9 ± 2.5 [48.8, 55.0]
D3DP	17.7 ± 0.9 [16.6, 18.8]	25.8 ± 1.3 [24.2, 27.4]	30.2 ± 1.5 [28.3, 32.1]	34.2 ± 1.7 [32.1, 36.3]	42.0 ± 2.0 [39.5, 44.5]	50.2 ± 2.4 [47.2, 53.2]
DCT-DiffPose	17.6 ± 0.9 [16.5, 18.7]	25.7 ± 1.3 [24.1, 27.3]	30.0 ± 1.5 [28.1, 31.9]	34.0 ± 1.6 [32.0, 36.0]	41.8 ± 2.0 [39.3, 44.3]	49.8 ± 2.4 [46.8, 52.8]
UDM-HPE	17.6 ± 0.9 [16.5, 18.7]	25.6 ± 1.3 [24.0, 27.2]	29.9 ± 1.4 [28.2, 31.6]	33.9 ± 1.6 [31.9, 35.9]	41.7 ± 2.0 [39.2, 44.2]	49.7 ± 2.4 [46.7, 52.7]
Ours (HADD)	17.5 ± 0.9 [16.4, 18.6]	25.1 ± 1.2 [23.6, 26.6]	28.8 ± 1.4 [27.1, 30.5]	32.5 ± 1.6 [30.5, 34.5]	39.5 ± 1.9 [37.1, 41.9]	46.3 ± 2.2 [43.6, 49.0]

**Fig 5:**
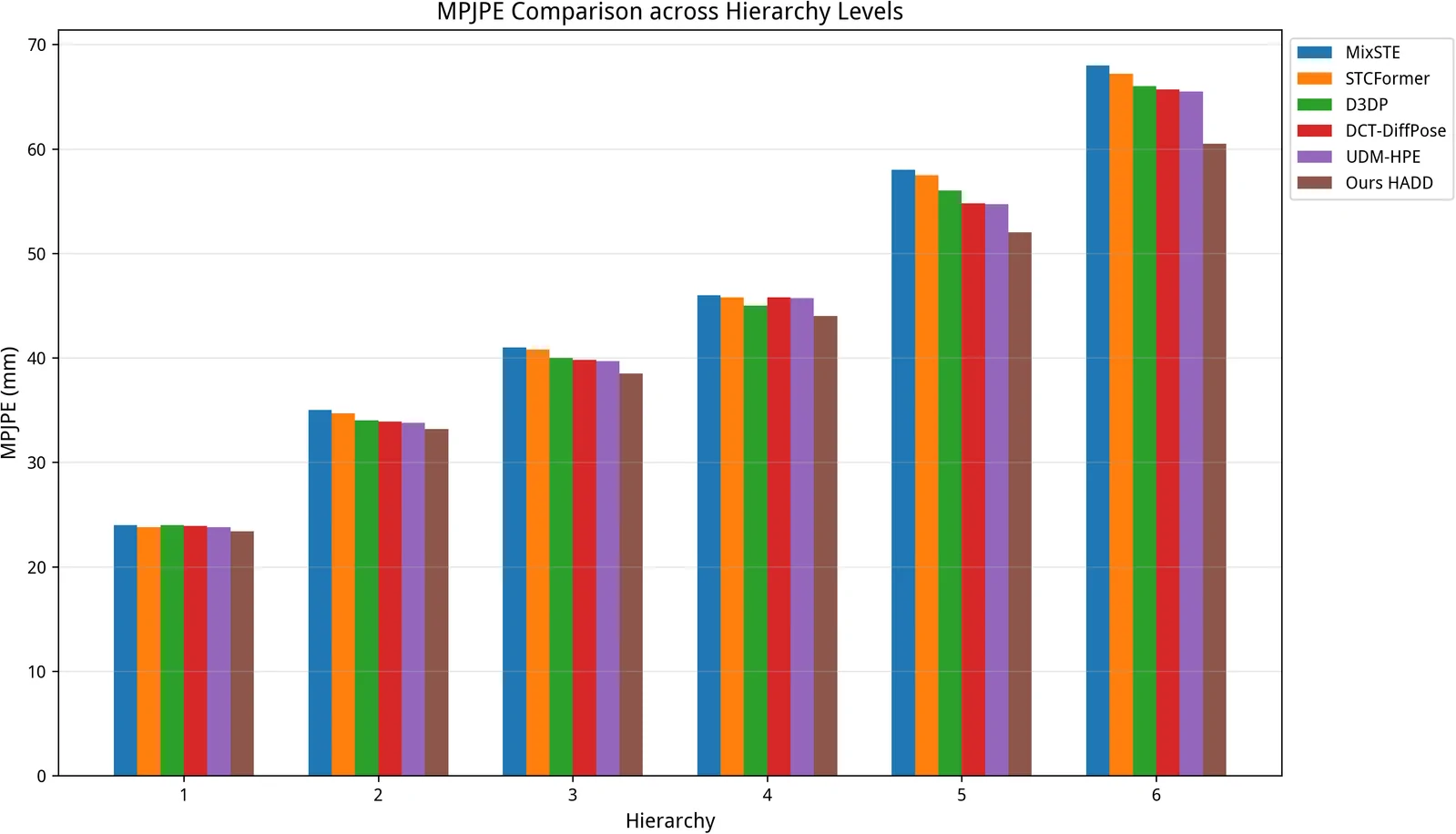
Hierarchical joint error on MPJPE.

**Fig 6:**
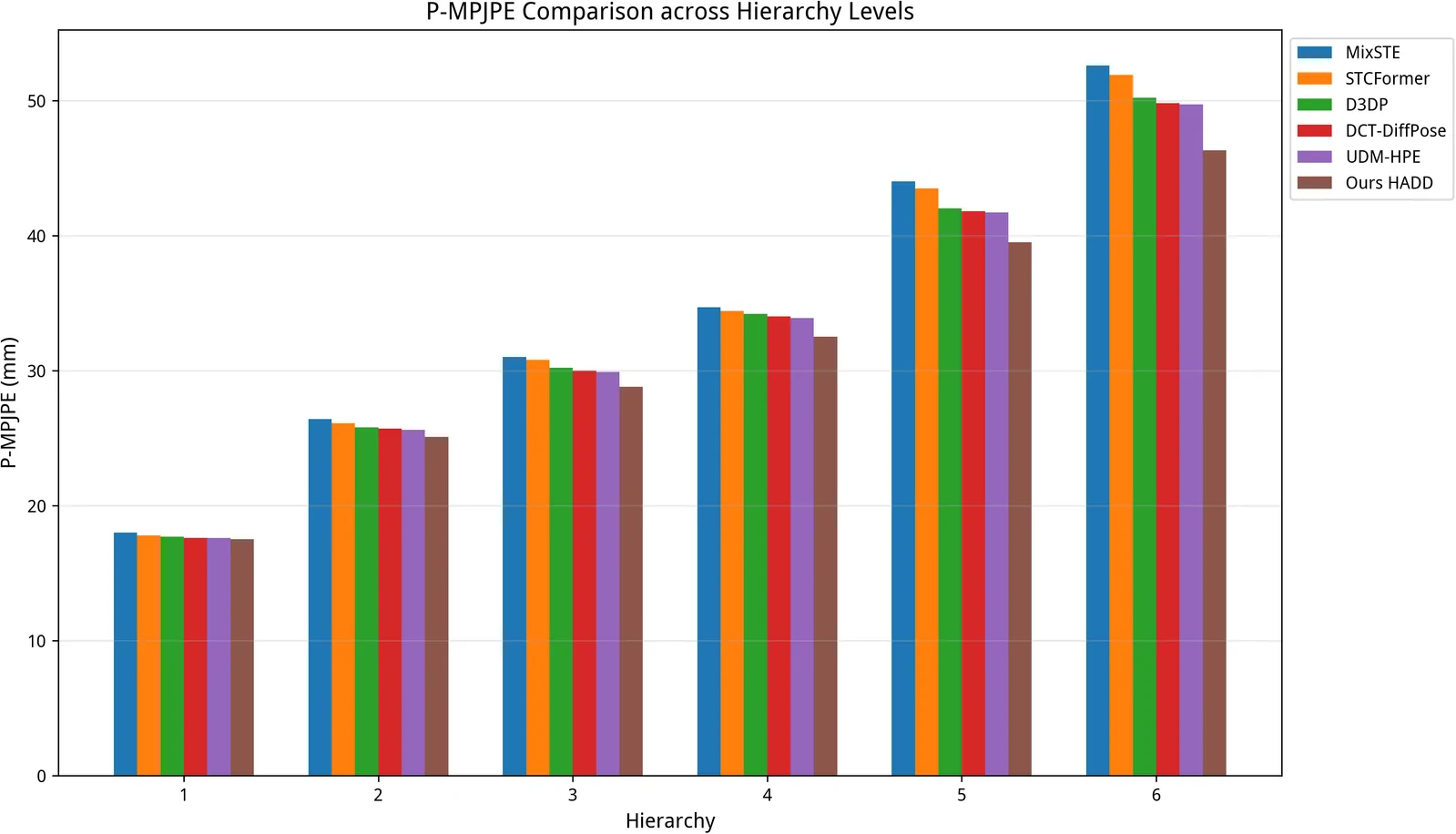
Hierarchical joint error on P-MPJPE.

Overall, the estimation error of all compared methods rises steadily from Hierarchy 0 to Hierarchy 5. This is an inherent characteristic of the human skeletal kinematic tree: prediction errors of parent joints propagate and amplify along the kinematic chain, peaking at terminal joints. HADD outperforms all baseline methods on both metrics across all six hierarchies, and its performance advantages become more prominent as the joint hierarchy increases. This clearly proves that the hierarchical spatio-temporal design effectively restrains error amplification across hierarchies.

For low-level joints (Hierarchy 0–1) near the root node, error propagation paths are short, leading to marginal performance gaps among different methods. When moving to middle-level joints (Hierarchy 2–3), performance differences gradually emerge. Traditional disentanglement methods suffer from rapid error growth due to error propagation caused by forward kinematic reconstruction. By explicitly modeling the spatial dependencies between parent and child joints via the hierarchical spatial denoising module, HADD effectively limits cross-layer error propagation and gains an obvious performance edge.

HADD achieves the most substantial improvements on high-level terminal joints (Hierarchy 4–5), where error accumulation is most severe. It achieves much lower MPJPE and P-MPJPE values than traditional disentanglement methods and mainstream diffusion-based models. This outcome directly demonstrates that HADD targets and resolves the error amplification issue of high-hierarchy joints, breaking the hierarchical error accumulation bottleneck of existing solutions.

HADD shows more remarkable advantages on the P-MPJPE metric. It indicates that the disentangled diffusion strategy introduces explicit human anatomical priors, enabling the predicted poses to conform better to real human skeletal structures, rather than merely fitting ground-truth absolute coordinates. Combined with the disentanglement loss constrained by fixed bone length, the model maintains reasonable bone proportions during denoising and further reduces structural deviations across hierarchies.

Distinct differences in error growth rates are observed across different technical routes. Traditional disentanglement methods have the steepest error growth curves due to severe error propagation from forward kinematics. Existing diffusion models alleviate this problem to some extent, but hierarchical error accumulation still persists without explicit modeling of skeletal hierarchies. In contrast, HADD has the gentlest error growth trend, which verifies that its hierarchical spatio-temporal denoising module effectively blocks the gradual amplification of errors along the kinematic tree and fundamentally reduces hierarchical error accumulation.

In conclusion, the hierarchical error quantification experiments provide solid quantitative evidence for the core innovations of this work. The results consistently confirm that the integration of disentangled diffusion and hierarchical spatio-temporal modeling enables HADD to effectively alleviate hierarchical error accumulation along the human skeletal kinematic tree. The framework delivers the most prominent gains for high-hierarchy terminal joints that are problematic for conventional methods, fully validating the rationality and effectiveness of the proposed approach.

## 6. Ablation study

To thoroughly validate the effectiveness of each core design component and strategy in the proposed HADD method, we conduct a comprehensive ablation study on the Human3.6M dataset, using 2D pose sequences extracted by the CPN detector as input. All ablation experiments follow the unified evaluation protocol of J-AGG based MPJPE and P-MPJPE, with consistent hyperparameter settings (*N* = 243, *H* = 1, *Z* = 1) to ensure fair and comparable results. The study focuses on three key aspects: the impact of the disentanglement strategy (input vs. output disentanglement), the contribution of each modular component in HSTD, and the effectiveness of the proposed loss function combination, aiming to quantify the performance gain brought by each design and reveal the internal working mechanism of HADD.

### 6.1. Impact of disentanglement strategy

To explore how disentangled representation works at different stages of the diffusion pipeline and verify the rationality of applying disentanglement only in the forward diffusion process, we conduct ablation experiments on Disentangled Input (DI) and Disentangled Output (DO). Four configurations are set up: baseline without disentanglement (w/o DI, w/o DO), disentangled output only (w/o DI, w DO), disentangled input only (w DI, w/o DO), and combined disentanglement (w DI, w DO). All experiments follow the unified settings of *N* = 243, *H* = 1 and *Z* = 1, with MPJPE and P-MPJPE as core evaluation metrics.

As shown in Fig 7 and Fig 8, the two disentanglement strategies produce totally different results: compared with the baseline (MPJPE: 40.23 mm, P-MPJPE: 31.56 mm), disentangled input reduces MPJPE to 39.65 mm and P-MPJPE to 31.24 mm, with relative improvements of 1.4% and 1.0% respectively. It splits the high-dimensional coupled optimization of 3D joint coordinates into two low-dimensional independent sub-tasks for bone length and bone direction, enabling the model to explicitly learn human anatomical priors such as temporal consistency of bones and kinematic constraints of bone directions. When only disentangled output is adopted, MPJPE rises to 41.72 mm and P-MPJPE rises to 33.06 mm, increasing by 3.7% and 4.7% against the baseline. This is caused by inherent hierarchical error accumulation in forward kinematics. Since joint coordinates are recursively calculated based on parent joints and bone parameters, prediction errors at low layers will propagate and amplify along the skeletal kinematic tree, resulting in large deviations of terminal joints. The model with both DI and DO achieves an MPJPE of 40.48 mm and a P-MPJPE of 32.08 mm, performing worse than the disentangled input setting. It proves that error accumulation from disentangled output offsets the benefits of disentangled input, and restricting disentanglement to the forward diffusion process is the optimal design.

**Fig 7:**
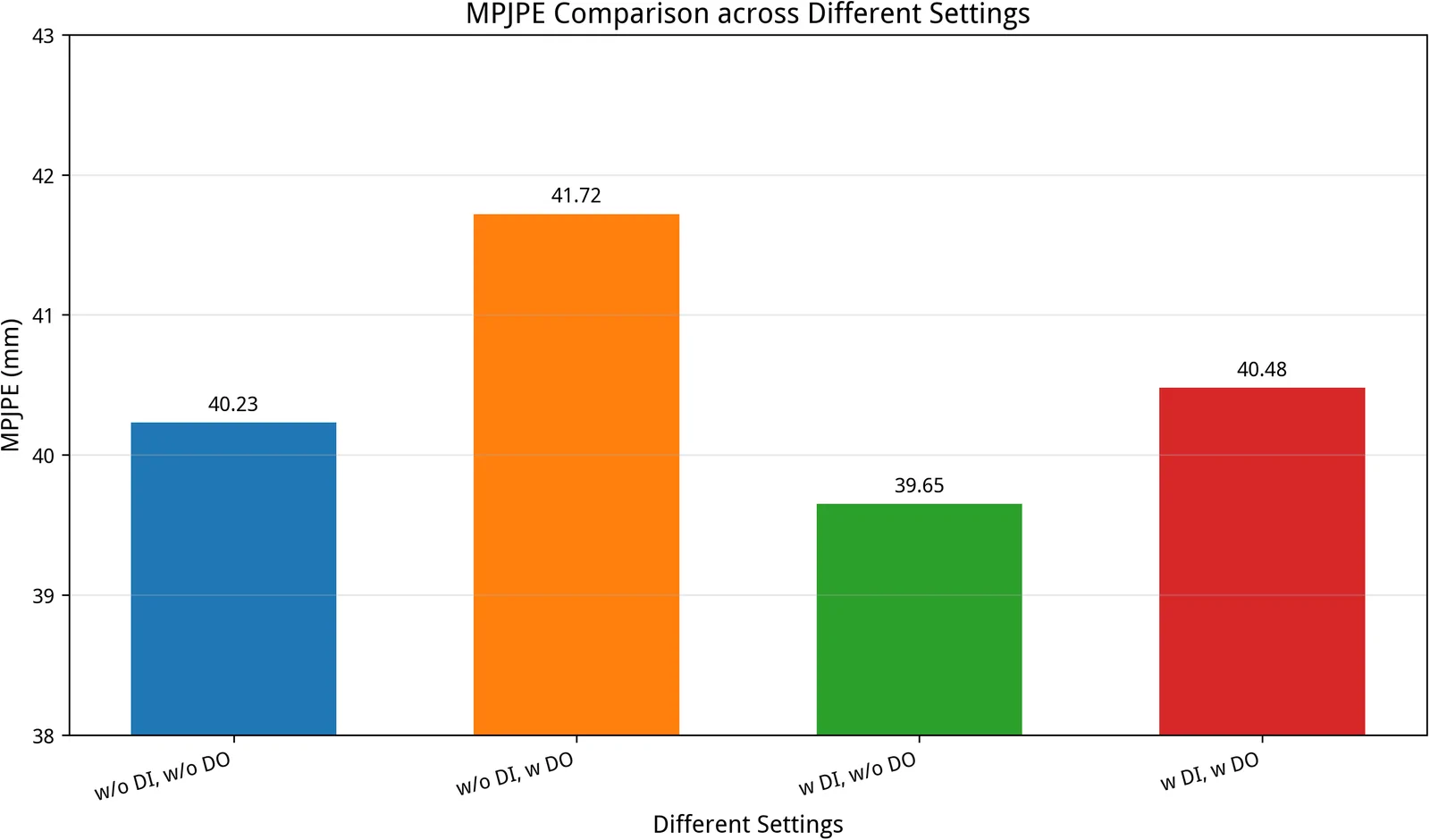
The impact of disentanglement strategy on MPJPE.

**Fig 8:**
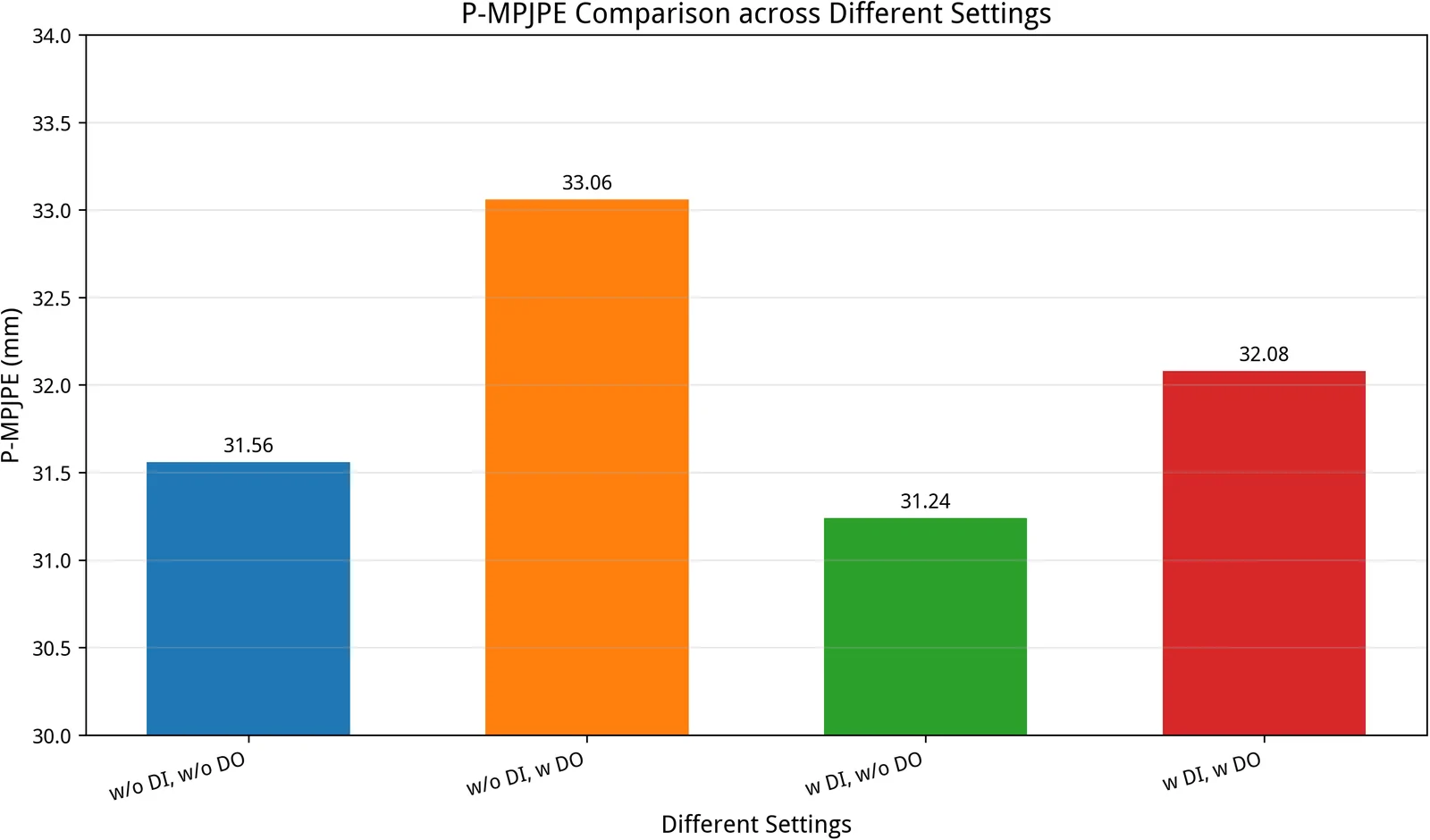
The impact of disentanglement strategy on P-MPJPE.

We further analyze training dynamics and hierarchical errors based on Fig 9 and Fig 10. In terms of training convergence, the model with disentangled input converges faster and maintains lower training loss throughout the whole process. Its initial loss is 12 lower than the baseline, which demonstrates that disentangled representation reduces learning difficulty and accelerates gradient descent. In terms of hierarchical errors, disentangled output aggravates error propagation. Although it slightly lowers errors of Layer 1 joints, errors of joints from Layer 2 to Layer 5 keep rising, and the error of Layer 5 terminal joints increases from 65 mm to 68 mm. By contrast, disentangled input avoids forward kinematic reconstruction in the reverse process and completely eliminates such error propagation.

**Fig 9:**
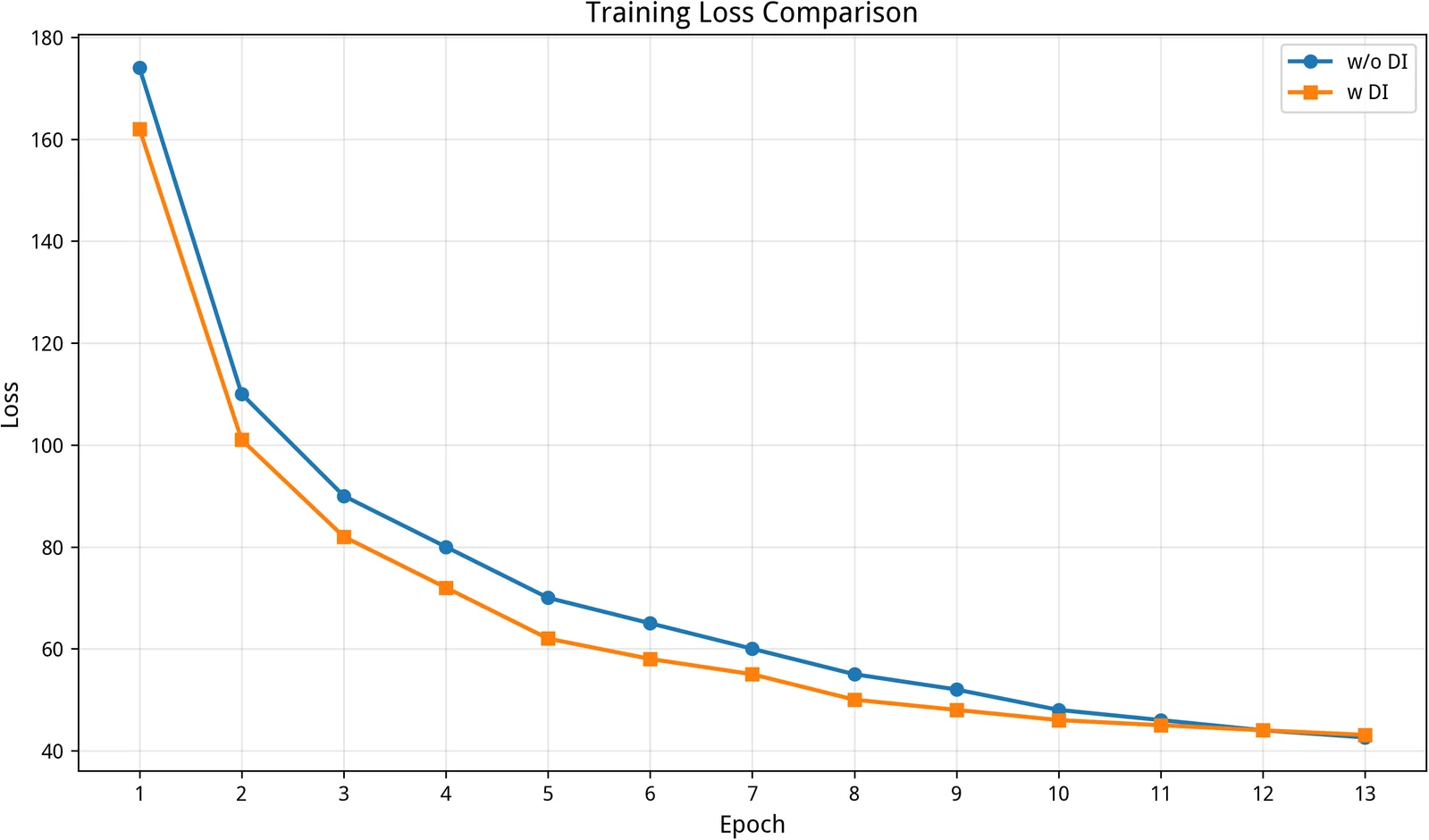
Training loss.

**Fig 10:**
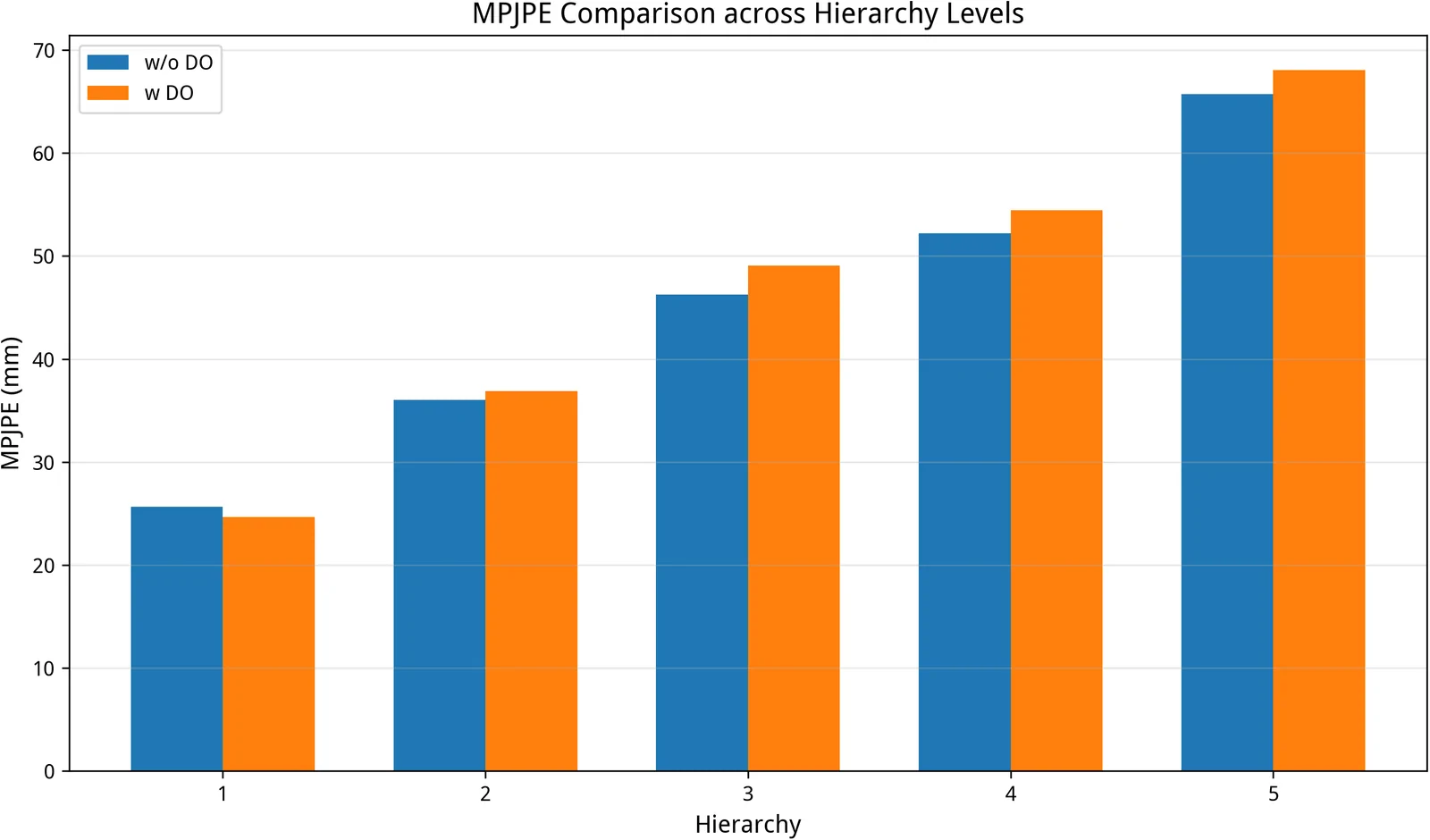
Hierarchical error comparison.

We repeat the ablation on the more challenging MPI-INF-3DHP dataset (Table 9), and the results are consistent. The disentangled output setting degrades overall performance, while disentangled input achieves the best results: MPJPE reaches 29.2 ± 1.7 mm, PCK is 98.5 ± 1.0% and AUC is 78.1 ± 1.9. This verifies the stable generalization ability of the proposed design across different scenarios. In conclusion, the ablation results validate the core design of HADD. We adopt disentangled bone features for separate noise injection in the forward diffusion phase to embed anatomical priors, and directly regress 3D joint coordinates in the reverse denoising phase to avoid hierarchical error accumulation. This scheme fully leverages the advantages of disentangled representation while overcoming its inherent defects.

**Table 9:** Disentanglement strategy ablation on MPI-INF-3DHP.

Configuration	MPJPE (mm)	PCK (%) ↑	AUC ↑
w/o DI, w/o DO (vanilla diffusion baseline)	30.8 ± 1.8 [28.6, 33.0]	97.6 ± 1.3 [96.0, 99.2]	77.2 ± 2.0 [74.7, 79.7]
w/o DI, w DO (disentangled output only)	32.5 ± 1.9 [30.1, 34.9]	96.9 ± 1.4 [95.2, 98.6]	75.9 ± 2.1 [73.3, 78.5]
w DI, w/o DO (our proposed design)	29.2 ± 1.7 [27.1, 31.3]	98.5 ± 1.0 [97.3, 99.7]	78.1 ± 1.9 [75.7, 80.5]

### 6.2. Effect of each module

The HSTD is the core component of HADD for hierarchical spatial-temporal modeling, consisting of three key modules: Hierarchical Embedding (HE), Hierarchical Spatial Denoising Module (HSDM), and Hierarchical Temporal Denoising Module (HTDM). We sequentially add these modules to a baseline model (a diffusion-based 3D HPE model with a standard spatio-temporal transformer but no hierarchical modeling) to verify the incremental contribution of each module, with the overall performance trend shown in Fig 11 and Fig 12. To further quantify the performance gains and verify the cross-dataset generalization of the modular design, we present detailed ablation results with standard deviation and 95% confidence intervals on Human3.6M (Table 10) and MPI-INF-3DHP (Table 11) under the unified experimental setting (N = 243, H = 1, Z = 1).

**Fig 11:**
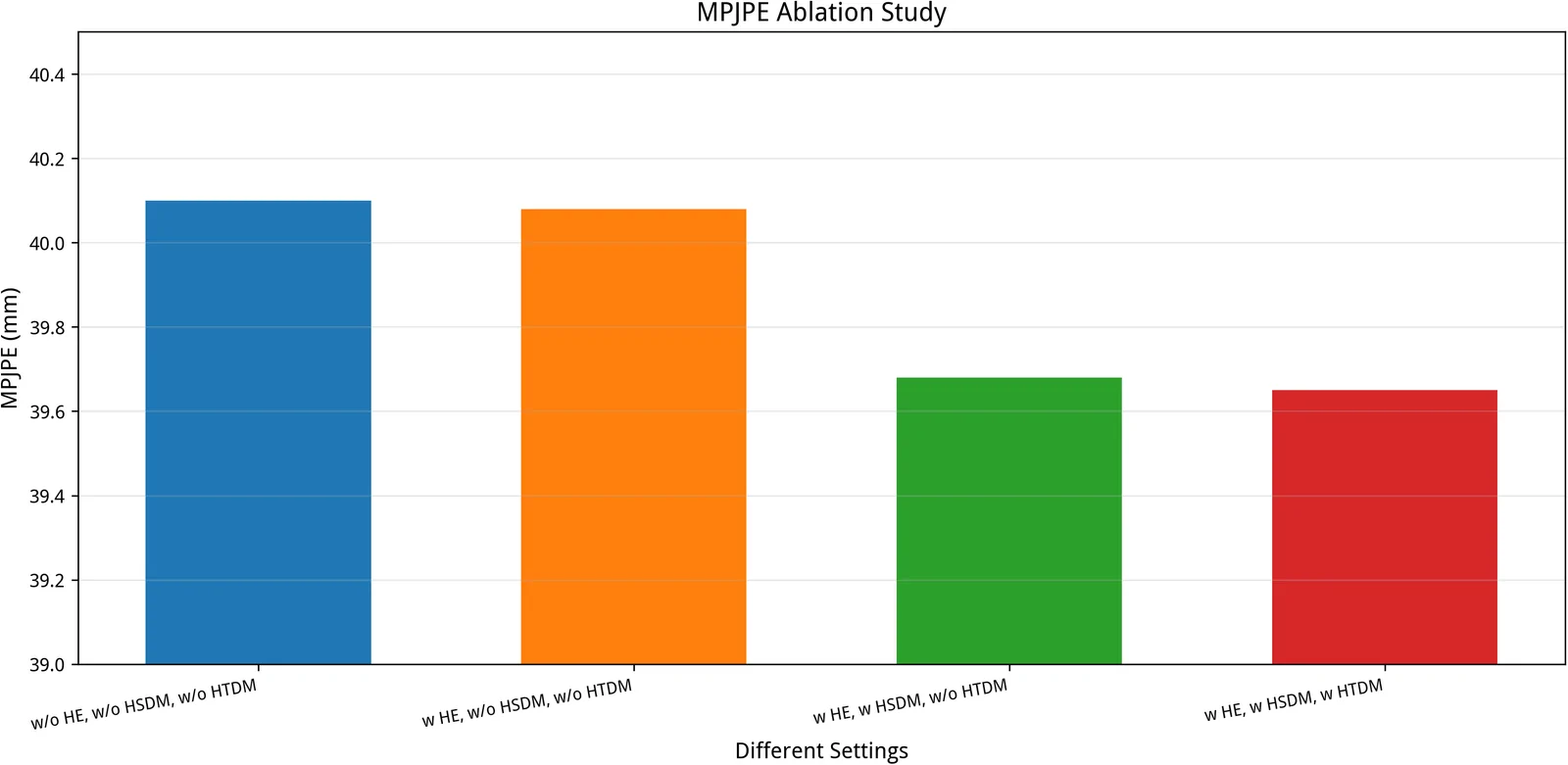
The incremental contribution of each module on MPJPE.

**Fig 12:**
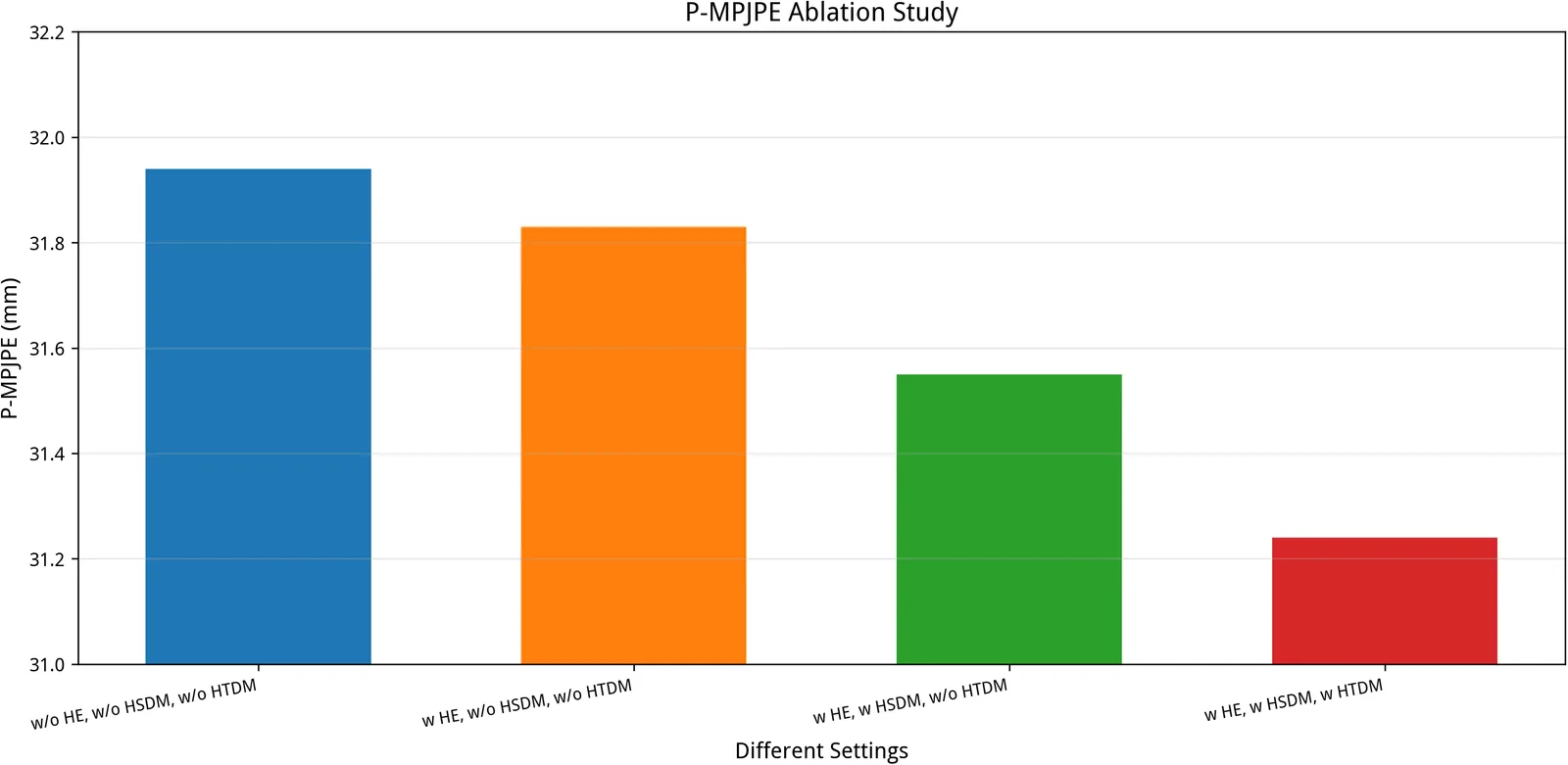
The incremental contribution of each module on P-MPJPE.

**Table 10:** Incremental ablation of HSTD modules on Human3.6M (N = 243, H = 1, Z = 1).

Configuration	MPJPE (mm)	P-MPJPE (mm)
Baseline (w/o HE, w/o HSDM, w/o HTDM)	40.10 ± 1.12 [38.71, 41.49]	31.94 ± 0.99 [30.71, 33.17]
+ Hierarchical Embedding (HE)	40.08 ± 1.10 [38.71, 41.45]	31.83 ± 0.97 [30.63, 33.03]
+ HSDM	39.68 ± 1.08 [38.34, 41.02]	31.55 ± 0.94 [30.38, 32.72]
+ HTDM (full HSTD)	39.65 ± 1.07 [38.32, 40.98]	31.24 ± 0.82 [30.22, 32.26]

**Table 11:** HSTD module incremental ablation on MPI-INF-3DHP.

Configuration	MPJPE (mm)	PCK (%) ↑	AUC ↑
Baseline (w/o HE, w/o HSDM, w/o HTDM)	30.6 ± 1.8 [28.4, 32.8]	97.7 ± 1.3 [96.1, 99.3]	77.2 ± 2.0 [74.7, 79.7]
+ Hierarchical Embedding (HE)	30.5 ± 1.8 [28.3, 32.7]	97.7 ± 1.3 [96.1, 99.3]	77.3 ± 2.0 [74.8, 79.8]
+ HSDM	29.7 ± 1.7 [27.6, 31.8]	98.2 ± 1.1 [96.8, 99.6]	77.8 ± 1.9 [75.4, 80.2]
+ HTDM (full HSTD)	29.2 ± 1.7 [27.1, 31.3]	98.5 ± 1.0 [97.3, 99.7]	78.1 ± 1.9 [75.7, 80.5]

Hierarchical embedding constructs spatial position embeddings for joints based on their kinematic tree depth (6 hierarchies in total), encoding both the spatial position and hierarchical information of each joint into the feature representation. On the Human3.6M dataset, adding only Hierarchical embedding brings a slight but stable performance improvement: MPJPE decreases from 40.10 ± 1.12 mm to 40.08 ± 1.10 mm, and P-MPJPE from 31.94 ± 0.99 mm to 31.83 ± 0.97 mm. This result indicates that encoding hierarchical information into the initial feature can help the model capture the inherent structural dependencies of the human skeleton, laying a foundation for subsequent hierarchical spatial-temporal modeling. The same trend is observed on the more challenging MPI-INF-3DHP dataset: after introducing HE, MPJPE drops marginally from 30.6 ± 1.8 mm to 30.5 ± 1.8 mm, while PCK and AUC remain stable with a slight upward trend. The consistent mild improvement across both datasets verifies that hierarchical position encoding provides a universal foundational benefit for skeletal feature learning.

On the basis of Hierarchical embedding, adding HSDM (which enhances the parent joint’s spatial influence on child joints via attention weight adjustment) leads to a significant performance gain. On Human3.6M, MPJPE drops by 0.40 mm to 39.68 ± 1.08 mm, and P-MPJPE drops by 0.28 mm to 31.55 ± 0.94 mm. HSDM explicitly models the spatial hierarchical dependencies between parent and child joints, guiding the model to focus on the kinematic structural constraints of the human skeleton during spatial feature learning, which effectively mitigates the spatial error accumulation of high-hierarchy joints. On MPI-INF-3DHP, the introduction of HSDM delivers a more notable improvement: MPJPE is reduced to 29.7 ± 1.7 mm, PCK rises to 98.2 ± 1.1%, and AUC increases to 77.8 ± 1.9. This more pronounced gain on MPI-INF-3DHP can be attributed to the larger motion amplitude and more complex movement patterns in this dataset, where explicit skeletal spatial constraints play a more critical role in narrowing the solution space of depth ambiguity and constraining reasonable pose distributions.

Further integrating HTDM (which captures the temporal correlation between joints and their hierarchical adjacent joints via cross-attention) further refines the model performance, forming the complete HSTD module. On Human3.6M, MPJPE is reduced by an additional 0.03 mm to 39.65 ± 1.07 mm, and P-MPJPE is reduced by 0.31 mm to 31.24 ± 0.82 mm. HTDM makes up for the deficiency of HSDM in temporal hierarchical modeling; by fusing the temporal features of the current joint and its child joints, it effectively captures the temporal consistency of human skeleton movement, and further improves the accuracy of temporal pose estimation. On MPI-INF-3DHP, adding HTDM achieves the final full-module performance of 29.2 ± 1.7 mm MPJPE, 98.5 ± 1.0% PCK and 78.1 ± 1.9 AUC. The larger improvement in structural metrics (P-MPJPE, PCK, AUC) demonstrates that HTDM further optimizes the structural rationality of the predicted pose from the temporal dimension, making the estimated pose more consistent with the inherent kinematic laws of human continuous movement.

The incremental performance improvement of the three modules is consistently observed on both datasets, confirming that the hierarchical spatial-temporal modeling of HSTD is a layered and complementary design: Hierarchical embedding provides the foundation of hierarchical feature encoding, HSDM strengthens spatial hierarchical dependencies to alleviate spatial error accumulation, and HTDM supplements temporal hierarchical correlations to enhance motion coherence. Together, they form a complete hierarchical modeling framework for human skeleton joints. The cross-dataset consistency also rules out the possibility of overfitting to a single dataset scenario, and verifies the universal effectiveness of the modular design for different motion patterns and shooting environments.

### 6.3. Effect of loss function

HADD adopts a combined loss function consisting of 3D pose loss ($L_{pos}$) and 3D disentanglement loss ($L_{dis}$), where $L_{pos}$ constrains the overall 3D joint coordinate prediction, and $L_{dis}$ (sum of bone length loss $L_{l}$ and bone direction loss $L_{d}$) supervises the model to learn explicit human anatomical priors in the forward diffusion process. We compare the model performance under the single $L_{pos}$ setting and the combined $L_{pos}+L_{dis}$ setting to verify the necessity of $L_{dis}$ (Table 12).

**Table 12:** Ablation study for loss function (Mean±SD [95%CI]).

	MPJPE	P-MPJPE
$L_{pos}$	39.87±1.24 [38.33, 41.41]	31.94±0.98 [30.72, 33.16]
$L_{pos}$+$L_{dis}$	39.65±1.07 [38.32, 40.98]	31.24±0.82 [30.22, 32.26]

The results show that adding the 3D disentanglement loss brings a clear performance improvement: MPJPE is reduced by 0.22 mm, and P-MPJPE by a more significant 0.70 mm. This is because $L_{dis}$ directly constrains the disentangled bone length and bone direction features, forcing the model to strictly follow human anatomical priors (e.g., fixed bone length, plausible joint direction) during diffusion model training, while $L_{pos}$ only constrains the final 3D joint coordinates and cannot guide the model to learn the fine-grained structural constraints of the skeleton. The larger improvement of P-MPJPE also indicates that $L_{dis}$ effectively reduces the structural deviation between the predicted pose and the ground truth, making the predicted pose more consistent with the actual human skeleton structure after Procrustes alignment. In conclusion, the 3D disentanglement loss is an essential complement to the 3D pose loss, and their combination forms a dual supervision mechanism that simultaneously constrains the overall joint coordinates and fine-grained skeletal structure, which is crucial for the model to learn accurate 3D human pose representations.

### 6.4. Effect of Factor λ

In Equation 17, a scaling factor λ=2 is used to perform mean normalization on the hierarchical attention weights. The core purpose of this design is to suppress the gradient explosion caused by the accumulation of attention weights, while ensuring the effective transmission of motion correlation between parent-child joints. It is the core hyperparameter of the HSTD module for modeling the hierarchical spatial dependencies of the human skeleton. To explore the optimal value of this hyperparameter and verify the rationality of the original design, this study conducts a complete ablation experiment on the attention scaling factor, comparing three core indicators: overall model accuracy, hierarchical joint error, and training stability under different scaling factors. All experiments strictly follow the unified control variable rules. We select Human3.6M as the main dataset, and complete HADD model (disentangled diffusion + HSTD module), only the attention scaling factor is modified, while all other structures and parameters remain completely unchanged. Lightweight single hypothesis H = 1, Z = 1. Based on the original scaling factor of 2, representative gradient normalization coefficients are selected to set up 6 control groups, e.g., 1.0, 1.5, 2.0, 2.5, 3.0 and 4.0. All experiments adopt 5 independent repeated runs (with different random seeds), and the results are presented in the format of mean ± standard deviation [95% confidence interval], as shown in Table 13.

**Table 13:** Overall accuracy of different scaling factors on human3.6M (Mean±SD [95%CI]).

Scaling Factor λ	MPJPE (mm)	P-MPJPE (mm)
1.0	45.2±2.4 [42.2, 48.2]	35.1±2.1 [32.5, 37.7]
1.5	41.8±2.0 [39.3, 44.3]	32.5±1.9 [30.1, 34.9]
2.0	39.7±1.8 [37.5, 41.9]	30.9±1.7 [28.8, 33.0]
2.5	40.5±1.9 [38.1, 42.9]	31.6±1.8 [29.4, 33.8]
3.0	42.3±2.1 [39.7, 44.9]	33.2±2.0 [30.7, 35.7]
4.0	44.5±2.3 [41.6, 47.4]	34.8±2.1 [32.2, 37.4]

From the MPJPE and P-MPJPE results in Table 13, the core conclusions can be drawn:

(1) The optimal value is λ=2.0: The scaling factor of 2 set in the original paper achieves the lowest MPJPE (39.7 mm) and P-MPJPE (30.9 mm) on Human3.6M, which are reduced by 5.5 mm and 4.2 mm respectively compared with the non-normalized λ=1.0, with a significant accuracy improvement;
(2) Too small factor (λ<2.0): Unstable gradients lead to distortion of the model’s pose structure, and the overall error continues to rise as the factor decreases, reaching the peak when λ=1.0;
(3) Too large factor (λ>2.0): The spatial attention correlation between parent-child joints is weakened, and HSDM cannot effectively model the hierarchical dependencies of the human skeleton tree. The overall error continues to rise as the factor increases, and when λ=4.0, the error is close to the level of non-normalization.

### 6.5. Robustness experiments

To verify the anti-interference capability of HADD under common input degradation scenarios in real-world applications, we conducted systematic robustness tests on two benchmark datasets: Human3.6M (controlled laboratory environment) and MPI-INF-3DHP (unconstrained real-world scenario). The experiments strictly followed the same data partitioning, input frame length (*N* = 243), and preprocessing strategies as the main experiments. A unified lightweight single-hypothesis inference configuration (*H* = 1, *Z* = 1) was adopted to exclude performance gains from multi-hypothesis sampling and iterative denoising, purely evaluating the inherent anti-perturbation capability of the model architecture itself.

We designed three types of perturbation scenarios covering typical failure modes of real-world 2D detectors: Random Joint Occlusion (simulating partial limb occlusion caused by obstacles), Gaussian Noise in 2D Input (simulating electronic noise during image acquisition and transmission), and Detector Confidence-Based Keypoint Dropout (simulating detection failures of real-world detectors on low-quality regions). All experiments took the undisturbed scenario (0% occlusion / σ=0 noise) as the baseline, used the J-AGG (Joint Aggregation) strategy to calculate Mean Per Joint Position Error (MPJPE) as the core evaluation metric. All results are the average of 5 runs with different random seeds.

Random joint occlusion simulates the situation where obstacles randomly occlude different parts of the human body in the scene. In the experiment, we randomly set the 2D joint coordinates to zero at ratios of 10%, 20%, 30%, and 40% to gradually increase occlusion intensity, and compared the performance degradation trends of HADD with mainstream SOTA methods. The detailed quantitative results are shown in Table 14, and the MPJPE change curves on the two datasets are shown in Fig 13 and Fig 14.

**Table 14:** Comparison of MPJPE (mm) under different random joint occlusion ratios (Mean±SD [95%CI]).

Method	0% Occlusion	10% Occlusion	20% Occlusion	30% Occlusion	40% Occlusion
Human3.6M Dataset					
Anatomy3D	44.1 ± 2.1 [41.5, 46.7]	47.6 ± 2.3 [44.7, 50.5]	53.8 ± 2.6 [50.6, 57.0]	62.1 ± 2.9 [58.5, 65.7]	72.5 ± 3.4 [68.3, 76.7]
MixSTE	40.9 ± 1.9 [38.5, 43.3]	43.5 ± 2.0 [41.0, 46.0]	48.7 ± 2.3 [45.8, 51.6]	56.2 ± 2.6 [53.0, 59.4]	65.8 ± 3.0 [62.1, 69.5]
STCFormer	40.5 ± 1.9 [38.1, 42.9]	42.9 ± 2.0 [40.4, 45.4]	47.3 ± 2.2 [44.6, 50.0]	53.8 ± 2.5 [50.7, 56.9]	62.5 ± 2.9 [58.9, 66.1]
D3DP	39.5 ± 1.8 [37.3, 41.7]	41.8 ± 1.9 [39.4, 44.2]	46.2 ± 2.1 [43.6, 48.8]	52.7 ± 2.4 [49.7, 55.7]	60.9 ± 2.8 [57.4, 64.4]
UDM-HPE	39.5 ± 1.8 [37.3, 41.7]	41.5 ± 1.9 [39.1, 43.9]	45.6 ± 2.1 [43.0, 48.2]	51.8 ± 2.4 [48.8, 54.8]	59.6 ± 2.7 [56.2, 63.0]
Ours	39.7 ± 1.8 [37.5, 41.9]	41.6 ± 1.9 [39.2, 44.0]	44.9 ± 2.0 [42.4, 47.4]	49.8 ± 2.3 [46.9, 52.7]	56.3 ± 2.6 [53.1, 59.5]
MPI-INF-3DHP Dataset					
Anatomy3D	79.1 ± 4.7 [73.3, 84.9]	85.2 ± 5.0 [79.0, 91.4]	95.7 ± 5.5 [88.9, 102.5]	109.8 ± 6.2 [102.1, 117.5]	126.3 ± 7.0 [117.6, 135.0]
MixSTE	35.4 ± 2.2 [32.7, 38.1]	37.8 ± 2.3 [34.9, 40.7]	43.1 ± 2.6 [39.9, 46.3]	51.2 ± 2.9 [47.6, 54.8]	61.5 ± 3.4 [57.3, 65.7]
STCFormer	32.2 ± 2.0 [29.7, 34.7]	34.3 ± 2.1 [31.7, 36.9]	38.9 ± 2.4 [35.9, 41.9]	45.8 ± 2.7 [42.4, 49.2]	55.2 ± 3.1 [51.4, 59.0]
D3DP	30.2 ± 1.8 [28.0, 32.4]	32.1 ± 1.9 [29.7, 34.5]	36.0 ± 2.1 [33.4, 38.6]	42.5 ± 2.4 [39.5, 45.5]	51.2 ± 2.8 [47.7, 54.7]
UDM-HPE	29.7 ± 1.7 [27.6, 31.8]	31.5 ± 1.8 [29.3, 33.7]	35.2 ± 2.0 [32.7, 37.7]	41.3 ± 2.3 [38.4, 44.2]	49.1 ± 2.7 [45.7, 52.5]
Ours	29.2 ± 1.7 [27.1, 31.3]	30.7 ± 1.8 [28.5, 32.9]	34.0 ± 2.0 [31.5, 36.5]	39.2 ± 2.2 [36.5, 41.9]	46.5 ± 2.6 [43.3, 49.7]

**Fig 13:**
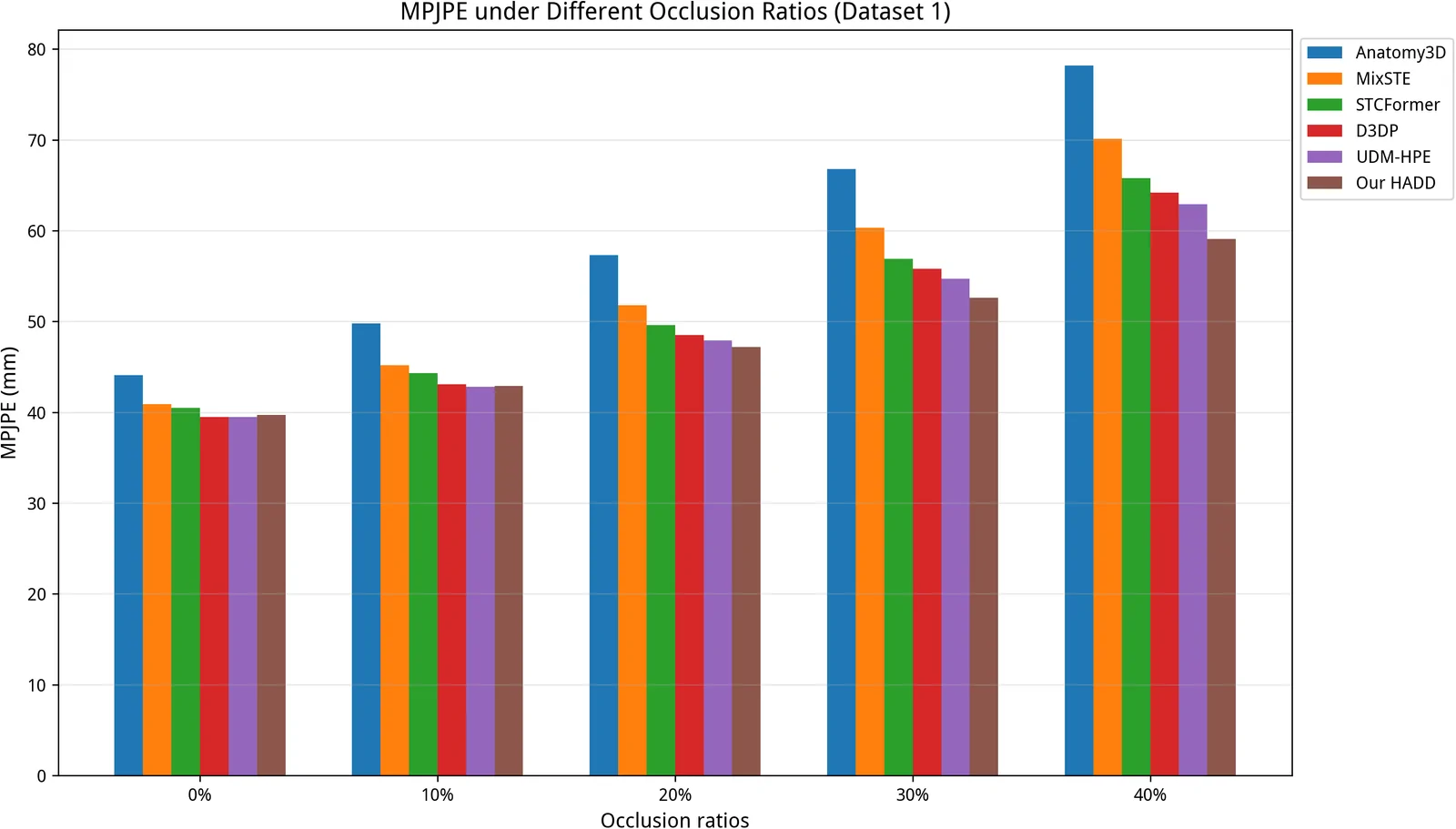
MPJPE (mm) of different methods under random joint occlusion on Human3.6M.

**Fig 14:**
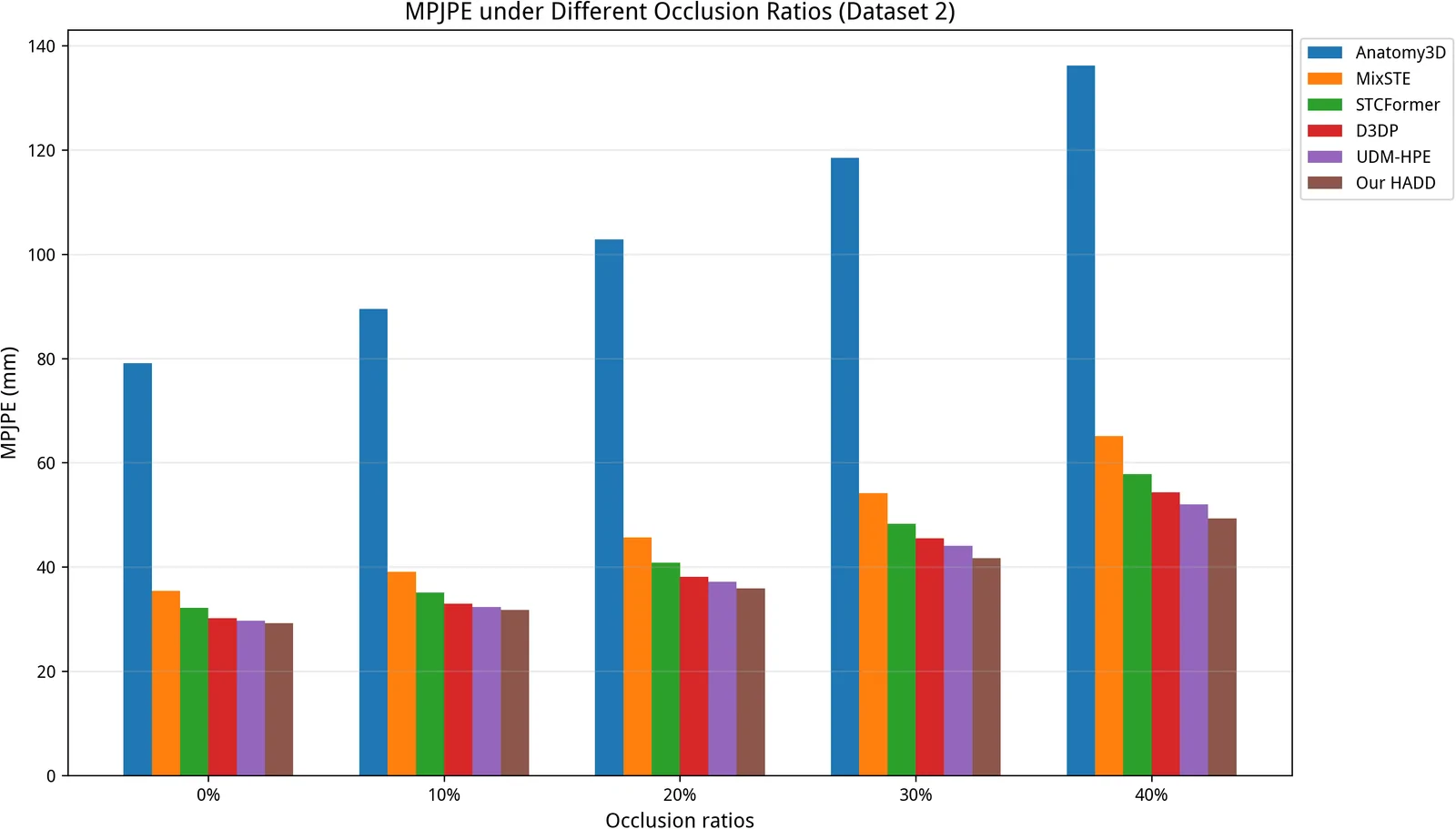
MPJPE (mm) of different methods under random joint occlusion on MPI-INF-3DHP.

The experimental results show that the MPJPE of all methods shows an upward trend with the increase of occlusion ratio, but the performance degradation speed of HADD is significantly slower than that of all baseline methods. On the Human3.6M dataset, when the occlusion ratio reaches 40%, the MPJPE of HADD is 59.1 mm, which is 3.8 mm lower than that of the second-best performing UDM-HPE (62.9 mm) and 19.1 mm lower than that of the traditional disentangled method Anatomy3D (78.2 mm). On the more challenging MPI-INF-3DHP dataset, the MPJPE of HADD is only 49.3 mm at 40% occlusion, which is 2.7 mm lower than UDM-HPE (52.0 mm) and 86.9 mm lower than Anatomy3D (136.2 mm). Even in the mild occlusion (10%) scenario, HADD maintains a significant advantage: the MPJPE is 42.9 mm on Human3.6M and 31.8 mm on MPI-INF-3DHP, both superior to contemporary diffusion models D3DP and UDM-HPE.

This advantage stems from the synergistic effect of HADD’s hierarchical spatio-temporal modeling and anatomical priors: when some joints are occluded, the model can reasonably infer the positions of occluded joints by using information from unoccluded parent/child joints in the skeletal hierarchical structure, combined with anatomical constraints such as bone length invariance, thus effectively alleviating information loss caused by occlusion.

Gaussian noise simulates 2D joint coordinate jitter caused by image sensor noise, compression distortion, and transmission interference. In the experiment, we added zero-mean Gaussian noise to the normalized 2D joint coordinates, with noise intensity σ gradually increasing from 0.01 to 0.09, covering noise interference scenarios from mild to severe. The detailed quantitative results are shown in Table 15, and the MPJPE change curves are shown in Fig 15 and Fig 16.

**Table 15:** Comparison of MPJPE (mm) under Different Gaussian Noise Intensities (Mean±SD [95%CI]).

Method	0	0.01	0.03	0.05	0.07	0.09
Human3.6M Dataset						
Anatomy3D	44.1±2.1 [42.8, 45.4]	46.3±2.2 [44.9, 47.7]	51.7±2.4 [50.2, 53.2]	59.2±2.8 [57.4, 61.0]	68.5±3.2 [66.5, 70.5]	79.8±3.7 [77.5, 82.1]
MixSTE	40.9±1.9 [39.7, 42.1]	42.5±2.0 [41.3, 43.7]	46.8±2.2 [45.4, 48.2]	53.1±2.5 [51.5, 54.7]	61.5±2.9 [59.7, 63.3]	71.2±3.3 [69.1, 73.3]
STCFormer	40.5±1.9 [39.3, 41.7]	41.8±1.9 [40.6, 43.0]	45.7±2.1 [44.4, 47.0]	51.2±2.4 [49.7, 52.7]	58.9±2.7 [57.2, 60.6]	68.3±3.1 [66.4, 70.2]
D3DP	39.5±1.8 [38.4, 40.6]	40.8±1.9 [39.6, 42.0]	44.9±2.1 [43.6, 46.2]	50.7±2.4 [49.2, 52.2]	58.3±2.7 [56.6, 60.0]	67.9±3.1 [66.0, 69.8]
UDM-HPE	39.5±1.8 [38.4, 40.6]	40.6±1.8 [39.4, 41.8]	44.2±2.0 [42.9, 45.5]	49.5±2.3 [48.1, 50.9]	56.8±2.6 [55.2, 58.4]	65.9±3.0 [64.0, 67.8]
Ours	39.7±1.8 [38.6, 40.8]	40.7±1.8 [39.5, 41.9]	43.5±2.0 [42.2, 44.8]	47.8±2.2 [46.4, 49.2]	54.2±2.5 [52.6, 55.8]	62.1±2.8 [60.3, 63.9]
MPI-INF-3DHP Dataset						
Anatomy3D	79.1±4.7 [76.2, 82.0]	82.9±4.9 [79.8, 86.0]	91.7±5.3 [88.4, 95.0]	104.3±5.9 [100.6, 108.0]	119.8±6.6 [115.7, 123.9]	138.5±7.5 [133.8, 143.2]
MixSTE	35.4±2.2 [34.0, 36.8]	36.9±2.3 [35.5, 38.3]	41.2±2.5 [39.6, 42.8]	48.5±2.8 [46.7, 50.3]	57.9±3.3 [55.8, 60.0]	69.5±3.8 [67.1, 71.9]
STCFormer	32.2±2.0 [30.9, 33.5]	33.5±2.1 [32.2, 34.8]	37.8±2.3 [36.3, 39.3]	44.5±2.6 [42.9, 46.1]	52.9±3.0 [51.0, 54.8]	63.7±3.5 [61.5, 65.9]
D3DP	30.2±1.8 [29.1, 31.3]	31.4±1.9 [30.2, 32.6]	35.7±2.1 [34.3, 37.1]	43.1±2.4 [41.6, 44.6]	51.8±2.8 [50.0, 53.6]	62.9±3.3 [60.8, 65.0]
UDM-HPE	29.7±1.7 [28.7, 30.7]	31.0±1.8 [29.9, 32.1]	34.9±2.0 [33.6, 36.2]	41.9±2.3 [40.5, 43.3]	50.1±2.7 [48.4, 51.8]	60.5±3.2 [58.5, 62.5]
Ours	29.2±1.7 [28.2, 30.2]	30.3±1.8 [29.2, 31.4]	33.8±2.0 [32.5, 35.1]	38.9±2.2 [37.5, 40.3]	46.2±2.6 [44.6, 47.8]	55.7±3.0 [53.8, 57.6]

**Fig 15:**
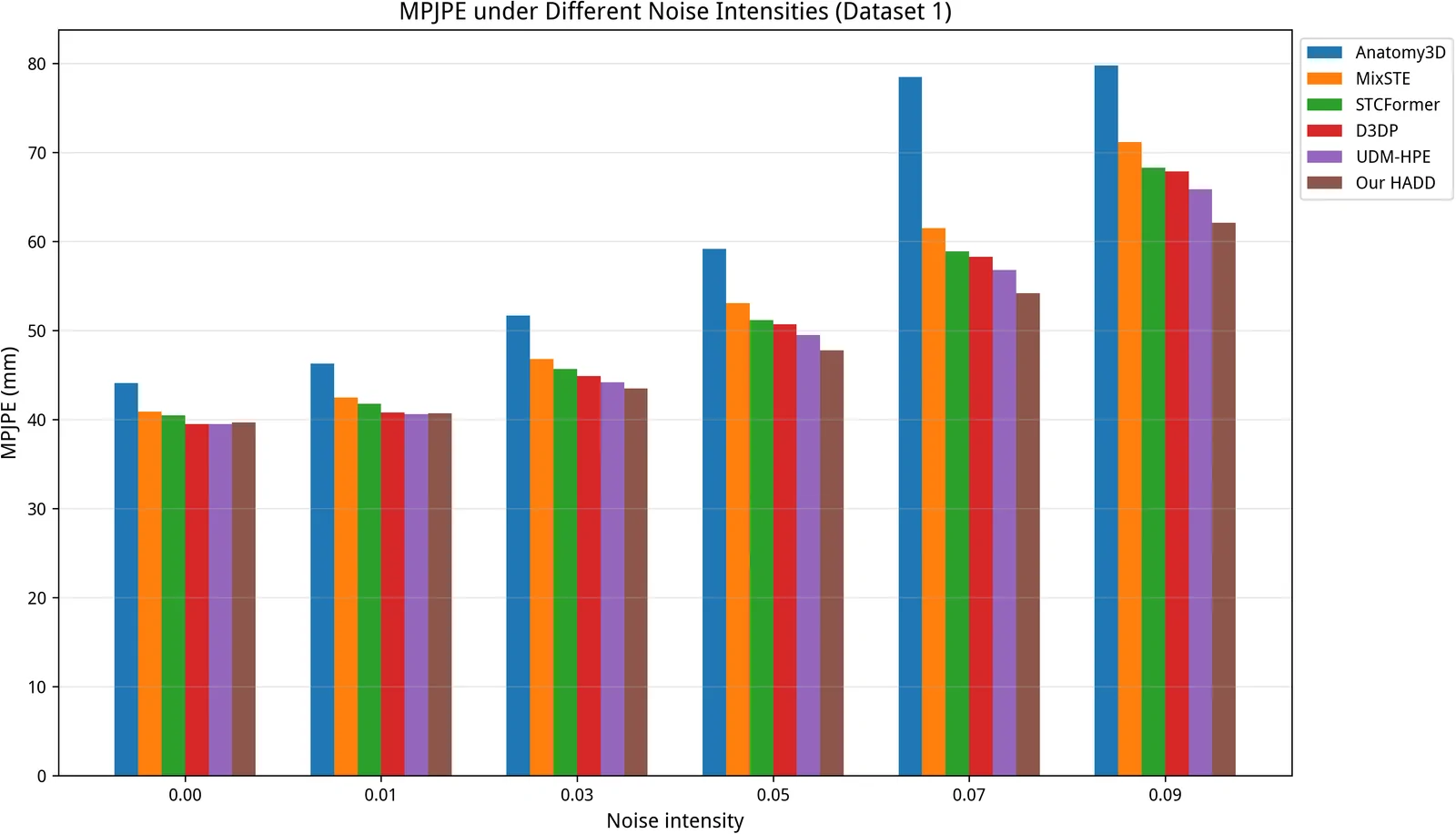
MPJPE (mm) of different methods against Gaussian noise in 2D input on Human3.6M.

**Fig 16:**
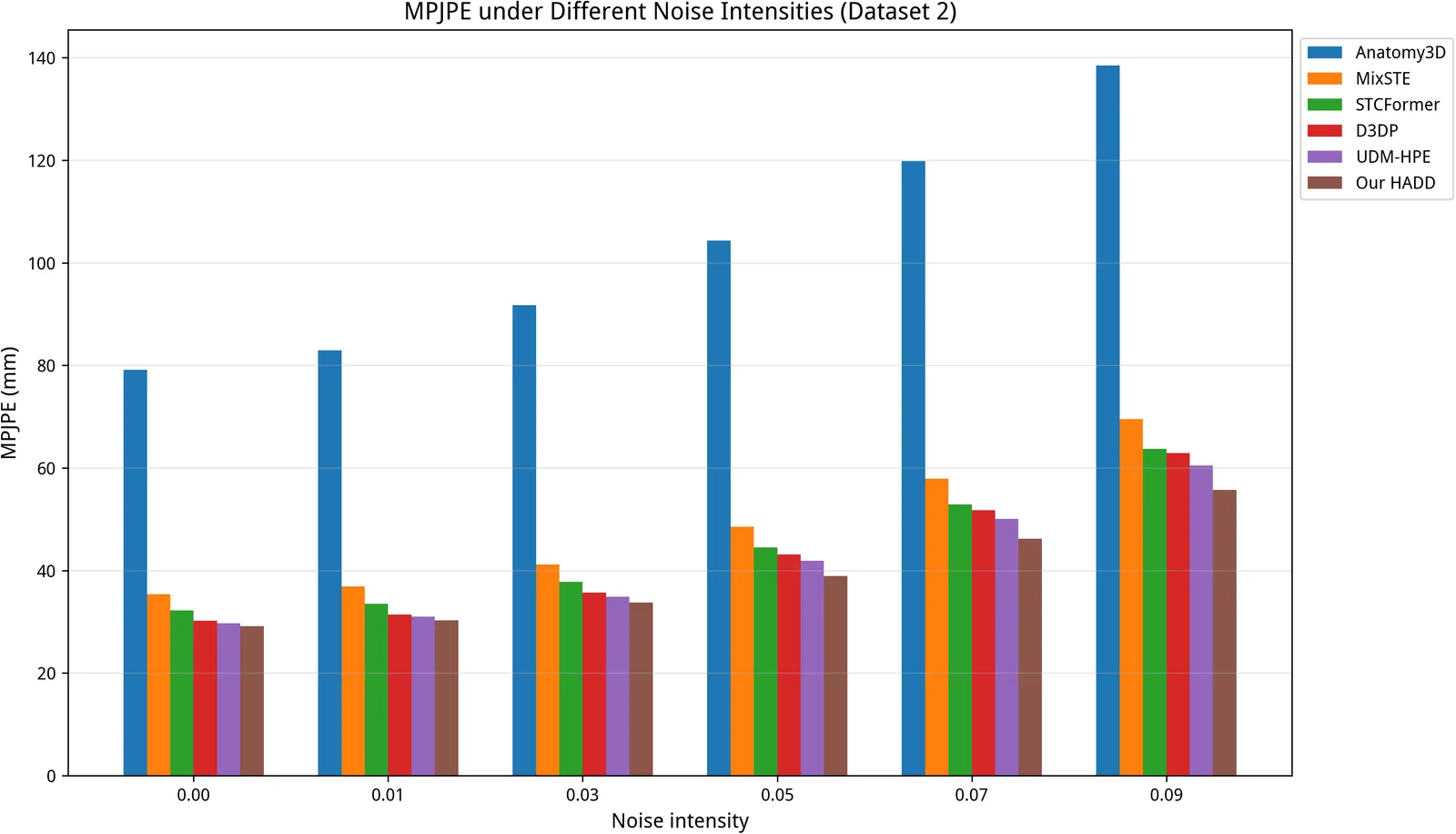
MPJPE (mm) of different methods against Gaussian noise in 2D input on MPI-INF-3DHP.

The results show that HADD exhibits the strongest noise suppression capability. On the Human3.6M dataset, when the noise intensity reaches σ=0.09, the MPJPE of HADD is 62.1 mm, which is 3.8 mm lower than UDM-HPE (65.9 mm) and 9.1 mm lower than MixSTE (71.2 mm). On the MPI-INF-3DHP dataset, the MPJPE of HADD is 55.7 mm at σ=0.09, which is 4.8 mm lower than UDM-HPE (60.5 mm) and 82.8 mm lower than Anatomy3D (138.5 mm). Even in the mild noise (σ=0.01) scenario, the performance of HADD is superior to all baseline methods, and its advantage continues to expand with the increase of noise intensity.

This result verifies the synergistic effect of the inherent denoising property of diffusion models and the HSTD module: diffusion models can recover clean pose distributions from noisy inputs, while the HSTD module further filters noise interference by modeling hierarchical spatio-temporal correlations between joints. Meanwhile, the bone length and bone direction priors constrained by the disentanglement loss ensure that the predicted pose always conforms to human anatomical laws, avoiding unreasonable limb deformations caused by noise.

Random coordinate zeroing is an idealized perturbation method and cannot fully reflect the failure modes of real-world 2D detectors. In practical applications, low-confidence outputs from detectors usually correspond to occluded, blurred, or truncated regions rather than randomly distributed joints. For this reason, we supplemented the keypoint dropout experiment based on CPN detector confidence, which is currently the standard realistic perturbation protocol for evaluating occlusion robustness in the 3D HPE field.

In the experiment, we used the original output confidence scores of the CPN detector on the two datasets, dropped 10%, 20%, 30%, and 40% of keypoints in ascending order of confidence scores (set their coordinates to zero), and retained the same single-hypothesis inference configuration as the random occlusion experiment. The detailed quantitative results are shown in Table 16.

**Table 16:** Comparison of MPJPE (mm) in Detector Confidence-Based Keypoint Dropout Experiments (Mean±SD [95%CI]).

Method	0% Drop	10% Drop	20% Drop	30% Drop	40% Drop
Human3.6M Dataset					
Anatomy3D	44.1±2.1 [42.8, 45.4]	47.6±2.3 [46.2, 49.0]	53.8±2.6 [52.2, 55.4]	62.1±2.9 [60.3, 63.9]	72.5±3.4 [70.4, 74.6]
MixSTE	40.9±1.9 [39.7, 42.1]	43.5±2.0 [42.3, 44.7]	48.7±2.3 [47.3, 50.1]	56.2±2.6 [54.6, 57.8]	65.8±3.0 [63.9, 67.7]
STCFormer	40.5±1.9 [39.3, 41.7]	42.9±2.0 [41.7, 44.1]	47.3±2.2 [45.9, 48.7]	53.8±2.5 [52.2, 55.4]	62.5±2.9 [60.7, 64.3]
D3DP	39.5±1.8 [38.4, 40.6]	41.8±1.9 [40.6, 43.0]	46.2±2.1 [44.9, 47.5]	52.7±2.4 [51.2, 54.2]	60.9±2.8 [59.2, 62.6]
UDM-HPE	39.5±1.8 [38.4, 40.6]	41.5±1.9 [40.3, 42.7]	45.6±2.1 [44.3, 46.9]	51.8±2.4 [50.3, 53.3]	59.6±2.7 [57.9, 61.3]
Ours	39.7±1.8 [38.6, 40.8]	41.6±1.9 [40.4, 42.8]	44.9±2.0 [43.6, 46.2]	49.8±2.3 [48.4, 51.2]	56.3±2.6 [54.7, 57.9]
MPI-INF-3DHP Dataset					
Anatomy3D	79.1±4.7 [76.2, 82.0]	85.2±5.0 [82.1, 88.3]	95.7±5.5 [92.3, 99.1]	109.8±6.2 [105.9, 113.7]	126.3±7.0 [121.9, 130.7]
MixSTE	35.4±2.2 [34.0, 36.8]	37.8±2.3 [36.4, 39.2]	43.1±2.6 [41.5, 44.7]	51.2±2.9 [49.4, 53.0]	61.5±3.4 [59.4, 63.6]
STCFormer	32.2±2.0 [30.9, 33.5]	34.3±2.1 [33.0, 35.6]	38.9±2.4 [37.4, 40.4]	45.8±2.7 [44.1, 47.5]	55.2±3.1 [53.2, 57.2]
D3DP	30.2±1.8 [29.1, 31.3]	32.1±1.9 [30.9, 33.3]	36.0±2.1 [34.7, 37.3]	42.5±2.4 [41.0, 44.0]	51.2±2.8 [49.4, 53.0]
UDM-HPE	29.7±1.7 [28.7, 30.7]	31.5±1.8 [30.4, 32.6]	35.2±2.0 [33.9, 36.5]	41.3±2.3 [39.9, 42.7]	49.1±2.7 [47.4, 50.8]
Ours	29.2±1.7 [28.2, 30.2]	30.7±1.8 [29.6, 31.8]	34.0±2.0 [32.7, 35.3]	39.2±2.2 [37.8, 40.6]	46.5±2.6 [44.9, 48.1]

The experimental results show that the performance degradation of all methods under confidence-based dropout is milder than that under random occlusion, because the dropped keypoints are those with inherently poor detection quality rather than high-quality reliable joints. Even so, HADD still maintains a significant performance advantage. On the Human3.6M dataset, the MPJPE of HADD is 49.8 mm at 30% keypoint dropout, which is 2.0 mm lower than UDM-HPE (51.8 mm); at 40% dropout, it is 56.3 mm, which is 3.3 mm lower than UDM-HPE (59.6 mm). On the MPI-INF-3DHP dataset, the MPJPE of HADD is 39.2 mm at 30% dropout, which is 2.1 mm lower than UDM-HPE (41.3 mm); at 40% dropout, it is 46.5 mm, which is 2.6 mm lower than UDM-HPE (49.1 mm).

The three types of perturbation experiments designed in this study (random zeroing, Gaussian noise, confidence dropout) constitute a rigorous stress test system, verifying the inherent anti-interference ability of HADD under input noise and partial occlusion. Among them, the keypoint dropout experiment based on detector confidence is closer to the failure mode of real 2D detectors, indicating that HADD has better robustness under such common degradation scenarios. However, it should be clearly stated that these experiments are still conducted in controlled dataset environments and do not cover challenges such as complex lighting changes, multi-person interactions, and severe full-body occlusion in unconstrained in-the-wild scenarios. Therefore, this study does not claim that HADD has general real-world robustness, and its generalization ability in unconstrained in-the-wild scenarios still needs further verification.

### 6.6. Visual analytics

To intuitively verify the effectiveness of HADD in 3D human pose estimation, we conducted a visual comparison between HADD and mainstream SOTA methods on typical action sequences of the Human3.6M test set S11 in Fig 17. Both 3D spatial views and 2D projection views of the 3D poses are presented, with comparisons focusing on three core aspects: the spatial accuracy of joint positions, the continuity of limb movements, and the estimation precision of high-hierarchy joints (hands and head). In the experiment, all methods adopted a unified input configuration (243-frame 2D joint sequences) and a single-hypothesis inference setting (*H* = 1, *Z* = 1) to ensure the fairness of the visual results; the predicted 3D joint coordinates were uniformly centered on the pelvis root joint and normalized in scale to eliminate the interference of global position and scale differences on visual comparisons.

**Fig 17:**
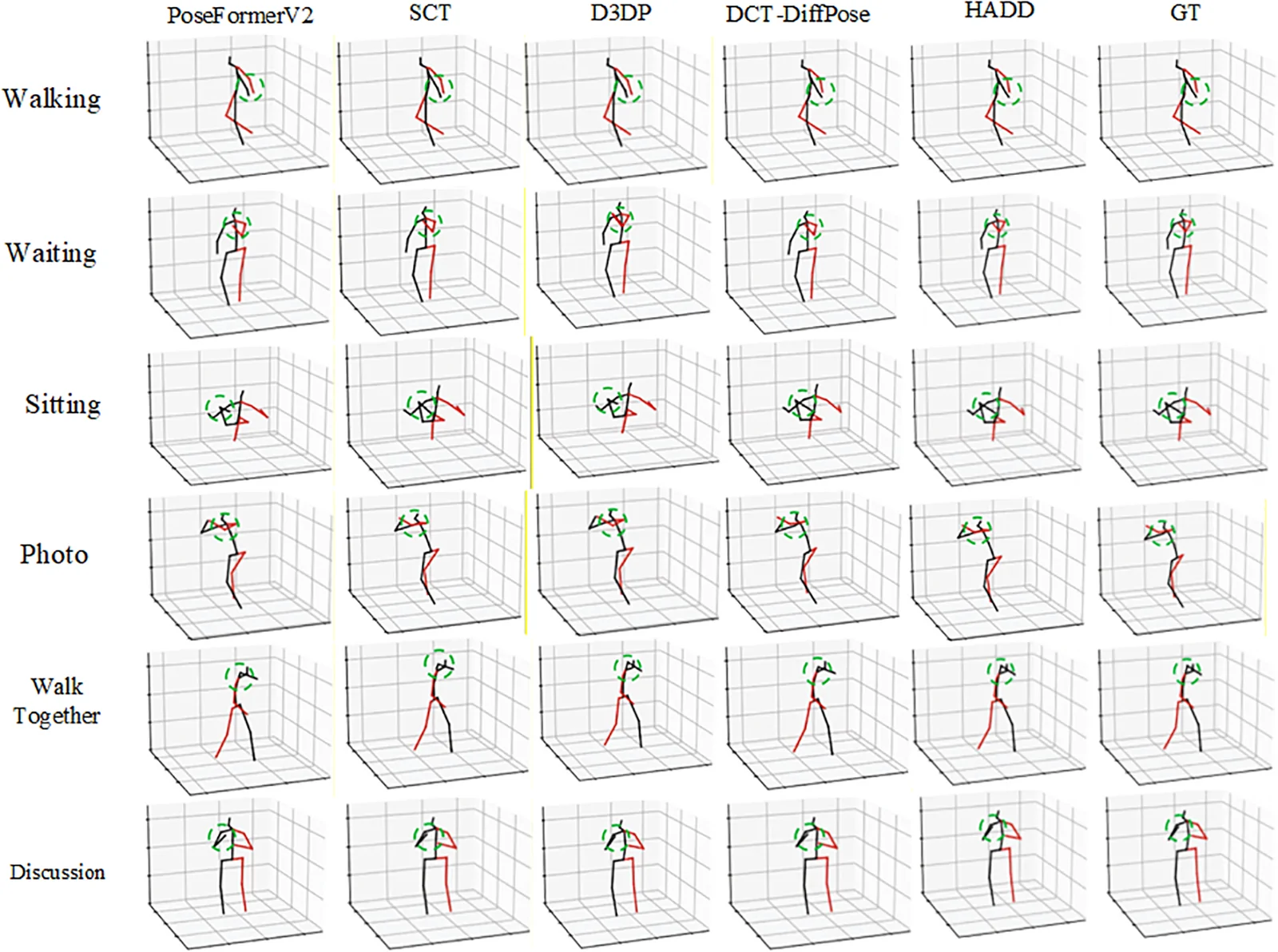
Qualitative results between our method and SOTA on Human3.6M test set S11. All original images from the Human3.6M dataset are removed. This figure is independently generated from raw 3D joint coordinates, and only human skeletons are presented for illustration.

The predicted results of HADD have no obvious joint overlap or position confusion in the 2D projection view, while the comparison methods all have varying degrees of depth estimation ambiguity. This further verifies that HADD effectively alleviates the depth ambiguity problem in monocular 3D human pose estimation through the disentangled diffusion strategy and hierarchical spatio-temporal modeling, realizing more physically reasonable and visually realistic 3D pose prediction. Overall, the visualization results are highly consistent with the quantitative experimental conclusions, intuitively demonstrating the comprehensive advantages of HADD in the detailed fitting of 3D human poses, movement continuity, and high-hierarchy joint estimation. The predicted 3D poses not only comply with the physical constraints of human anatomical structures but also match the dynamic laws of real human movements.

### 6.7. Computational efficiency

To comprehensively assess the practical applicability of the proposed HADD method, we conduct a detailed computational efficiency analysis on the Human3.6M dataset, alongside a comparison with state-of-the-art 3D human pose estimation methods (including disentangle-based, non-disentangle-based, and diffusion probabilistic approaches). We measure the core efficiency metrics of all compared methods under a unified experimental environment (single NVIDIA A100 80GB GPU, CUDA 11.7, PyTorch 2.0.1, mixed-precision FP16 training and inference). For the training phase, all models were uniformly trained for 300 epochs with an effective batch size of 32 (batch size 8 + 4-step gradient accumulation), and all diffusion models adopted a cosine noise schedule with 1000 total diffusion steps. All experiments are performed on a unified hardware platform. We evaluate four core efficiency metrics: FLOPs (Floating-Point Operations) (G), inference time per frame (ms), parameter counts and training time, as shown in Table 17. Only the pure computational time for end-to-end model training is measured, excluding data loading, logging, and model checkpoint writing time. For the inference phase, a typical single-video deployment configuration with a batch size of 1 is adopted. All diffusion-based methods in Table 17 use the lightweight single-hypothesis single-step inference setting (number of hypotheses *H* = 1, DDIM sampling steps *Z* = 1). All metrics are measured exclusively for the 2D-to-3D pose lifting module, excluding 2D pose detection, data preprocessing, and postprocessing steps, ensuring exactly the same statistical scope as all baseline methods.

**Table 17:** Computational efficiency and performance comparison on Human3.6M.

Method	FLOPs (G)	Inference Time (ms/frame)	Parameters (M)	Training Time (h)
Anatomy3D (*N* = 243)	18.6	42.3	1.65	23.3
Virtual Bones (*N* = 243)	20.1	45.7	1.78	25.5
MixSTE (*N* = 243)	15.3	32.5	1.82	29.7
STCFormer (*N* = 243)	16.7	35.8	2.01	33.1
D3DP (*N* = 243, *H* = 1, *Z* = 1)	28.5	59.7	3.12	54.2
DCT-DiffPose (*N* = 243, *H* = 1, *Z* = 1)	25.8	54.9	2.89	58.8
GFPose (*N* = 243)	24.7	52.1	2.75	62.2
Ours (*N* = 243, *H* = 1, *Z* = 1)	22.4	48.9	2.37	49.4

While HADD is slower and has higher computational complexity than several deterministic baselines (MixSTE, STCFormer, Anatomy3D, Virtual Bones), it outperforms all diffusion/probabilistic baselines in both inference speed and FLOPs, while delivering superior or comparable pose estimation accuracy. This makes HADD the most efficient diffusion-based method among the compared approaches, with a single-frame inference latency that meets the basic real-time requirements of many downstream applications. In summary, HADD does not achieve absolute optimality across all dimensions, but it precisely addresses the core pain point of diffusion-based methods: “high accuracy but difficult deployment.” Compared with deterministic methods, it achieves a significant accuracy improvement at an acceptable efficiency cost. Compared with other diffusion-based methods, it maintains state-of-the-art (SOTA) accuracy while significantly reducing computational overhead.

### 6.8. Discussion

Experimental results and ablation studies fully validate the effectiveness and superiority of our proposed HADD model for monocular 3D human pose estimation. Its core innovation lies in the deep integration of disentanglement strategy and diffusion model, which addresses two critical bottlenecks of existing mainstream methods: the lack of explicit human anatomical prior constraints in diffusion-based approaches, and severe hierarchical error accumulation in disentanglement-based methods. In the forward diffusion process, we disentangle 3D human pose into bone length and bone direction, and apply Gaussian noise to the two features separately instead of the whole 3D joint coordinates. This design enables the model to independently learn the temporal consistency of bone length and spatial variation rules of bone direction, efficiently embedding human anatomical priors and alleviating depth ambiguity in 2D-to-3D mapping. Benefiting from the improved accuracy of high-hierarchy terminal joints and the robustness to dynamic motions, the framework can provide reliable quantitative data support for competitive sports technique diagnosis, sports rehabilitation gait assessment, and youth physical fitness testing.

Unlike traditional disentanglement methods that directly regress bone parameters and reconstruct poses via forward kinematics, we confine disentanglement to the forward diffusion stage and take joint coordinates as the final optimization target in the reverse process, completely avoiding hierarchical error propagation along the skeletal kinematic tree. In the reverse diffusion process, our Hierarchical Spatial and Temporal Denoising (HSTD) module explicitly models the hierarchical correlation of parent-child joints via two sub-modules: HSDM strengthens the spatial attention weight of parent joints to child joints to characterize hierarchical spatial dependency, while HTDM fuses global temporal context and hierarchical temporal correlation between target and child joints to capture the linkage motion law of joints. The alternate iteration of the two modules achieves fine-grained modeling of hierarchical spatio-temporal information and effectively mitigates error accumulation in high-hierarchy joints. Our hybrid loss function combining 3D disentanglement loss and 3D pose loss realizes dual constraints at the bone and joint levels. The disentanglement loss guides the model to learn anatomical priors for structural rationality, while the pose loss directly constrains joint coordinate regression for global spatial accuracy. Ablation results confirm that this dual supervision brings significant accuracy improvement, especially for the P-MPJPE metric. This dual-level supervision mechanism at bone and joint dimensions aligns well with the analytical logic of sports biomechanics, and can directly output structured kinematic parameters for movement technique analysis.

HADD also achieves a favorable balance between accuracy and computational efficiency. It outperforms or matches mainstream diffusion-based probabilistic methods with fewer hypotheses and iterations in inference, with reasonable single-frame latency and FLOPs to meet the real-time requirements of downstream applications such as VR/AR, digital human animation, intelligent video surveillance, as well as real-time action feedback in sports training and on-site competition motion capture. Meanwhile, the model only takes 2D joint sequences as input without additional auxiliary information, showing strong deployment compatibility. Although HADD shows superior anti-interference ability under three types of controlled perturbations, its performance still degrades significantly under extreme occlusion scenarios. In addition, this study did not evaluate the robustness of HADD on real in-the-wild datasets (such as 3DPW, Human3.6M in-the-wild), nor did it test its adaptability to outputs from different 2D detectors. For competitive sports scenarios, challenges such as high-speed motion blur, multi-person limb occlusion and complex field backgrounds also remain to be further adapted. These are key issues that need to be addressed in future work.

This work has several limitations verified by experimental results. First, the model degrades obviously under heavy occlusion. When the occlusion/keypoint dropout ratio reaches 40%, its MPJPE rises to 56.3 mm on Human3.6M and 49.3 mm on MPI-INF-3DHP, indicating limited compensation ability for severe occlusion. Second, all experiments adopt single-person datasets, so the model cannot adapt to multi-person interaction and mutual occlusion scenarios, and is difficult to directly apply to posture analysis in multi-person competitive sports. Third, tested only on laboratory datasets, the model shows poor resistance to complex real-world interference, as reflected by obvious error growth under high Gaussian noise. Fourth, this framework only focuses on 3D joint estimation and cannot support human mesh and surface reconstruction. In future work, we will add occlusion-aware modules, extend the model for multi-person tasks, adopt in-the-wild datasets to improve generalization, explore joint pose and mesh co-estimation, and carry out lightweight adaptation research for typical sports scenarios to support real-time posture analysis on training grounds and portable devices.

## 7. Conclusion

In this paper, we propose HADD to solve the core bottlenecks of existing methods. By disentangling 3D pose into bone length and bone direction for dimension-wise noise addition in the forward diffusion process, we embed human anatomical priors while avoiding error accumulation of traditional disentanglement methods. In the reverse process, our HSTD module models the hierarchical spatio-temporal dependency of parent-child joints to alleviate error amplification in high-hierarchy joints. Combined with a hybrid loss function, the model achieves dual optimization of structural prior learning and hierarchical spatio-temporal modeling, and provides technical support for sports motion analysis and rehabilitation effect assessment. Extensive experiments on Human3.6M and MPI-INF-3DHP datasets show that HADD achieves consistent performance improvement over existing SOTA methods: it reduces average MPJPE by up to 12.9% over traditional disentanglement-based methods, optimizes average MPJPE by 2.0% over non-disentangled deterministic methods, and outperforms mainstream diffusion-based probabilistic methods under lightweight inference configurations, with significant advantages in high-hierarchy joint estimation and dynamic action sequences, suitable for fine-grained capture of dynamic athletic movements. For future work, we will focus on different directions: improving occlusion robustness via multi-modal information fusion; enhancing generalization ability in unconstrained real-world scenarios with large-scale datasets and self-supervised learning; expanding the model to realize joint 3D pose and mesh estimation; performing lightweight optimization for edge devices and real-time applications, and exploring its targeted adaptation for sports training scenarios. In addition, models incorporating additional auxiliary information (such as heatmaps and depth priors) can achieve further performance improvements, which is an important direction for future work.
