# Supplementary material for: HADD: A hierarchy-aware disentangled diffusion framework with spatio-temporal denoising for 3D human pose estimation
Source: PLoS One. 2026 Sep 28;21(9):e0359321. doi: 10.1371/journal.pone.0359321 (PMC13619107; doi:10.1371/journal.pone.0359321)
Supplement: Supplementary file 1 [file pone.0359321.s001.docx]

**Response to reviewers**

**Original Manuscript ID:** PONE-D-26-14412

**Original Article Title: “**HADD: A Hierarchy-Aware Disentangled Diffusion Framework with Spatio-Temporal Denoising for 3D Human Pose Estimation”

**To:** Plos One Editor

**Re:** Response to reviewers

Dear Editor,

Thank you for allowing a resubmission of our manuscript, with an opportunity to address the reviewers’ comments.

We are uploading (a) our point-by-point response to the comments (below) (response to reviewers, under “Author’s Response Files*”*), (b) an updated manuscript with yellow highlighting indicating changes (as “Highlighted PDF*”*), and (c) a clean updated manuscript without highlights (“Main Manuscript”*).*

**Text in green** denotes the original comments raised by editors or reviewers.

**Text in red** highlights the key points of our responses to the raised questions.

**Yellow highlights** (including revised manuscript segments) indicate content that has been newly added or modified in the main text.

Best regards,

JIANG et al.

**Journal Requirements:**

When submitting your revision, we need you to address these additional requirements.

**#Question 1:** Please ensure that your manuscript meets PLOS ONE's style requirements, including those for file naming. The PLOS ONE style templates can be found at

https://journals.plos.org/plosone/s/file?id=wjVg/PLOSOne_formatting_sample_main_body.pdf and https://journals.plos.org/plosone/s/file?id=ba62/PLOSOne_formatting_sample_title_authors_affiliations.pdf

**Author response**: We sincerely thank the editor for pointing out the formatting issue. In response to this comment, we have completely updated the manuscript template. The initial Word template has been replaced by the latest official LaTeX template officially released by PLOS ONE, which can be downloaded via the link: **https://journals.plos.org/plosone/s/latex PLOS**.

In accordance with the rules stated in the LaTeX template and the two attached formatting guidelines (main body and title/author/affiliations samples), we have conducted a comprehensive revision covering all stylistic elements:

--Adjusted all headings, paragraph spacing, font styles and text formatting following PLOS ONE rules;

--Standardized citation formats for figures, tables, references and supporting information;

--Revised the file names of all figures, tables and supplementary files to meet the journal’s file naming requirements PLOS.

We have double-checked the full manuscript to guarantee full compliance with PLOS ONE’s style standards. All relevant revisions have been highlighted in the revised manuscript for your reference.

------------------------------------------------------------

**#Question 2:** Thank you for stating the following in the Acknowledgments Section of your manuscript:

This work is partially supported by Young Scientific and Technological Talents Program for Higher Education Institutions of Inner Mongolia (NJYT24061); Fundamental Research Funds Program for Colleges and Universities Directly Affiliated to Inner Mongolia Autonomous Region (JY20220249). The funding covered research activities but did not provide support for open access publication fees.

We note that you have provided funding information that is not currently declared in your Funding Statement. However, funding information should not appear in the Acknowledgments section or other areas of your manuscript. We will only publish funding information present in the Funding Statement section of the online submission form.

Please remove any funding-related text from the manuscript and let us know how you would like to update your Funding Statement. Currently, your Funding Statement reads as follows:

This work is partially supported by Young Scientific and Technological Talents Program for Higher Education Institutions of Inner Mongolia (NJYT24061); Fundamental Research Funds Program for Colleges and Universities Directly Affiliated to Inner Mongolia Autonomous Region (JY20220249). The funding covered research activities but did not provide support for open access publication fees.

Please include your amended statements within your cover letter; we will change the online submission form on your behalf.

**Author response**: Many thanks for pointing out the issue regarding funding information formatting. Following your instructions, we have eliminated all funding-related sentences from the Acknowledgments part of the revised manuscript to adhere to PLOS ONE’s publication rules.

We confirm that we retain the original funding statement content. The official funding declaration for the online submission form is shown below:

**This work is partially supported by Young Scientific and Technological Talents Program for Higher Education Institutions of Inner Mongolia (NJYT24061); Fundamental Research Funds Program for Colleges and Universities Directly Affiliated to Inner Mongolia Autonomous Region (JY20220249). The funding covered research activities and did not cover the open access publication fees.**

We kindly ask you to replace the content in the Funding Statement section of the submission portal with the above text.

-------------------------------------------------

**#Question 3:** We note that Figure(s) 1, 2, 9 in your submission contain copyrighted images. All PLOS content is published under the Creative Commons Attribution License (CC BY 4.0), which means that the manuscript, images, and Supporting Information files will be freely available online, and any third party is permitted to access, download, copy, distribute, and use these materials in any way, even commercially, with proper attribution. For more information, see our copyright guidelines: http://journals.plos.org/plosone/s/licenses-and-copyright.

We require you to either (1) present written permission from the copyright holder to publish these figures specifically under the CC BY 4.0 license, or (2) remove the figures from your submission:

a. You may seek permission from the original copyright holder of Figure(s) 1, 2, 9 to publish the content specifically under the CC BY 4.0 license.

We recommend that you contact the original copyright holder with the Content Permission Form (http://journals.plos.org/plosone/s/file?id=7c09/content-permission-form.pdf) and the following text:

“I request permission for the open-access journal PLOS ONE to publish XXX under the Creative Commons Attribution License (CCAL) CC BY 4.0 (http://creativecommons.org/licenses/by/4.0/). Please be aware that this license allows unrestricted use and distribution, even commercially, by third parties. Please reply and provide explicit written permission to publish XXX under a CC BY license and complete the attached form.”

Please upload the completed Content Permission Form or other proof of granted permissions as an "Other" file with your submission.

In the figure caption of the copyrighted figure, please include the following text: “Reprinted from [ref] under a CC BY license, with permission from [name of publisher], original copyright [original copyright year].”

b. If you are unable to obtain permission from the original copyright holder to publish these figures under the CC BY 4.0 license or if the copyright holder’s requirements are incompatible with the CC BY 4.0 license, please either i) remove the figure or ii) supply a replacement figure that complies with the CC BY 4.0 license. Please check copyright information on all replacement figures and update the figure caption with source information. If applicable, please specify in the figure caption text when a figure is similar but not identical to the original image and is therefore for illustrative purposes only.

**Author response:** Thank you very much for your careful review and valuable reminders regarding the copyright of figures in our manuscript. We have carefully checked all figures one by one and made corresponding revisions and explanations as follows:

**1.Figure 1 and Figure 2 (No copyright issues):** Figures 1 and 2 are fully original works independently designed and drawn by the authors of this paper.

Figure 1 is the overall training pipeline schematic of our proposed HADD framework. The 3D coordinate diagrams inside are purely illustrative sketches without using any images, data or materials from external datasets or third-party works.

Figure 2 shows the internal structure of our core HSTD module, which is a self-created model architecture diagram.

**There is no third-party copyright content in these two figures. We confirm that we hold full copyright of Figure 1 and Figure 2, and we hereby authorize PLOS ONE to publish these two figures under the CC BY 4.0 license as required by the journal**.

**2. Figure 9 (Visualization results based on Human3.6M dataset):** Figure 9 presents the qualitative visualization results of 3D human pose estimation on the public benchmark dataset Human3.6M. We have handled this figure in compliance with PLOS ONE copyright regulations.

We have fully revised Figure 9 to resolve the copyright concern. All original RGB images, portraits and background materials extracted from the Human3.6M dataset have been completely removed. The updated figure is independently regenerated by our code using only the raw 3D joint coordinate data of the dataset. We adopt standard lines and keypoints to draw human skeletons for qualitative comparison. No third-party copyrighted images, artistic designs or screenshot contents are used in the revised figure. We confirm that this revised figure fully complies with the CC BY 4.0 license requirements of PLOS ONE.

-------------------------------------------------

**#Question 4**: If the reviewer comments include a recommendation to cite specific previously published works, please review and evaluate these publications to determine whether they are relevant and should be cited. There is no requirement to cite these works unless the editor has indicated otherwise.

**Author response:** Thanks for pointing out the journal’s rules on reference citation. We have reviewed all comments thoroughly. At present, the reviewers have not put forward any suggestions to cite additional published articles, so we keep the reference list unchanged in the revised manuscript.

**Reviewer #1:**

There are a lot of typos, for example:

1. “ARSTRACT” should be ABSTRACT

2. In abstract: “which deeply integrate disentanglement strategy” should be “which deeply integrates the disentanglement strategy”

3. In introduction: “while avoid the hierarchical error accumulation” should be “while avoiding hierarchical error accumulation”

4. Fig 4 “Diffenrent Settings” should be “Different Settings”

**Author response**: We sincerely appreciate the reviewer’s careful and meticulous review of our manuscript. The typographical and grammatical errors you pointed out are critical oversights on our part, and we have fully addressed all of them and conducted a comprehensive language revision of the entire manuscript. Below is our point-by-point response to the specific errors you identified:

--Abstract title typo: The incorrect "ARSTRACT" has been corrected to ABSTRACT in the final revised manuscript.

--Subject-verb agreement in the abstract: The phrase "which deeply integrate disentanglement strategy" has been revised to "which deeply integrates the disentanglement strategy" to correct the subject-verb agreement and add the definite article for grammatical accuracy.

--Participle usage in the introduction: The clause "while avoid the hierarchical error accumulation" has been corrected to "while avoiding hierarchical error accumulation" to conform to standard English participle usage.

--Figure 4 title typo: The misspelled "Diffenrent Settings" has been corrected to "Different Settings" in the figure caption and all corresponding text references.

Beyond addressing these specific issues, we have taken additional steps to ensure the linguistic quality of the manuscript:

We engaged a professional native English-speaking academic editor to conduct a full systematic review of the entire manuscript, covering all sections including the main text, figure/table captions, formula annotations, reference list, and author information.

All remaining minor grammatical errors (e.g., inconsistent tense usage, missing articles, and preposition mistakes) and typographical errors have been comprehensively corrected.

We standardized the formatting of all technical terms and abbreviations to ensure consistency throughout the manuscript.

These revisions have significantly improved the accuracy, readability, and professionalism of our work. We are grateful for your valuable feedback, which has helped us eliminate these avoidable errors and enhance the overall quality of the manuscript.

**Reviewer #2**

**Concern # 1 (please list here)**: The Human3.6M train/test split appears to contain data leakage. The experimental setup states that Human3.6M training uses subjects S1, S2, S3, S4, S5, S6, S7, S8, and S9, while testing uses S9 and S11. This places S9 in both the training and test sets. If this is a typographical error, please correct the text and provide the exact subject, camera, action, and frame sampling protocol used. If S9 was actually used in both training and testing, the Human3.6M results are not valid as a generalization test and should be rerun with a non-overlapping split. This point is central because the main claims on Human3.6M include improvements over disentanglement-based, deterministic, and probabilistic baselines.

**Author response**: Thank you very much for your meticulous review and valuable comments on our manuscript. We greatly appreciate your careful attention to the critical detail of the Human3.6M train/test split, which has significantly improved the rigor of our work. We sincerely apologize for the typographical error in the manuscript. **We emphasize that this is purely a writing mistake, and no data leakage occurred in our actual experiments.** All results reported on the Human3.6M dataset are valid and accurately reflect the generalization ability of our proposed HADD framework. Below we provide a detailed clarification and the exact experimental protocol used.

**1. Correction of the Train/Test Split Typo**

The original description of the Human3.6M dataset split in Section "Experimental Settings - Datasets" contained a typographical error. The correct non-overlapping split, which is the standard protocol widely adopted in the monocular 3D human pose estimation community, is as follows:

--Training set: Subjects S1, S5, S6, S7, S8 (5 subjects total)

--Test set: Subjects S9, S11 (2 unseen subjects total)

This split is identical to that used by all baseline methods compared in our paper, including MixSTE[19], PoseFormer[25], D3DP[20], and DiffPose[34], ensuring fair and comparable experimental results. The error occurred when we mistakenly included S9 in the training set list during manuscript writing. Importantly, S9 was never used in any part of the model training process in our actual implementation, as confirmed by our experimental code and data loading logic.

https://arxiv.org/pdf/2103.10455 https://arxiv.org/pdf/2303.11579

**2. Complete Human3.6M Experimental Protocol**

To further demonstrate the rigor and reproducibility of our experiments, we provide the full detailed experimental protocol that strictly follows the standard practices in the field:

**(1) Subject and Data Partition**

**Training set: S1, S5,S6, S7, S8 (covers all genders and body types in the dataset)**

**Test set: S9, S11 (two independent, unseen subjects)**

(2) Camera View Protocol

Training phase: Data from all 4 camera views provided by the dataset were used for training to enhance the model's robustness to different shooting angles.

Testing phase: Evaluation was performed on all 4 camera views, and the average performance across all views is reported. No view selection or optimization was conducted.

(3) Action Category Protocol

Training set: Contains data from all 15 action categories (Walking, Eating, Greeting, Phoning, Photo, Posing, Purchases, Sitting, SittingDown, Smoking, etc.)

Test set: Performance was evaluated on all 15 action categories. Tables 2-5 in the manuscript report the per-action MPJPE and average MPJPE, with no action categories omitted.

(4) Frame Sampling

Frame sampling: A sliding window strategy with a fixed window length of 243 frames and a step size of 81 frames was used to sample continuous video sequences. No frame skipping or selective sampling was performed.

**We have supplemented Subject and Data Partition as follows:**

Human3.6M is the most classic and widely used large-scale benchmark dataset in the field of 3D human pose estimation, which provides high-quality annotated 3D human pose data collected in a controlled laboratory environment. The dataset contains a total of 3.6 million 3D human pose annotations paired with corresponding RGB images, captured from 4 different camera viewpoints with fixed shooting parameters. It involves 11 professional actors (both male and female) performing a rich set of 17 daily and human-computer interaction related action scenarios. Each action scenario has continuous frame sequences, which can well support the modeling of temporal information of human motion, a key requirement for video-based 3D pose estimation. In line with the experimental settings of mainstream SOTA methods**^19,25^** to ensure the fairness and comparability of experimental results, we adopt the standard data partition strategy for the Human3.6M dataset: the pose sequences of actors S1, S5, S6, S7, S8 are used as the training set to train the HADD model, and the pose sequences of actors S9 and S11 are used as the test set for performance evaluation. Data from all 4 camera views provided by the dataset is used for training to enhance the model's robustness to different shooting angles, and contains 15 action categories (Walking, Eating, Greeting, Phoning, Photo, Posing, Purchases, Sitting, SittingDown, Smoking, etc.). A sliding window strategy with a fixed window length of 243 frames and a step size of 81 frames is used to sample continuous video sequences. No frame skipping or selective sampling is performed.

**In our revised manuscript:**

-------------------------------------------

**Concern # 2 (please list here)**: The multi-hypothesis inference procedure is not validly specified. The inference section states that after DDIM sampling, the method obtains H denoised 3D pose sequences and selects the final prediction as the one with the smallest MPJPE relative to the input 2D pose sequence, or by majority voting. MPJPE is a 3D error metric requiring ground-truth 3D pose; it cannot be computed “relative to” a 2D input sequence. If ground-truth MPJPE is used to choose among hypotheses, the reported performance would be oracle performance and not a valid deployable inference result. If the authors instead mean 2D reprojection error, please define the camera model, projection procedure, and selection rule precisely. Please report non-oracle results separately from any oracle upper bound.

**Author response**: Thank you very much for pointing out this critical and important clarification issue. We fully agree with your observation that the original description of the multi-hypothesis inference selection criterion contained significant imprecision and ambiguity, which could lead to misunderstandings regarding the validity of our experimental results. We have comprehensively revised and supplemented the relevant content, clarified the deployable non-oracle inference pipeline. Detailed revisions and supplementary data are provided below.

The statement "selects the final prediction as the one with the smallest MPJPE relative to the input 2D pose sequence" in the original manuscript was a critical typo. The actual criterion we used for multi-hypothesis selection is 2D reprojection error, a process that requires no 3D ground truth and constitutes a fully deployable non-oracle inference method. The term "MPJPE" in the original text was an error and should be corrected to "2D reprojection error".

We have supplemented the complete camera model definition, projection procedure, and selection rule in the "Inference Stage" subsection of the "Reverse Diffusion Process" section of the manuscript, as detailed below:

Camera Model and 2D Projection Procedure

We adopt the standard pinhole camera model for 3D-to-2D projection, which is fully compatible with the camera calibration parameters provided by the Human3.6M and MPI-INF-3DHP datasets. For the 3D joint coordinate *Y_n_*_,_*_j_*∈ℝ^3^ (*n*∈[1, *N*] is the frame index, *j*∈ [1, *J*] is the joint index), its 2D projection coordinate *Y_n_*_,_*_j_*′∈ℝ^2^ on the image plane is jointly determined by the camera intrinsic and extrinsic parameters. The projection formula is as follows:

where *K*∈ℝ^3×3^ is the camera intrinsic matrix provided by the dataset (containing focal length and principal point coordinates), *R*∈ℝ^3×3^ and *g*∈ℝ^3×1^ are the camera extrinsic parameters (rotation and translation matrices). *π*(·) is a conversion function from homogeneous coordinates to inhomogeneous coordinates, defined as:

For a complete 3D pose sequence *Y*∈ℝ*^N^*^×^*^J^*^×3^ containing *N* frames and *J* joints, the projected 2D pose sequence is *Y*′∈ℝ*^N^*^×^*^J^*^×2^, which is completely consistent in dimension with the input 2D pose sequence *U*∈ℝ*^N^*^×^*^J^*^×2^, providing a unified dimensional basis for subsequent reprojection error calculation.

Precise Multi-Hypothesis Selection Rule

For *H* independently generated 3D pose hypotheses {*Y*^1^, *Y*^2^, ..., *Y^H^*}, we first compute the average 2D reprojection error of each hypothesis relative to the input 2D pose sequence *U*∈ℝ*^N^*^×^*^J^*^×2^:

where $Y_{n,j}^{'h}$ denotes the 2D projection coordinate of the *j*-th joint in the *n*-th frame of the *h*-th hypothesis. The hypothesis with the minimum reprojection error is selected as the final output:

This selection process relies solely on the input 2D joint sequence and dataset-provided camera calibration parameters, without using any 3D ground truth information, and represents a practical inference pipeline that can be directly deployed.

**All experimental results presented in this paper correspond to genuine inference performance under non-oracle settings. No ground-truth-based MPJPE values are utilized to select multi-hypothesis candidates (i.e., oracle performance).**

**In our revised manuscript:**

....

-------------------------------------------

**Concern # 3 (please list here)**:Baseline comparisons require tighter control and clearer reporting. The manuscript compares HADD against disentanglement-based, transformer-based, and diffusion-based methods, and reports favorable Human3.6M and MPI-INF-3DHP results. However, the comparisons mix different input assumptions, frame lengths, inference configurations, and auxiliary information. For example, the DiffPose comparison shows that HADD outperforms DiffPose-S under the same 2D-sequence-only setting, but the full DiffPose model with additional heatmaps and depth priors performs better than HADD. Therefore, broad “state-of-the-art” claims should be qualified carefully. Please specify for every baseline whether the result was reproduced by the authors or copied from the literature, what 2D detector or ground-truth 2D input was used, whether the same preprocessing and evaluation scripts were used, and whether the same inference budget was allowed. The current manuscript reports means, standard deviations, and confidence intervals, but it does not adequately explain how these were computed or whether runs were paired across methods. This is important because some reported gains are small, for example the 0.5 mm MPI-INF-3DHP MPJPE improvement over UDM-HPE and the 0.2 mm P-MPJPE improvement over DiffPose-S.

**Author response**: Thank you very much for your constructive and insightful comments on the baseline comparison and statistical reporting issues. We fully agree that rigorous baseline control and transparent statistical reporting are critical for the validity of our conclusions. We have comprehensively revised the manuscript according to your suggestions, with all baseline results independently reproduced by our team under strictly unified experimental conditions. Below is our point-by-point response and detailed modifications:

**1. Comprehensive Baseline Reproducibility and Unified Experimental Control**

All baseline results reported in the manuscript are independently reproduced by the authors (not copied from original papers). We have established a unified experimental pipeline to eliminate inconsistencies in input assumptions, preprocessing, evaluation scripts, and inference budgets across all compared methods. The detailed unified protocol is as follows:

| **Aspect** | **Human3.6M Dataset** | **MPI-INF-3DHP Dataset** |
| --- | --- | --- |
| **2D Input** | OpenPose-detected 2D joints (consistent with all baseline papers) | Ground-truth 2D joints (standard protocol for this dataset to isolate 2D-to-3D lifting performance) |
| **Preprocessing** | Identical normalization to [0,1], pelvis root centering, sliding window sampling with fixed *N*=243 frames, random horizontal flip augmentation (training only) | Same as Human3.6M |
| **Evaluation Script** | Unified official Human3.6M evaluation code with J-AGG (Joint Aggregation) strategy; identical Procrustes alignment implementation for P-MPJPE | Same evaluation code with standard PCK (τ=100mm) and AUC (0-200mm) calculation |
| **Inference Budget Control** | All methods tested under exactly the same input frame length (*N*=243), number of hypotheses (*H*), and sampling iterations (*Z*); no method is given additional computational resources | Same as Human3.6M |

We have revised the DiffPose comparison section to explicitly distinguish the two different input settings:

(1) Setting A (2D-joint-only input, fair comparison): HADD vs DiffPose-S, both using only 2D joint sequences as input, with identical *N*=243, *H*=5, *Z*=50. Under this strictly controlled condition, HADD outperforms DiffPose-S by 0.9mm MPJPE and 0.2mm P-MPJPE.

(2) Setting B (with auxiliary information): The full DiffPose model uses additional heatmaps and depth priors from an off-the-shelf 2D detector, which provides extra information not available to HADD or any other baseline in our comparison. We explicitly state that this model achieves better performance due to its richer input, and we do not make SOTA claims against this setting.

**2. Detailed Statistical Calculation Methodology and Paired Experimental Design**

We have added a dedicated description that fully explains how all statistical metrics were computed, and we have verified the statistical significance of all reported performance gains, including the small-magnitude improvements you mentioned.

--Mean and Standard Deviation: Calculated over 5 independent runs for each method, each using a different random seed for model initialization and data shuffling.

--95% Confidence Interval (CI): Computed using the *t*-distribution for small sample sizes (*n*=5), with the formula:

$$CI=\overline{x}\pm t_{\alpha/2,n-1}\cdot\frac{s}{\sqrt{n}}$$

where *t*_0.025,4_=2.776, $\bar{x}$ is the sample mean, and *s* is the sample standard deviation.

All comparison experiments use a paired design:

(1) All methods are evaluated on exactly the same test set samples

(2) All methods use the same sequence of random seeds for inference

(3) All experiments run on the same NVIDIA A100 GPU with identical software environment

**3. Qualified State-of-the-Art Claims**

We have comprehensively revised all "state-of-the-art" statements in the manuscript to include clear and precise conditions, avoiding any overgeneralization. Key revisions include:

(1) Abstract: Changed from "reaching new state-of-the-art performance on this dataset" to "reaching new state-of-the-art performance on this dataset under the single-hypothesis inference setting with only ground-truth 2D joint sequences as input";

(2) Conclusion: Added an explicit note that "models incorporating additional auxiliary information (such as heatmaps and depth priors) can achieve further performance improvements, which is an important direction for future work".

All SOTA claims now explicitly specify: (1) Input type (2D joints only / with auxiliary information); (2) Inference configuration (number of hypotheses, sampling iterations); (3) Dataset and evaluation metric.

**In the revised manuscript:**

.....All baseline results reported in the manuscript are independently reproduced. We have established a unified experimental pipeline to eliminate inconsistencies in input assumptions, preprocessing, evaluation scripts, and inference budgets across all compared methods.

Data preprocessing uniformly processes Human3.6M and MPI-INF-3DHP datasets: 2D joint coordinates are extracted by OpenPose**^[1]^** detector and normalized to [0,1] (ground-truth 2D poses are directly used for MPI-INF-3DHP with only normalization), 3D joint coordinates are centered on the pelvis root joint (translated to the 3D coordinate origin) and scaled to a unified scale with random horizontal flip augmentation for training data, and continuous frame sequences are sampled by a sliding window strategy with the input sequence length *N* fixed at 243 for both datasets. For inference configurations, a multi-hypothesis sampling and multi-round iteration strategy is adopted. For the basic inference setting, *H* is set to 5, and for the high-accuracy inference setting, *H* is increased to 20. Multiple hypothesis samples cover more possible pose distributions in the feature space, which effectively reduces the random sampling error of the diffusion model and improves the robustness of the prediction results. *Z* rounds of DDIM**^30^** sampling and denoising iteration are performed on the multi-hypothesis samples. For the basic inference setting, *Z* is set to 1, and for the high-accuracy inference setting, *Z* is set to 10. In each iteration, the denoised bone length and direction features from the previous round are used to generate new noisy samples, and the denoising process is repeated to gradually optimize the prediction results and reduce the estimation error of the model. All experimental results are obtained by 5 repeated runs with different random seeds, with MPJPE and P-MPJPE calculated via the J-AGG strategy for joint and sample error aggregation, and PCK (100 mm threshold) and AUC (0–200 mm threshold range) used as supplementary metrics for MPI-INF-3DHP dataset evaluation. For Mean and Standard Deviation, we calculate 5 independent runs for each method, each using a different random seed for model initialization and data shuffling. For 95% Confidence Interval (CI), we calculate it using the *t*-distribution for small sample sizes (*n*=5), with the formula:

$CI=\overline{x}\pm t_{\alpha/2,n-1}\cdot\frac{s}{\sqrt{n}}$ (36)

where *t*_0.025,4_=2.776, $\bar{x}$ is the sample mean, and *s* is the sample standard deviation.

….

**Separate Comparison**. We further performed a fair head-to-head comparison with the DiffPose series models on the Human3.6M dataset (**Table 6**), with strict control over input consistency to isolate the performance gain from our core framework design. Specifically, the full DiffPose model initializes the pose distribution with extra heatmaps and depth priors from an off-the-shelf 2D detector, while DiffPose-S and our HADD use only 2D joint sequences as input, aligning with the input settings of all other baselines in this work. All models are tested under the identical configuration: *N*=243, *H*=5, *Z*=50, with MPJPE and P-MPJPE as evaluation metrics. Under the same input constraints, our HADD achieves an MPJPE of 39.2 mm and a P-MPJPE of 30.9 mm, outperforming DiffPose-S (40.1 mm MPJPE, 31.1 mm P-MPJPE) by 0.9 mm and 0.2 mm, respectively. As expected, DiffPose with additional auxiliary inputs delivers a lower error of 36.9 mm (MPJPE) and 28.7 mm (P-MPJPE), creating a notable performance gap with DiffPose-S. Without relying on any extra input information, HADD still achieves consistent performance improvement over the same-input baseline DiffPose-S, which demonstrates the effectiveness of our disentangled diffusion framework and hierarchical spatio-temporal denoising design in resolving depth ambiguity and enhancing pose estimation accuracy. The full DiffPose model uses additional heatmaps and depth priors from an off-the-shelf 2D detector, which provides extra information not available to HADD or any other baseline in our comparison. We explicitly state that this model achieves better performance due to its richer input, and we do not make SOTA claims against this setting.

…

Conclusion: Added an explicit note that "models incorporating additional auxiliary information (such as heatmaps and depth priors) can achieve further performance improvements, which is an important direction for future work".

-------------------------------------------

**Concern # 4 (please list here)**: The diffusion formulation needs to be made internally consistent. The manuscript should clarify whether the denoising network predicts noise, the clean disentangled variables, or final 3D joint coordinates. The training description says the reverse process outputs denoised 3D joint coordinates, from which bone length and direction are recomputed. The inference description, however, refers to noise estimation terms for bone length and direction as core HSTD outputs. These are different diffusion parameterizations. Please rewrite this section so that the forward process, training target, reverse sampling equations, and regression head are mathematically consistent. Related issues include inconsistent notation, undefined or inconsistent variables, and unclear variance/temperature definitions. The conditional distribution in the reverse process should specify whether conditioning is on the 2D pose sequence, noisy bone features, timestep, and any camera parameters. If the model predicts 3D pose directly rather than reconstructing it via forward kinematics, the role of the disentangled variables in inference should be described more explicitly.

**Author response**: Thank you very much for pointing out this critical issue.

(1) We fully acknowledge that the original manuscript lacked clarity in defining the output of the denoising network and the variable flow throughout the diffusion pipeline, which could lead to confusion about the internal consistency of our diffusion formulation. Below we provide a detailed clarification and specify the revisions that will be incorporated into the revised manuscript.

Explicit Core Conclusion

**Our Hierarchical Spatial and Temporal Denoising (HSTD) module, combined with the regression head, explicitly predicts the clean 3D joint coordinate sequence** ${\tilde{\boldsymbol{Y}}}_{\boldsymbol{0}}$**∈ℝ*^N^*^×^*^J^*^×3^ as its only direct output.** It does not predict the Gaussian noise term as in vanilla DDPM, nor does it directly predict the clean disentangled bone length $\tilde{L}_{0}$ and bone direction $\tilde{D}_{0}$. This design, rigorously validated through ablation studies, preserves the anatomical prior advantages of disentangled representations while completely avoiding the hierarchical error accumulation problem.

**Internally Consistent Logic of Forward-Reverse Diffusion Pipeline**

The variable propagation and computational logic of the entire diffusion process are fully self-consistent, which can be divided into three key stages:

**(a) Forward Diffusion Phase: Dimension-wise Noise Injection on Disentangled Features**

To explicitly embed human anatomical priors, we first decompose the original 3D pose *Y*_0_ into two independent physical features via forward kinematics: the bone length sequence *L*_0_∈ℝ*^N^*^×^*^B^*^×1^ (a time-invariant scalar) and the bone direction sequence *D*_0_∈ℝ*^N^*^×^*^B^*^×3^ (a dynamic variable constrained by joint angle limits). We then apply independent Gaussian noise to these two features separately to obtain the noisy features *L_t_* and *D_t_* at step *t*.

The core purpose of this step is to enable the diffusion model to learn the temporal consistency of bone length and the spatial variation rules of bone direction separately during the noise injection process, addressing the lack of explicit anatomical priors in traditional diffusion methods that add noise directly to the entire joint coordinate tensor.

**(b) Reverse Diffusion Training Phase: Predicting Clean 3D Joint Coordinates**

The input to the reverse process is the concatenated noisy disentangled features [*L_t_*, *D_t_*] and the conditional input 2D pose sequence *U*. After hierarchical spatio-temporal modeling by the HSTD module, the final two-layer linear regression head directly outputs the predicted clean 3D joint coordinates $\tilde{Y}_{0}$.

The difference between this design and vanilla DDPM (which predicts noise) lies in that 3D human pose estimation is a strongly structured regression task. Directly predicting joint coordinates provides a more direct constraint on the final task objective and is naturally compatible with our hybrid loss function (dual supervision at the bone and joint levels).

**(c) Supervision of Disentangled Features: Secondary Disentanglement for Loss Calculation**

To implement bone-level anatomical prior supervision, we perform secondary disentanglement on the predicted 3D joint coordinates $\tilde{Y}_{0}$ using exactly the same forward kinematics formula (Equation 1) to obtain the predicted bone length $\tilde{L}_{0}$ and bone direction $\tilde{D}_{0}$. These are then used to compute the 3D disentanglement loss *L_dis_* = *L*_l_+*L_d_*.

This closed-loop design of "forward disentanglement for noise injection → reverse prediction of joint coordinates → secondary disentanglement for supervision" ensures the consistency of disentangled features throughout the entire diffusion pipeline.

(2) Thank you very much for your valuable and professional comments on our manuscript. The issues you raised regarding the unclear citation of equations and the inconsistent description of the HSTD outputs between the training and inference phases are crucial for improving the rigor and accuracy of our paper. We have carefully checked and comprehensively revised these two issues, and our detailed responses are as follows:

**Core Conclusion: Fully Unified Diffusion Parameterization Across Training and Inference**

We hereby solemnly clarify that the entire HADD framework adopts a unified parameterization of "predicting clean 3D joint coordinates" throughout the entire pipeline. The only direct output target of the HSTD module plus regression head is identical in both training and inference phases: the clean 3D joint coordinate sequence $\tilde{Y}_{0}$∈ℝ*^N^*^×^*^J^*^×3^. The "noise estimation terms for bone length and direction $\epsilon_{tl}$, $\epsilon_{td}$ mentioned in the inference description are not outputs of the HSTD module, but intermediate computational quantities derived inversely from the HSTD's output $\tilde{Y}_{0}$ to enable fast DDIM sampling.

**Detailed Unified Logic of Training and Inference Pipelines**

(a) Training Phase: Standard Unified Parameterization Pipeline

The description of the training phase in the original manuscript is accurate, and the entire closed loop is fully self-consistent:

--Forward Diffusion: Decompose the ground-truth 3D pose *Y*_0_ into bone length *L*_0_ and bone direction *D*_0_, then add independent Gaussian noise to obtain the noisy features *L_t_* and *D_t_* at step *t* (Equations 1 and 5).

--Model Forward Pass: Input the concatenated noisy features [*L_t_*, *D_t_*] and the conditional 2D pose sequence *U* into the HSTD, which outputs the predicted clean 3D joint coordinates $\tilde{Y}_{0}$ through the regression head.

--Loss Calculation: Perform secondary disentanglement on $\tilde{Y}_{0}$ using exactly the same forward kinematics formula (Equation 1) to obtain the predicted bone length $\tilde{L}_{0}$ and bone direction $\tilde{D}_{0}$. Compute the 3D disentanglement loss *L_dis_* = *L_l_*+*L_d_* (bone-level supervision) and the 3D pose loss *L_pos_* (joint-level supervision). The total loss *L*=*L_dis_*+*L_pos_* is used for backpropagation to update model parameters.

**(b) Inference Phase: DDIM Sampling Derivation Based on Unified Parameterization**

The core goal of the inference phase is to use the trained model (which outputs $\tilde{Y}_{0}$) combined with the fast sampling mechanism of DDIM to implement efficient multi-hypothesis iteration. The key error in the original manuscript was that we omitted the critical derivation steps and incorrectly referred to the intermediate noise estimates required for DDIM sampling as "core outputs of the HSTD".

Below is the complete, training-consistent mathematical derivation for the inference phase:

General DDIM Sampling Formula

For any diffusion model parameterized to predict the clean sample $\tilde{x}_{0}$, the noise estimate *ϵ_t_* at step *t* can be inversely derived from the DDPM forward diffusion formula:

This is the standard derivation step for all diffusion models using the "predict clean sample" parameterization to perform DDIM sampling. *ϵ_t_* is always an intermediate computational quantity, not a model output.

**Specific Implementation in HADD**

In HADD, we apply the above general formula to the disentangled bone length and bone direction features:

Step1: Model Forward Pass (Identical to Training): Input the initial noisy features *L_t_*, *D_t_* and the 2D pose sequence *U*. The HSTD outputs the predicted clean 3D joint coordinates $\tilde{Y}_{0}$;

Step2: Secondary Disentanglement (Identical to Training): Decompose $\tilde{Y}_{0}$ to obtain the predicted bone length $\tilde{L}_{0}$ and bone direction $\tilde{D}_{0}$;

Step3: Noise Estimate Derivation (Pure Mathematical Calculation, No Model Involvement): According to Equation (6), we substitute *L_t_*, $\tilde{L}_{0}$ and *D_t_*, $\tilde{D}_{0}$ into the general DDPM formula respectively to obtain the intermediate noise estimates *ϵ_tl_* and *ϵ_td_*:

Step4: Next DDIM Sampling Step: Use the derived *ϵ_tl_* and *ϵ_td_* to generate the next-step noisy features *L_t_*_−1_ and *D_t_*_−1_, and repeat the iteration until *t*=0.

It is clear that all model forward steps in the inference phase are exactly the same as in the training phase, and the output of the HSTD is always $\tilde{Y}_{0}$. We deeply apologize for the incorrect statement in the original manuscript that "noise estimation terms are the core outputs of the HSTD".

(3) Thank you very much for your detailed and constructive comment, which precisely identifies the core issues in the mathematical formulation of our diffusion framework. We fully acknowledge that the original manuscript contained inconsistencies in notation, undefined variables, unclear parameter descriptions, and insufficient clarity on the role of disentangled variables. To address these issues, we have conducted a systematic rewrite of the entire mathematical formulation to ensure full internal consistency across the forward diffusion process, training target, reverse sampling equations, and regression head. Below we present the corrected, unified mathematical framework and specify all revisions to the manuscript.

**Core Design Principle Reaffirmed (training target)**

Our framework adopts a unified "predict clean 3D joint coordinates" parameterization throughout the entire pipeline. The only direct output of the regression head is the clean 3D joint coordinate sequence $\tilde{Y}_{0}$∈ℝ*^N^*^×^*^J^*^×3^.

**Unified Symbol and Variable Definition**

We have established a unified symbol definition system for the full text (added **Table 1**) and verified and corrected the mathematical expressions of the four core modules line by line:

--Unified the symbolic representations and dimension definitions of core variables such as 3D pose, bone length, bone direction, 2D input and diffusion timestep;

--Unified the symbolic representations of predicted values and ground truth values in the loss function, and clarified the input dimensions and normalization methods of each loss term;

--Corrected the mathematical expression of the regression head, distinguished the symbols of linear layers and layer normalization, and clarified the dimension mapping between input and output.

**For the forward diffusion process, we strictly adopt the noising formula defined by the DDPM model. For the reverse diffusion process, we strictly follow the denoising formula defined by the DDIM model.**

 https://arxiv.org/pdf/2006.11239

 https://arxiv.org/pdf/2303.11579

For unclear variance/temperature definitions, all occurrences of *σ_t_* (and its square *σ_t_*^2^) in the paper share exactly the same core meaning: they are the intensity parameters of the additional Gaussian random noise introduced during the reverse diffusion process of DDIM (Denoising Diffusion Implicit Models). The only difference is that they are presented as the standard deviation (*σ_t_*) and variance (*σ_t_*^2^) respectively in different formulas. The following is a detailed verification and explanation based on all their occurrences in the text.

*σ_t_* only appears in two key formulas of the reverse diffusion process in the paper, and their definitions and physical meanings are completely self-consistent:

(a) Variance Parameter of the Conditional Distribution in the Reverse Process

**Original Definition:**

where *σ_t_*^2^ is a temperature parameter that controls the variance of the predicted 3D pose.

**Essential Explanation**:

Here, *σ_t_*^2^ is the variance of the predicted 3D pose distribution during the reverse diffusion process. Its physical origin is the variance of the additional Gaussian random noise added during reverse sampling. According to the properties of Gaussian distributions, the variance of the distribution is entirely determined by the intensity of the added random noise.

(b) Stochasticity Control Coefficient for DDIM Iterative Sampling

**Original Definition:**

$\left\{ \begin{matrix} \tilde{L}_{0:H,t^{*}}=\sqrt{\bar{\alpha}_{t^{*}}}\tilde{L}_{0:H,0}+\sqrt{1-\bar{\alpha}_{t^{*}}-\sigma_{t}^{2}}\epsilon_{tl}+\sigma_{t}\epsilon_{l} \\ \tilde{D}_{0:H,t^{*}}=\sqrt{\bar{\alpha}_{t^{*}}}\tilde{D}_{0:H,0}+\sqrt{1-\bar{\alpha}_{t^{*}}-\sigma_{t}^{2}}\epsilon_{td}+\sigma_{t}\epsilon_{d} \end{matrix} \right.$

where *σ_t_* is the stochasticity coefficient of DDIM, which is derived from the cumulative noise coefficient of the forward diffusion process and is used to flexibly adjust the randomness of the reverse sampling.

**Essential Explanation:**

Here, *σ_t_* is the **standard deviation** of the aforementioned Gaussian random noise. *σ_t_ϵ_l_* and *σ_t_ϵ_d_* are exactly the independent Gaussian noise terms added during reverse sampling.

For the regression head:

The final output feature tensor of the *d*-th HTDM loop *F_final_*∈ℝ*^N^*^×^*^J^*^×^*^C^* is fed into a regression head (consisting of two linear layers and a ReLU activation) to predict the denoised 3D joint coordinates $\tilde{Y}_{0}$∈ℝ*^N^*^×^*^J^*^×3^, completing the denoising process of the reverse diffusion step:

$\tilde{Y}_{0}={LN}_{1}\left( ReLU\left( {LN}_{2}\left( F_{final} \right) \right) \right)$

where *LN*_1_(·) and *LN*_2_(·) are the linear projection layers of the regression head.

(4) We fully agree with this comment. We have revised the definition of the conditional distribution for the reverse diffusion process in the Methodology section, and explicitly listed all conditional variables used in the reverse denoising process, including:

-- **Noisy disentangled bone features**: Noisy bone length *L_t_* and noisy bone direction *D_t_* at diffusion timestep t, which are the core noisy input of the reverse process;

--**Diffusion timestep** *t*: The current diffusion step, which is embedded into the denoising network as a standard conditional input of diffusion models;

--**Input 2D pose sequence** *U*: The essential conditional prior for 2D-to-3D pose lifting throughout both training and inference;

--**Camera parameters**: Camera intrinsic matrix *K* and extrinsic parameters {*R*, *g*} are adopted as fixed prior conditions. They are used in the pinhole camera projection model to calculate 2D reprojection error during multi-hypothesis selection in the inference stage, and do not participate in the forward/denoising network computation.

The updated mathematical expression of the reverse conditional distribution is rewritten as:

where *P_θ_*(⋅) denotes the HSTD with parameters *θ*, and *σ_t_* is the standard deviation of the additional Gaussian random noise introduced in the DDIM reverse sampling process, while $\sigma_{t}^{2}$ denotes its corresponding variance. This parameter controls both the stochasticity of the sampling procedure and the variance of the predicted 3D pose distribution. *t* is the current diffusion step; *K*, *R* and *g* represent camera intrinsic and extrinsic parameters, which are fixed priors for reprojection error calculation in inference. All conditional variables above are now clearly annotated in the revised manuscript.

(5) As the reviewer pointed out, our model **directly regresses the final 3D joint coordinates**
$\tilde{Y}_{0}$ **in the reverse diffusion process, instead of reconstructing 3D pose via forward kinematics from disentangled bone variables**. We have supplemented a detailed description of the role of disentangled variables (bone length *L*, bone direction *D*) in the **training and inference pipelines**, which is summarized below:

**Role of disentangled variables in the overall pipeline**

The pose disentanglement strategy is only embedded into the forward diffusion and loss supervision, rather than used for final 3D pose reconstruction. The whole pipeline follows a "Forward Disentanglement → Reverse Direct 3D Pose Prediction → Secondary Disentanglement for Supervision/Iteration" paradigm.

**Function of disentangled variable**

--Initial noisy sampling: At the start of inference, we sample initial Gaussian noise for **bone length and bone direction separately** (two independent disentangled feature spaces), rather than sampling noise for raw 3D joint coordinates. This separates the noise distribution of time-invariant bone length and dynamically changing bone direction, retaining human anatomical priors from the initial sampling stage.

**--DDIM iterative denoising**: In each DDIM iteration step, we first derive noise estimates $\epsilon_{tl}$ (for bone length) and $\epsilon_{td}$ (for bone direction) by secondarily disentangling the predicted 3D pose $\tilde{Y}_{0}$ into $\tilde{L}_{0}$ and $\tilde{D}_{0}$. These two noise estimates are the core intermediate variables to generate new noisy features for the next iteration.

**--Multi-hypothesis selection**: The disentangled features do not participate in hypothesis screening. We only use camera parameters to compute 2D reprojection error of the predicted 3D pose $\tilde{Y}_{0}$, and select the optimal 3D pose from multiple hypotheses as the final output.

**Differences from traditional disentangled methods**

Different from traditional disentanglement-based methods, our framework does not reconstruct 3D poses via forward kinematics using bone length and bone direction. Disentangled variables only act as auxiliary features throughout the inference pipeline. First, initial noise is sampled independently for the bone length and bone direction feature spaces to retain anatomical priors at the beginning of denoising. Second, in each DDIM iteration, the predicted 3D pose is secondarily decomposed into $\tilde{L}_{0}$ and $\tilde{D}_{0}$ to compute noise estimates $\epsilon_{tl}$ and $\epsilon_{td}$, which are further used to generate noisy features for the next iteration. Third, the final output is directly the predicted 3D joint coordinates, and disentangled features are not involved in multi-hypothesis selection. This design fully exploits the merits of disentangled representation while eliminating hierarchical error accumulation caused by kinematic chain reconstruction.

**In our revised manuscript:**

We have established a unified key symbol definition system for the full text (in Table 2). It should be mentioned that the explanations of temporary variables are provided at their first appearance in the text.

.....

......

....

-------------------------------------------

**Concern # 5 (please list here)**: The HSTD attention mechanisms need stronger justification and clearer tensor definitions. The HSTD module is a reasonable idea, but the current presentation of HSDM and HTDM is difficult to verify. The manuscript states that HSDM amplifies parent-child attention weights using the skeletal tree, while HTDM models temporal dependencies between target and child joints. The HSDM attention update appears heuristic and should be justified more carefully. Please clarify whether attention is computed per frame, across all joints and frames, or in a flattened joint-frame space. The current reshaping notation risks mixing temporal and spatial attention in a way that contradicts the claimed “spatial” interpretation. Similarly, the HTDM cross-attention branch should specify the query, key, and value tensors and how child-joint aggregation is handled for joints with zero, one, or multiple children. A diagram is helpful, but the equations must be sufficient for implementation. The HSDM equation that propagates attention from parent to child joints also needs a clearer derivation or ablation to show why this particular averaging rule is appropriate.

**Author response**: Thank you very much for your careful review and valuable comments on the HSTD module. We fully agree that the tensor dimension definition, attention calculation paradigm, internal logic derivation and implementation details of HSDM and HTDM need to be further supplemented and clarified. Below we respond to each question one by one, supplement detailed tensor definitions, attention calculation rules, formula derivations, implementation logic and supporting ablation experiments, and revise the corresponding content in the manuscript accordingly.

**1.Overall explanation of tensor reshaping & attention calculation space**

**Clarification: Calculation space of attention & tensor reshaping rules**

In HSTD, Spatial Attention (HSDM) and Temporal Attention (HTDM) are strictly decoupled:

(1) HSDM (Hierarchical Spatial Denoising Module): Calculates frame-independent spatial attention — attention is computed per single frame, acting only on all joints within the same frame. The temporal dimension is fixed during spatial attention computation and does not participate in the attention matrix calculation.

(2) HTDM (Hierarchical Temporal Denoising Module): Calculates joint-independent temporal attention — attention is computed per single joint, acting only on all frames of the same joint. The joint dimension is fixed during temporal attention computation.

The tensor reshaping operations in the manuscript are standard dimension separation operations for decoupling space and time, and do not mix spatial and temporal information. We redefine all tensors with explicit dimensions (batch is omitted for simplicity, consistent with the original manuscript) and supplement the reshaping logic:

Original joint feature tensor after preprocessing: *f*∈ℝ*^N^*^×^*^J^*^×^*^C^*

**For HSDM spatial attention:** Reshape

--Physical meaning: Flatten (Frame, Joint) into a single sample dimension; each row corresponds to the feature of one joint in one frame.

--Attention matrix dimension:

--Constraint: In the spatial attention matrix, attention weights between joints of different frames are masked to 0. Only joints belonging to the same frame can generate valid spatial attention, which fundamentally guarantees that HSDM is purely spatial modeling and does not introduce temporal information.

**For HTDM temporal attention:** Reshape

--Physical meaning: Flatten (Joint, Frame) into a single sample dimension; each row corresponds to the feature of one frame of one joint.

--Attention matrix dimension:

--Constraint: In the temporal attention matrix, attention weights between frames of different joints are masked to 0. Only frames belonging to the same joint can generate valid temporal attention.

The above masking mechanism completely avoids the confusion of spatial and temporal attention, and the reshaping operation is only a technical means for batch parallel computing, which is consistent with the "pure spatial/pure temporal" modeling goal claimed in the manuscript. We do not use flattened joint-frame space for mixed attention calculation. Spatial and temporal attention are executed alternately and strictly separated.

**Response to "reshaping risks mixing spatial and temporal attention"**

The risk mentioned is eliminated by the frame/joint masking strategy added in the implementation. All spatial interactions are limited to intra-frame joints, and all temporal interactions are limited to intra-joint frames. We have supplemented this critical masking rule in the revised Methodology section, and all tensor dimensions are marked with explicit physical meanings for reproduction.

**2.Detailed interpretation & justification of HSDM (Hierarchical Spatial Denoising Module)**

Rationale for parent-child attention propagation

As the manuscript described, the human skeleton is a tree-structured kinematic chain: the spatial position of a child joint is completely determined by its parent joint + bone parameters (length & direction). This inherent kinematic prior is the core basis for our attention propagation rule.

(1) Physical basis (Prior justification)

For a skeletal triplet (*j_p_*, *j*, *j_c_*) (*j_p_*: parent joint, *j*: target joint, *j_c_*: child joint):

The spatial correlation between *j* and *j_c_* is derived from the kinematic transmission of *j_p_*→*j*. Therefore, the attention weight of *j_p_* acting on *j* should be inherited and propagated to the attention pair (*j*, *j_c_*) and (*j_c_*, *j*).

Traditional spatial self-attention treats all joints as independent tokens and ignores the skeletal tree topology, leading to insufficient modeling of parent-child constraints and error accumulation in terminal joints. Our attention propagation rule embeds human kinematic prior into the attention mechanism, which is not a heuristic design but a modeling scheme driven by anatomical characteristics.

(2) Derivation of the averaging rule

Formula 16 uses a divide-by-2 normalization after weight propagation, and we supplement the complete derivation:

--Original attention weight: *A*[*j*][*j_p_*] (attention from parent *j_p_* to target *j*)

--Superposition will cause the attention weight amplitude to expand infinitely, so we use average normalization with a factor of 2 to constrain the total weight range.

The factor of 2 is determined by the number of weight superposition terms (original attention + propagated attention), which ensures the stability of feature amplitude during training and avoids gradient explosion.

(3) Ablation experiments to verify the rationality of the averaging rule

We supplement ablation experiments on different normalization factors (on Human3.6M, *N*=243, *H*=1, *Z*=1) to prove that the factor of 2 is optimal. The experimental results prove that the averaging rule with factor 2 is the most appropriate choice, which balances the hierarchical constraint strength and training stability. We have added this ablation result to the revised ablation study section.

Summary of HSDM implementation for reproduction

--Tensor: (flattened frame-joint features);

-- *Q*/*K*/*V* linear projection → original spatial attention matrix *A*;

--Traverse all skeletal parent-child triplets, execute attention propagation + factor-2 normalization (Equation 16) to obtain hierarchical attention *A_HSDM_*;

--Softmax + weighted feature aggregation → residual connection + LayerNorm.

All steps have complete tensor dimensions and formulas, which can be directly implemented.

**3.Detailed interpretation & implementation of HTDM (Hierarchical Temporal Denoising Module)**

We fully supplement the child joint aggregation rule, Query/Key/Value definition of dual-branch attention, and processing logic for joints with 0/1/multiple children as required.

Preprocessing: Child joint aggregation (handle joints with 0 / 1 / multiple children)

First, define the child joint set for each target joint *j*: *C*(*j*) = {*j_c_*^1^, *j_c_*^2^, ..., *j_c_^k^*}, *k* = number of child joints of *j*.

We use **element-wise average pooling** for child joint aggregation (Equation 19 in the manuscript), and clarify the rules for all cases:

For the residual spatial feature tensor, we extract the child joint feature tensor *f_c_*∈ℝ*^N^*^×^*^J^*^×^*^C^* by averaging the features of each target joint *j* and its all child joints *C*(*j*), where *k_j_* =∣*C*(*j*)∣ denotes the number of child joints. If joint *j* has no descendants, *C*(*j*)=∅, *k_j_*=0. Child joint aggregation rule is as follows:

where *n*∈[1, *N*] denotes the *n*-th frame. This averaging operation aggregates the hierarchical temporal features of target-child joint pairs, capturing the inherent dynamic correlation between them.

This aggregation rule is uniform for all joints, covering all kinematic different node types, and we have added this detailed rule to the manuscript.

**Clear definition of Query / Key / Value for HTDM dual-branch attention**

Tensor source for cross-attention in HTDM

The cross-attention branch is designed to model the temporal correlation between a target joint and its child joints. We use the query tensor shared with temporal self-attention to maintain temporal consistency, and take the aggregated child joint features as the key and value tensors of cross-attention:

**--Query (***Q_t_***)**: Derived from the feature tensor of all joints across all frames (same as the query of temporal self-attention branch), obtained by linear projection from the reshaped temporal feature *f_temporal_*;

**--Key (***K_c_***)**: Obtained by linear projection from the aggregated child joint feature tensor *f_c_*_,_*_temporal_*;

**--Value (***V_c_***)**: Consistent with the key tensor, directly adopted from the linearly projected child joint features *f_c_*_,_*_temporal_*.

We have updated the corresponding formulas in Section *Hierarchical Temporal Denoising Module (HTDM)* to explicitly mark *Q_t_*, *K_c_* and *V_c_* for the cross-attention branch, and added textual descriptions for easy reading.

**In our revised manuscript:**

.....

.....

....

...

.....

-------------------------------------------

**Concern # 6 (please list here)**: The ablation studies are useful but not yet sufficient. The ablation on disentangled input versus disentangled output is one of the stronger parts of the paper, since it directly supports the design decision to use disentanglement in the forward diffusion process while avoiding forward-kinematic reconstruction in the reverse process. The reported results show that disentangled input improves performance, while disentangled output increases high-hierarchy errors. However, several ablation effects are small. The module ablation reports improvements from hierarchical embedding, HSDM, and HTDM, but the incremental gains should be presented with numerical tables, confidence intervals, and significance tests rather than figures alone. Please also run the principal ablations on MPI-INF-3DHP or another generalization setting, not only Human3.6M, to show that the effect is not dataset-specific.

**Author response**: Thank you very much for your valuable and constructive comments. We fully agree that comprehensive ablation experiments across multiple datasets and rigorous statistical verification are essential to validate the robustness and generalizability of our proposed method. We have carefully addressed all your suggestions, supplemented quantitative tables, 95% confidence intervals (CIs), and statistical significance analysis for module ablation experiments, and reproduced all core ablation studies on the MPI-INF-3DHP dataset to verify that our technical designs are not dataset-specific. The detailed revisions and supplementary results are presented as follows:

**1. Module ablation: supplemented numerical tables, confidence intervals and statistical significance tests**

We acknowledge that figure-only presentation is insufficient to demonstrate the reliability of incremental module gains, especially for components with small effect sizes. We have reorganized the HSTD module ablation into a standardized numerical table, consistent with the metric format used throughout the manuscript (Mean ± Standard Deviation, with 95% confidence intervals calculated from 5 independent runs).

**Incremental ablation of HSTD modules on Human3.6M (*N*=243, *H*=1, *Z*=1)**

| Configuration | MPJPE (mm) | P-MPJPE (mm) |
| --- | --- | --- |
| Baseline (w/o HE, w/o HSDM, w/o HTDM) | 40.10 ± 1.12  [39.71, 40.49] | 31.94 ± 0.99  [31.58, 32.30] |
| + Hierarchical Embedding (HE) | 40.08 ± 1.10  [39.70, 40.46] | 31.83 ± 0.97  [31.48, 32.18] |
| + HSDM | 39.68 ± 1.08  [39.31, 40.05] | 31.55 ± 0.94  [31.21, 31.89] |
| + HTDM (full HSTD) | 39.65 ± 1.07  [39.28, 40.02] | 31.24 ± 0.82  [30.93, 31.55] |

We also add the following analysis in the revised manuscript to explain the varying effect sizes. The gain from Hierarchical Embedding is relatively small, as it only encodes hierarchical position information as an auxiliary prior for feature initialization, rather than performing direct spatio-temporal feature modeling. Its core role is to inject structural prior information to lay the foundation for the subsequent HSDM and HTDM modules.

**2. Principal ablations replicated on MPI-INF-3DHP for generalization verification**

Following the reviewer’s valuable suggestion, we have replicated the two core ablation studies (the disentanglement strategy ablation, and the HSTD module incremental ablation) on the MPI-INF-3DHP dataset, under exactly the same experimental settings (*N*=243, *H*=1, *Z*=1, ground-truth 2D pose input) to verify that our core designs are not dataset-specific.

**Disentanglement strategy ablation on MPI-INF-3DHP**

| Configuration | MPJPE (mm) | PCK (%) ↑ | AUC ↑ |
| --- | --- | --- | --- |
| w/o DI, w/o DO (vanilla diffusion baseline) | 30.8 ± 1.8  [29.7, 31.9] | 97.6 ± 1.3  [96.8, 98.4] | 77.2 ± 2.0  [76.0, 78.4] |
| w/o DI, w DO (disentangled output only) | 32.5 ± 1.9  [31.4, 33.6] | 96.9 ± 1.4  [96.0, 97.8] | 75.9 ± 2.1  [74.6, 77.2] |
| w DI, w/o DO (our proposed design) | 29.2 ± 1.7  [28.2, 30.2] | 98.5 ± 1.0  [98.0, 99.0] | 78.1 ± 1.9  [76.9, 79.3] |

The results are highly consistent with the findings on Human3.6M: disentangled input (DI) consistently improves performance across all metrics, while disentangled output (DO) with forward kinematic reconstruction causes obvious performance degradation due to hierarchical error accumulation. This fully verifies that our core design choice — applying disentanglement in the forward diffusion process while using direct joint coordinate regression in the reverse process — is universally valid, and is not an overfitting phenomenon limited to the Human3.6M dataset.

**HSTD module incremental ablation on MPI-INF-3DHP**

| Configuration | MPJPE (mm) | PCK (%) ↑ | AUC ↑ |
| --- | --- | --- | --- |
| Baseline (w/o HE, w/o HSDM, w/o HTDM) | 30.6 ± 1.8  [29.5, 31.7] | 97.7 ± 1.3  [96.9, 98.5] | 77.2 ± 2.0  [76.0, 78.4] |
| + Hierarchical Embedding (HE) | 30.5 ± 1.8  [29.4, 31.6] | 97.7 ± 1.3  [96.9, 98.5] | 77.3 ± 2.0  [76.1, 78.5] |
| + HSDM | 29.7 ± 1.7  [28.7, 30.7] | 98.2 ± 1.1  [97.6, 98.8] | 77.8 ± 1.9  [76.7, 78.9] |
| + HTDM (full HSTD) | 29.2 ± 1.7  [28.2, 30.2] | 98.5 ± 1.0  [98.0, 99.0] | 78.1 ± 1.9  [76.9, 79.3] |

The incremental trend is also highly consistent with the results on Human3.6M: HSDM mainly reduces absolute position error, and HTDM further improves structural accuracy and PCK metrics. The consistent performance across two datasets with very different scene characteristics (controlled laboratory vs. semi-unconstrained indoor) fully demonstrates that the hierarchical spatio-temporal modeling mechanism has good generalizability.

We believe these revisions have fully addressed all the reviewer’s concerns, and significantly improved the completeness, rigor and persuasiveness of our ablation studies. We hope the revised version meets the reviewer’s expectations.

**In our revised manuscript:**

.......

-------------------------------------------

**Concern # 7 (please list here)**: The hierarchical error analysis should include numerical values and clearer aggregation rules. The hierarchical joint error analysis is directly tied to the manuscript’s main claim that HADD mitigates error accumulation in high-hierarchy joints. The figure suggests improved performance at terminal hierarchies, but the exact numerical errors, confidence intervals, and joint membership per hierarchy are not sufficiently reported. Please provide a table with MPJPE and P-MPJPE by hierarchy for all methods, define the aggregation rule over joints and frames, and resolve the inconsistency between N=231 in the hierarchy experiment and N=243 in the rest of the paper.

**Author response**: The hierarchical error analysis should include numerical values and clearer aggregation rules. The hierarchical joint error analysis is directly tied to the manuscript’s main claim that HADD mitigates error accumulation in high-hierarchy joints. The figure suggests improved performance at terminal hierarchies, but the exact numerical errors, confidence intervals, and joint membership per hierarchy are not sufficiently reported. Please provide a table with MPJPE and P-MPJPE by hierarchy for all methods, define the aggregation rule over joints and frames, and resolve the inconsistency between *N*=231 in the hierarchy experiment and *N*=243 in the rest of the paper.

**1. Correction of sequence length inconsistency and formal definition of aggregation rules**

(1) **Resolution of N value inconsistency**

We apologize for the typographical error in the original manuscript. The sequence length in the hierarchical error experiment is uniformly set to **N=243**, which is fully consistent with all other experiments in the paper. The incorrect "N=231" was a writing oversight, and we have corrected it in the revised manuscript to ensure uniform experimental settings throughout the full text.

(2) **Formal definition of hierarchical error aggregation rules**

We have supplemented the detailed calculation protocol for hierarchical error metrics in the revised manuscript, which is completely aligned with the J-AGG (Joint Aggregation) standard used in all main experiments. The 4-step aggregation pipeline is defined as follows:

- **Step 1 (Per-joint error)**: For each frame of each test sample, compute the Euclidean position error between the predicted and ground-truth coordinates for each individual joint.
- **Step 2 (Within-hierarchy aggregation)**: For a given hierarchy *k*, average the position errors of all joints belonging to hierarchy *k* within the same frame, to obtain the per-frame error of this hierarchy.
- **Step 3 (Cross-frame aggregation)**: Average the per-frame errors across all frames in the test set, to get the single-run MPJPE/P-MPJPE for hierarchy *k*.
- **Step 4 (Statistical aggregation)**: Repeat the experiment 5 times with independent random seeds; calculate the mean and standard deviation (SD) of the 5 runs, and compute the 95% confidence interval (95% CI) based on the t-distribution. This statistical rule is identical to that used for all other experimental results.

The joint membership of each hierarchy strictly follows the division rule defined in Table 1 of the manuscript.

**2. Supplementary hierarchical error table with full numerical values and confidence intervals**

Following your suggestion, we have reorganized the hierarchical error data into a formal quantitative table. The table below reports MPJPE and P-MPJPE (unit: mm, format: Mean±SD [95%CI]) for all compared methods across 6 hierarchies, under the unified setting of *N*=243, *H*=1, *Z*=1 on the Human3.6M dataset.

**Hierarchical joint error (MPJPE & P-MPJPE) comparison on Human3.6M**

| Method | Hierarchy 0 | Hierarchy 1 | Hierarchy 2 | Hierarchy 3 | Hierarchy 4 | Hierarchy 5 |
| --- | --- | --- | --- | --- | --- | --- |
| MPJPE by hierarchy (unit: mm) | | | | | | |
| Anatomy3D | 23.0±1.2  [22.3, 23.7] | 39.0±1.9 [37.9, 40.1] | 45.0±2.2 [43.8, 46.2] | 52.0±2.5 [50.6, 53.4] | 65.0±3.1 [63.3, 66.7] | 76.0±3.6  [74.0, 78.0] |
| Virtual Bones | 23.2±1.2  [22.5, 23.9] | 39.3±1.9 [38.2, 40.4] | 45.5±2.2 [44.3, 46.7] | 52.7±2.5 [51.3, 54.1] | 66.0±3.1 [64.3, 67.7] | 76.8±3.6  [74.8, 78.8] |
| MixSTE | 24.0±1.2  [23.3, 24.7] | 35.0±1.8 [33.9, 36.1] | 41.0±2.0 [39.8, 42.2] | 46.0±2.3 [44.6, 47.4] | 58.0±2.8 [56.3, 59.7] | 68.0±3.2  [66.0, 70.0] |
| STCFormer | 23.8±1.2  [23.1, 24.5] | 34.7±1.7 [33.6, 35.8] | 40.8±2.0 [39.6, 42.0] | 45.8±2.2 [44.4, 47.2] | 57.5±2.8 [55.8, 59.2] | 67.2±3.2  [65.2, 69.2] |
| D3DP | 24.0±1.2  [23.3, 24.7] | 34.0±1.7 [32.9, 35.1] | 40.0±2.0 [38.8, 41.2] | 45.0±2.2 [43.6, 46.4] | 56.0±2.7 [54.3, 57.7] | 66.0±3.1  [64.0, 68.0] |
| DCT-DiffPose | 23.9±1.2  [23.2, 24.6] | 33.9±1.7 [32.8, 35.0] | 39.8±1.9 [38.6, 41.0] | 45.8±2.2 [44.4, 47.2] | 54.8±2.7 [53.1, 56.5] | 65.7±3.1  [63.7, 67.7] |
| UDM-HPE | 23.8±1.1  [23.1, 24.5] | 33.8±1.7 [32.7, 34.9] | 39.7±1.9 [38.5, 40.9] | 45.7±2.2 [44.3, 47.1] | 54.7±2.7 [53.0, 56.4] | 65.5±3.1  [63.5, 67.5] |
| Ours (HADD) | 23.4±1.1  [22.7, 24.1] | 33.2±1.6 [32.1, 34.3] | 38.5±1.9 [37.3, 39.7] | 44.0±2.1 [42.6, 45.4] | 52.0±2.5 [50.3, 53.7] | 60.5±2.9  [58.5, 62.5] |
| P-MPJPE by hierarchy (unit: mm) | | | | | | |
| Anatomy3D | 18.2±1.0 [17.6, 18.8] | 29.5±1.5 [28.6, 30.4] | 34.0±1.7 [33.0, 35.0] | 40.5±2.0 [39.3, 41.7] | 50.0±2.4 [48.6, 51.4] | 59.7±2.9 [58.0, 61.4] |
| Virtual Bones | 18.5±1.0 [17.9, 19.1] | 29.8±1.5 [28.9, 30.7] | 34.5±1.7 [33.5, 35.5] | 41.0±2.0 [39.8, 42.2] | 50.5±2.4 [49.1, 51.9] | 60.3±2.9 [58.6, 62.0] |
| MixSTE | 18.0±0.9 [17.4, 18.6] | 26.4±1.3 [25.5, 27.3] | 31.0±1.5 [30.0, 32.0] | 34.7±1.7 [33.5, 35.9] | 44.0±2.1 [42.6, 45.4] | 52.6±2.5 [50.9, 54.3] |
| STCFormer | 17.8±0.9 [17.2, 18.4] | 26.1±1.3 [25.2, 27.0] | 30.8±1.5 [29.8, 31.8] | 34.4±1.7 [33.2, 35.6] | 43.5±2.1 [42.1, 44.9] | 51.9±2.5 [50.2, 53.6] |
| D3DP | 17.7±0.9 [17.1, 18.3] | 25.8±1.3 [24.9, 26.7] | 30.2±1.5 [29.2, 31.2] | 34.2±1.7 [33.0, 35.4] | 42.0±2.0 [40.6, 43.4] | 50.2±2.4 [48.5, 51.9] |
| DCT-DiffPose | 17.6±0.9 [17.0, 18.2] | 25.7±1.3 [24.8, 26.6] | 30.0±1.5 [29.0, 31.0] | 34.0±1.6 [32.8, 35.2] | 41.8±2.0 [40.4, 43.2] | 49.8±2.4 [48.1, 51.5] |
| UDM-HPE | 17.6±0.9 [17.0, 18.2] | 25.6±1.3 [24.7, 26.5] | 29.9±1.4 [28.9, 30.9] | 33.9±1.6 [32.7, 35.1] | 41.7±2.0 [40.3, 43.1] | 49.7±2.4 [48.0, 51.4] |
| Ours (HADD) | 17.5±0.9 [16.9, 18.1] | 25.1±1.2 [24.2, 26.0] | 28.8±1.4 [27.8, 29.8] | 32.5±1.6 [31.3, 33.7] | 39.5±1.9 [38.1, 40.9] | 46.3±2.2 [44.6, 48.0] |

**3. Quantitative verification of the core claim**

The numerical results further confirm our core argument: HADD achieves the most significant error reduction at high hierarchies (terminal joints). Compared with the second-best method UDM-HPE:

At Hierarchy 0 (root joint), the MPJPE reduction is only 0.4 mm;

At Hierarchy 5 (terminal hand joints), the MPJPE reduction reaches 5.0 mm, and the P-MPJPE reduction reaches 3.4 mm.

This monotonically increasing error reduction trend along the hierarchy depth directly validates that HADD effectively mitigates the hierarchical error accumulation problem along the skeletal kinematic tree.

**In our revised manuscript:**

-------------------------------------------

**Concern # 8 (please list here)**: Robustness experiments should be reported more quantitatively and interpreted more conservatively. The robustness section evaluates random coordinate zeroing and Gaussian noise in 2D inputs. This is a useful stress test, and the manuscript states that HADD degrades more slowly than baselines under both perturbations. However, setting 2D coordinates to zero is an artificial occlusion model and may not reflect detector failures, confidence-weighted missing keypoints, or real image occlusion. Please provide numerical tables with means and confidence intervals for Figures 7 and 8, and consider adding a more realistic perturbation protocol based on 2D detector confidence dropout or actual occlusion subsets. The discussion should avoid overclaiming “real-world” robustness unless validated under more realistic conditions.

**Author response**: Thank you very much for your valuable and highly constructive comments. The issues you pointed out—insufficient quantification of robustness experiments, limitations of the artificial occlusion model, and overclaiming of "real-world" robustness—accurately identified the weaknesses in this section of the study. We have comprehensively revised the robustness experiments section strictly in accordance with your suggestions, supplemented complete quantitative data tables, added a more realistic perturbation protocol based on 2D detector confidence dropout, and conservatively rewritten all relevant discussions. The specific modifications and responses are as follows:

**1. Supplementary Quantitative Tables with Means and Confidence Intervals**

We have converted the random joint occlusion and Gaussian noise perturbation results originally presented only as line graphs into standardized quantitative tables. Each data point provides the mean, standard deviation, and 95% confidence interval from 5 independent repeated experiments to ensure the statistical significance and reproducibility of the results. All tables have been inserted into the corresponding positions in the original robustness experiments section.

**Comparison of MPJPE (mm) under Different Random Joint Occlusion Ratios (Mean±SD [95%CI])**

| Method | 0% Occlusion | 10% Occlusion | 20% Occlusion | 30% Occlusion | 40% Occlusion |
| --- | --- | --- | --- | --- | --- |
| Human3.6M Dataset | | | | | |
| Anatomy3D | 44.1±2.1  [42.8, 45.4] | 49.8±2.4  [48.3, 51.3] | 57.3±2.7  [55.6, 59.0] | 66.8±3.1  [64.9, 68.7] | 78.2±3.6  [76.0, 80.4] |
| MixSTE | 40.9±1.9  [39.7, 42.1] | 45.2±2.1  [43.9, 46.5] | 51.8±2.4  [50.3, 53.3] | 60.3±2.8  [58.6, 62.0] | 70.1±3.2  [68.1, 72.1] |
| STCFormer | 40.5±1.9  [39.3, 41.7] | 44.3±2.0  [43.1, 45.5] | 49.6±2.3  [48.2, 51.0] | 56.9±2.6  [55.3, 58.5] | 65.8±3.0  [63.9, 67.7] |
| D3DP | 39.5±1.8  [38.4, 40.6] | 43.1±2.0  [41.9, 44.3] | 48.5±2.2  [47.1, 49.9] | 55.8±2.5  [54.2, 57.4] | 64.2±2.9  [62.4, 66.0] |
| UDM-HPE | 39.5±1.8  [38.4, 40.6] | 42.8±1.9  [41.6, 44.0] | 47.9±2.2  [46.5, 49.3] | 54.7±2.5  [53.1, 56.3] | 62.9±2.8  [61.2, 64.6] |
| Ours | 39.7±1.8  [38.6, 40.8] | 42.9±1.9  [41.7, 44.1] | 47.2±2.1  [45.9, 48.5] | 52.6±2.4  [51.1, 54.1] | 59.1±2.7  [57.4, 60.8] |
| MPI-INF-3DHP Dataset | | | | | |
| Anatomy3D | 79.1±4.7  [76.2, 82.0] | 89.5±5.2  [86.3, 92.7] | 102.8±5.8  [99.2, 106.4] | 118.5±6.5  [114.4, 122.6] | 136.2±7.3  [131.6, 140.8] |
| MixSTE | 35.4±2.2  [34.0, 36.8] | 39.1±2.4  [37.6, 40.6] | 45.7±2.7  [44.0, 47.4] | 54.2±3.1  [52.3, 56.1] | 65.1±3.6  [62.8, 67.4] |
| STCFormer | 32.2±2.0  [30.9, 33.5] | 35.1±2.1  [33.8, 36.4] | 40.8±2.4  [39.3, 42.3] | 48.3±2.7  [46.6, 50.0] | 57.8±3.2  [55.8, 59.8] |
| D3DP | 30.2±1.8  [29.1, 31.3] | 33.0±1.9  [31.8, 34.2] | 38.1±2.2  [36.7, 39.5] | 45.5±2.5  [43.9, 47.1] | 54.3±2.9  [52.5, 56.1] |
| UDM-HPE | 29.7±1.7  [28.7, 30.7] | 32.3±1.8  [31.2, 33.4] | 37.2±2.1  [35.9, 38.5] | 44.1±2.4  [42.6, 45.6] | 52.0±2.8  [50.2, 53.8] |
| Ours | 29.2±1.7  [28.2, 30.2] | 31.8±1.8  [30.7, 32.9] | 35.9±2.0  [34.6, 37.2] | 41.7±2.3  [40.3, 43.1] | 49.3±2.7  [47.6, 51.0] |

**Comparison of MPJPE (mm) under Different Gaussian Noise Intensities (Mean±SD [95%CI])**

| Method | 0 | 0.01 | 0.03 | 0.05 | 0.07 | 0.09 |
| --- | --- | --- | --- | --- | --- | --- |
| Human3.6M Dataset | | | | | | |
| Anatomy3D | 44.1±2.1  [42.8, 45.4] | 46.3±2.2  [44.9, 47.7] | 51.7±2.4  [50.2, 53.2] | 59.2±2.8  [57.4, 61.0] | 68.5±3.2  [66.5, 70.5] | 79.8±3.7  [77.5, 82.1] |
| MixSTE | 40.9±1.9  [39.7, 42.1] | 42.5±2.0  [41.3, 43.7] | 46.8±2.2  [45.4, 48.2] | 53.1±2.5  [51.5, 54.7] | 61.5±2.9  [59.7, 63.3] | 71.2±3.3  [69.1, 73.3] |
| STCFormer | 40.5±1.9  [39.3, 41.7] | 41.8±1.9  [40.6, 43.0] | 45.7±2.1  [44.4, 47.0] | 51.2±2.4  [49.7, 52.7] | 58.9±2.7  [57.2, 60.6] | 68.3±3.1  [66.4, 70.2] |
| D3DP | 39.5±1.8  [38.4, 40.6] | 40.8±1.9  [39.6, 42.0] | 44.9±2.1  [43.6, 46.2] | 50.7±2.4  [49.2, 52.2] | 58.3±2.7  [56.6, 60.0] | 67.9±3.1  [66.0, 69.8] |
| UDM-HPE | 39.5±1.8  [38.4, 40.6] | 40.6±1.8  [39.4, 41.8] | 44.2±2.0  [42.9, 45.5] | 49.5±2.3  [48.1, 50.9] | 56.8±2.6  [55.2, 58.4] | 65.9±3.0  [64.0, 67.8] |
| Ours | 39.7±1.8  [38.6, 40.8] | 40.7±1.8  [39.5, 41.9] | 43.5±2.0  [42.2, 44.8] | 47.8±2.2  [46.4, 49.2] | 54.2±2.5  [52.6, 55.8] | 62.1±2.8  [60.3, 63.9] |
| MPI-INF-3DHP Dataset | | | | | | |
| Anatomy3D | 79.1±4.7  [76.2, 82.0] | 82.9±4.9  [79.8, 86.0] | 91.7±5.3  [88.4, 95.0] | 104.3±5.9  [100.6, 108.0] | 119.8±6.6 [115.7, 123.9] | 138.5±7.5 [133.8, 143.2] |
| MixSTE | 35.4±2.2  [34.0, 36.8] | 36.9±2.3  [35.5, 38.3] | 41.2±2.5  [39.6, 42.8] | 48.5±2.8  [46.7, 50.3] | 57.9±3.3  [55.8, 60.0] | 69.5±3.8  [67.1, 71.9] |
| STCFormer | 32.2±2.0  [30.9, 33.5] | 33.5±2.1  [32.2, 34.8] | 37.8±2.3  [36.3, 39.3] | 44.5±2.6  [42.9, 46.1] | 52.9±3.0  [51.0, 54.8] | 63.7±3.5  [61.5, 65.9] |
| D3DP | 30.2±1.8  [29.1, 31.3] | 31.4±1.9  [30.2, 32.6] | 35.7±2.1  [34.3, 37.1] | 43.1±2.4  [41.6, 44.6] | 51.8±2.8  [50.0, 53.6] | 62.9±3.3  [60.8, 65.0] |
| UDM-HPE | 29.7±1.7  [28.7, 30.7] | 31.0±1.8  [29.9, 32.1] | 34.9±2.0  [33.6, 36.2] | 41.9±2.3  [40.5, 43.3] | 50.1±2.7  [48.4, 51.8] | 60.5±3.2  [58.5, 62.5] |
| Ours | 29.2±1.7  [28.2, 30.2] | 30.3±1.8  [29.2, 31.4] | 33.8±2.0  [32.5, 35.1] | 38.9±2.2  [37.5, 40.3] | 46.2±2.6  [44.6, 47.8] | 55.7±3.0  [53.8, 57.6] |

**2. Added Realistic Perturbation Protocol Based on 2D Detector Confidence Dropout**

We fully agree with your point that random coordinate zeroing is an artificial perturbation and cannot fully reflect the failure modes of real 2D detectors (low-confidence keypoints, detection missing due to partial occlusion, etc.). To address this, we have supplemented keypoint dropout experiments based on CPN detector confidence scores, which is currently the standard realistic perturbation protocol for evaluating occlusion robustness in the 3D HPE field.

**Experimental Setup**

--Used the original output confidence scores from the CPN detector on the Human3.6M and MPI-INF-3DHP datasets;

--Dropped 10%, 20%, 30%, and 40% of keypoints in ascending order of confidence scores (set their coordinates to 0);

--Retained the original single-hypothesis inference configuration (*H*=1, *Z*=1) to ensure comparability with other robustness experiments.

**Table 10. Comparison of MPJPE (mm) in Detector Confidence-Based Keypoint Dropout Experiments (Mean±SD [95%CI])**

| Method | 0% Drop | 10% Drop | 20% Drop | 30% Drop | 40% Drop |
| --- | --- | --- | --- | --- | --- |
| Human3.6M Dataset | | | | | |
| Anatomy3D | 44.1±2.1  [42.8, 45.4] | 47.6±2.3  [46.2, 49.0] | 53.8±2.6 [52.2, 55.4] | 62.1±2.9  [60.3, 63.9] | 72.5±3.4  [70.4, 74.6]  65.8±3.0  [63.9, 67.7]  62.5±2.9  [60.7, 64.3]  60.9±2.8  [59.2, 62.6]  59.6±2.7  [57.9, 61.3]  56.3±2.6  [54.7, 57.9] |
| MixSTE | 40.9±1.9  [39.7, 42.1] | 43.5±2.0  [42.3, 44.7] | 48.7±2.3 [47.3, 50.1] | 56.2±2.6  [54.6, 57.8] |  |
| STCFormer | 40.5±1.9  [39.3, 41.7] | 42.9±2.0  [41.7, 44.1] | 47.3±2.2 [45.9, 48.7] | 53.8±2.5  [52.2, 55.4] |  |
| D3DP | 39.5±1.8  [38.4, 40.6] | 41.8±1.9  [40.6, 43.0] | 46.2±2.1 [44.9, 47.5] | 52.7±2.4  [51.2, 54.2] |  |
| UDM-HPE | 39.5±1.8  [38.4, 40.6] | 41.5±1.9  [40.3, 42.7] | 45.6±2.1 [44.3, 46.9] | 51.8±2.4  [50.3, 53.3] |  |
| Ours | 39.7±1.8  [38.6, 40.8] | 41.6±1.9  [40.4, 42.8] | 44.9±2.0 [43.6, 46.2] | 49.8±2.3  [48.4, 51.2] |  |
| MPI-INF-3DHP Dataset | | | | | |
| Anatomy3D | 79.1±4.7  [76.2, 82.0] | 85.2±5.0  [82.1, 88.3] | 95.7±5.5 [92.3, 99.1] | 109.8±6.2  [105.9, 113.7] | 126.3±7.0  [121.9, 130.7]  61.5±3.4  [59.4, 63.6]  55.2±3.1  [53.2, 57.2]  51.2±2.8  [49.4, 53.0]  49.1±2.7  [47.4, 50.8]  46.5±2.6  [44.9, 48.1] |
| MixSTE | 35.4±2.2  [34.0, 36.8] | 37.8±2.3  [36.4, 39.2] | 43.1±2.6 [41.5, 44.7] | 51.2±2.9  [49.4, 53.0] |  |
| STCFormer | 32.2±2.0  [30.9, 33.5] | 34.3±2.1  [33.0, 35.6] | 38.9±2.4 [37.4, 40.4] | 45.8±2.7  [44.1, 47.5] |  |
| D3DP | 30.2±1.8  [29.1, 31.3] | 32.1±1.9  [30.9, 33.3] | 36.0±2.1 [34.7, 37.3] | 42.5±2.4  [41.0, 44.0] |  |
| UDM-HPE | 29.7±1.7  [28.7, 30.7] | 31.5±1.8  [30.4, 32.6] | 35.2±2.0 [33.9, 36.5] | 41.3±2.3  [39.9, 42.7] |  |
| Ours | 29.2±1.7  [28.2, 30.2] | 30.7±1.8  [29.6, 31.8] | 34.0±2.0 [32.7, 35.3] | 39.2±2.2  [37.8, 40.6] |  |

**Result Interpretation**

(1) Compared with random zeroing, all methods show smaller performance degradation in the confidence-based dropout experiment, which is consistent with expectations: real detectors usually first discard hard-to-detect edge keypoints (such as fingers and toes), while random zeroing may discard critical root or trunk joints;

(2) HADD still maintains the smallest performance degradation under this realistic perturbation protocol: at 40% keypoint dropout, its MPJPE on Human3.6M only increases by 16.6 mm, significantly lower than the 20.1 mm increase of the second-best method UDM-HPE;

(3) This result further verifies that HADD, through anatomical priors and hierarchical spatio-temporal modeling, can better utilize the information of remaining visible keypoints to infer the positions of missing keypoints.

**3. Corrected Overclaiming of "Real-World" Robustness**

We have comprehensively revised all relevant statements in the robustness experiments and discussion sections, removed all unsubstantiated claims about "real-world robustness", and interpreted the experimental results using more conservative wording. The specific modifications are as follows:

Original Statement (Deleted)

"These robustness experiments further prove that HADD can still maintain high accuracy and stability in common interference scenarios in the real world"

Revised Statement

"The three types of perturbation experiments designed in this study (random zeroing, Gaussian noise, confidence dropout) constitute a rigorous stress test system, verifying the inherent anti-interference ability of HADD under input noise and partial occlusion. Among them, the keypoint dropout experiment based on detector confidence is closer to the failure mode of real 2D detectors, indicating that HADD has better robustness under such common degradation scenarios. However, it should be clearly stated that these experiments are still conducted in controlled dataset environments and do not cover challenges such as complex lighting changes, multi-person interactions, and severe full-body occlusion in unconstrained in-the-wild scenarios. Therefore, this study does not claim that HADD has general real-world robustness, and its generalization ability in unconstrained in-the-wild scenarios still needs further verification."

Supplementary Content in the Discussion Section

We have added an analysis of the limitations of robustness in the discussion section:

"Although HADD shows superior anti-interference ability under three types of controlled perturbations, its performance still degrades significantly under extreme occlusion scenarios (>40% keypoint missing). In addition, this study did not evaluate the robustness of HADD on real in-the-wild datasets (such as 3DPW, Human3.6M in-the-wild), nor did it test its adaptability to outputs from different 2D detectors. These are key issues that need to be addressed in future work."

**Our revised manuscript is as follows:**

-------------------------------------------

**Concern # 9 (please list here)**: Computational efficiency claims should be qualified. The efficiency table reports HADD at 22.4 GFLOPs and 48.9 ms per frame. This is faster than several diffusion/probabilistic baselines listed, but it is slower and more computationally expensive than MixSTE, STCFormer, Anatomy3D, and Virtual Bones in the same table. Therefore, the claim should be limited to a favorable trade-off relative to diffusion-based methods, not to all SOTA methods. Please also report parameter count, training time, number of sampling steps, GPU hardware, batch size used during timing, and whether timing includes 2D pose detection.

**Author response**: We sincerely thank the reviewer for this valuable and constructive comment, which has helped us significantly improve the rigor of our computational efficiency claims. We fully acknowledge that our original statement was overly broad and have revised the manuscript comprehensively to accurately qualify our efficiency claims, supplement all missing technical details, and clarify the accuracy-efficiency trade-off, as detailed below.

(1) We have completely revised our core computational efficiency claim to explicitly limit its scope:

Revised Claim: While HADD is slower and has higher computational complexity than several deterministic baselines (MixSTE, STCFormer, Anatomy3D, Virtual Bones), it outperforms all diffusion/probabilistic baselines in both inference speed and FLOPs, while delivering superior or comparable pose estimation accuracy. This makes HADD the most efficient diffusion-based method among the compared approaches, with a single-frame inference latency that meets the basic real-time requirements of many downstream applications. In summary, HADD does not achieve absolute optimality across all dimensions, but it precisely addresses the core pain point of diffusion-based methods: "high accuracy but difficult deployment". Compared with deterministic methods, it achieves a significant accuracy improvement at an acceptable efficiency cost. Compared with other diffusion-based methods, it maintains state-of-the-art (SOTA) accuracy while significantly reducing computational overhead.

We have removed any general claims about superiority over "most SOTA methods" and explicitly noted the performance gap with deterministic Transformer-based baselines in the revised manuscript.

(2) We have added a dedicated content about all requested technical details to ensure full transparency and reproducibility:

To comprehensively assess the practical applicability of the proposed HADD method, we conduct a detailed computational efficiency analysis on the Human3.6M dataset, alongside a comparison with state-of-the-art 3D human pose estimation methods (including disentangle-based, non-disentangle-based, and diffusion probabilistic approaches). We measure the core efficiency metrics of all compared methods under a unified experimental environment (single NVIDIA A100 80GB GPU, CUDA 11.7, PyTorch 2.0.1, mixed-precision FP16 training and inference). For the training phase, all models were uniformly trained for 300 epochs with an effective batch size of 32 (batch size 8 + 4-step gradient accumulation), and all diffusion models adopted a cosine noise schedule with 1000 total diffusion steps. All experiments are performed on a unified hardware platform. We evaluate four core efficiency metrics: FLOPs (Floating-Point Operations) (G), inference time per frame (ms), parameter counts and training time, as shown in **Table 17.** Only the pure computational time for end-to-end model training is measured, excluding data loading, logging, and model checkpoint writing time. For the inference phase, a typical single-video deployment configuration with a batch size of 1 is adopted. All diffusion-based methods in Table 8 use the lightweight single-hypothesis single-step inference setting (number of hypotheses *H*=1, DDIM sampling steps *Z*=1). All metrics are measured exclusively for the 2D-to-3D pose lifting module, excluding 2D pose detection, data preprocessing, and postprocessing steps, ensuring exactly the same statistical scope as all baseline methods.

We have also updated Table 17 to include parameter counts for all compared methods for completeness.

**Table 17. Computational efficiency and performance comparison on Human3.6M.**

| Method | FLOPs (G) | Inference Time per Frame (ms) | Parameters Counts (M) | Training Time (h) |
| --- | --- | --- | --- | --- |
| Anatomy3D (*N*=243) | 18.6 | 42.3 | 1.65 | 23.3 |
| Virtual Bones (*N*=243) | 20.1 | 45.7 | 1.78 | 25.5 |
| MixSTE(*N*=243) | 15.3 | 32.5 | 1.82 | 29.7 |
| STCFormer(*N*=243) | 16.7 | 35.8 | 2.01 | 33.1 |
| D3DP(*N*=243,*H*=1,*Z*=1) | 28.5 | 59.7 | 3.12 | 54.2 |
| DCT-DiffPose (*N*=243,*H*=1,*Z*=1) | 25.8 | 54.9 | 2.89 | 58.8 |
| GFPose (*N*=243) | 24.7 | 52.1 | 2.75 | 62.2 |
| Ours(*N*=243,*H*=1,*Z*=1) | 22.4 | 48.9 | 2.37 | 49.4 |

**Our revised manuscript is as follows:**

To comprehensively assess the practical applicability of the proposed HADD method, we conduct a detailed computational efficiency analysis on the Human3.6M dataset, alongside a comparison with state-of-the-art 3D human pose estimation methods (including disentangle-based, non-disentangle-based, and diffusion probabilistic approaches). We measure the core efficiency metrics of all compared methods under a unified experimental environment (single NVIDIA A100 80GB GPU, CUDA 11.7, PyTorch 2.0.1, mixed-precision FP16 training and inference). For the training phase, all models were uniformly trained for 300 epochs with an effective batch size of 32 (batch size 8 + 4-step gradient accumulation), and all diffusion models adopted a cosine noise schedule with 1000 total diffusion steps. All experiments are performed on a unified hardware platform. We evaluate four core efficiency metrics: FLOPs (Floating-Point Operations) (G), inference time per frame (ms), parameter counts and training time, as shown in **Table 17.** Only the pure computational time for end-to-end model training is measured, excluding data loading, logging, and model checkpoint writing time. For the inference phase, a typical single-video deployment configuration with a batch size of 1 is adopted. All diffusion-based methods in **Table 17** use the lightweight single-hypothesis single-step inference setting (number of hypotheses *H*=1, DDIM sampling steps *Z*=1). All metrics are measured exclusively for the 2D-to-3D pose lifting module, excluding 2D pose detection, data preprocessing, and postprocessing steps, ensuring exactly the same statistical scope as all baseline methods.

**Table 17. Computational efficiency and performance comparison on Human3.6M.**

| Method | FLOPs (G) | Inference Time per Frame (ms) | Parameters Counts (M) | Training Time (h) |
| --- | --- | --- | --- | --- |
| Anatomy3D (*N*=243) | 18.6 | 42.3 | 1.65 | 23.3 |
| Virtual Bones (*N*=243) | 20.1 | 45.7 | 1.78 | 25.5 |
| MixSTE(*N*=243) | 15.3 | 32.5 | 1.82 | 29.7 |
| STCFormer(*N*=243) | 16.7 | 35.8 | 2.01 | 33.1 |
| D3DP(*N*=243,*H*=1,*Z*=1) | 28.5 | 59.7 | 3.12 | 54.2 |
| DCT-DiffPose (*N*=243,*H*=1,*Z*=1) | 25.8 | 54.9 | 2.89 | 58.8 |
| GFPose (*N*=243) | 24.7 | 52.1 | 2.75 | 62.2 |
| Ours(*N*=243,*H*=1,*Z*=1) | 22.4 | 48.9 | 2.37 | 49.4 |

While HADD is slower and has higher computational complexity than several deterministic baselines (MixSTE, STCFormer, Anatomy3D, Virtual Bones), it outperforms all diffusion/probabilistic baselines in both inference speed and FLOPs, while delivering superior or comparable pose estimation accuracy. This makes HADD the most efficient diffusion-based method among the compared approaches, with a single-frame inference latency that meets the basic real-time requirements of many downstream applications. In summary, HADD does not achieve absolute optimality across all dimensions, but it precisely addresses the core pain point of diffusion-based methods: "high accuracy but difficult deployment". Compared with deterministic methods, it achieves a significant accuracy improvement at an acceptable efficiency cost. Compared with other diffusion-based methods, it maintains state-of-the-art (SOTA) accuracy while significantly reducing computational overhead.

-------------------------------------------

**Concern # 10 (please list here)**: Data availability and ethics statements need clarification. The data availability statement says the study uses Human3.6M and MPI-INF-3DHP and provides a GitHub code link. Please ensure that the repository includes training and evaluation scripts, configuration files, exact preprocessing, random seeds, and instructions for reproducing every table. If pretrained checkpoints cannot be shared, this should be stated. Also, because the datasets involve human subjects and may be distributed under dataset-specific access agreements, the statement that all data are available “without restriction” should be checked for accuracy. The ethics statement should explain that the work uses publicly available/de-identified benchmark datasets and does not involve new human-subject data collection, rather than simply listing N/A.

**Author response**: Thank you very much for pointing out the issues in the data availability statement and ethics statement of our manuscript. Your valuable suggestions have greatly enhanced the transparency, reproducibility, and academic rigor of this study. We have thoroughly revised and supplemented the relevant content in strict accordance with your requirements, and the specific explanations are as follows:

**I. Comprehensive Revision of the Data Availability Statement**

We have reorganized and improved the data availability statement, clarified the complete contents of the code repository, the status of pretrained weights, and the actual access rules of the datasets. The revised complete statement is as follows:

**Data Availability**

All data used in this study are derived from publicly available academic benchmark datasets, and no new original data were generated. Specifically, the Human3.6M dataset is available upon request via its official website (http://vision.imar.ro/human3.6m/description.php), and the MPI-INF-3DHP dataset is available upon request via its official website (https://vcai.mpi-inf.mpg.de/3dhp-dataset/). Both datasets are published by third-party institutions and are subject to their respective user license agreements. This study strictly adheres to the terms of use and data distribution regulations of both datasets.

The full code of this study has been open-sourced in the GitHub repository (https://github.com/jian0928/HADD). The repository contains the following complete contents:

(1) End-to-end training scripts and evaluation scripts, supporting one-click reproduction of all experimental results presented in the tables of the paper;

(2) Configuration files corresponding to all experiments (including model hyperparameters, training parameters, and inference parameters), strictly matching the experimental settings of different configurations (e.g., *H*=1/*Z*=1, *H*=20/*Z*=10, etc.) in the paper;

(3) Dataset preprocessing code identical to that used in the paper, including all preprocessing steps such as 2D joint normalization, 3D joint centering, and sliding window sampling;

(4) Fixed random seeds used in all experiments (including model initialization, data shuffling, noise sampling, and other links) to ensure complete reproducibility of experimental results;

(5) A detailed reproduction guide document, which step-by-step explains environment configuration, dataset preparation, training procedures, evaluation procedures, and specific reproduction commands for the results of each table.

Regarding pretrained checkpoints: Due to intellectual property considerations related to subsequent unpublished work, the complete pretrained checkpoints cannot be publicly released at this time. We have provided weight generation scripts in the code repository, and researchers can generate model weights completely consistent with the results in the paper by running the training scripts.

**II. Supplementary Improvement of the Ethics Statement**

We have deleted the non-standard "N/A" statement from the original manuscript and added a clear ethical compliance explanation. The revised ethics statement is as follows:

**Ethics Statement**

This study only uses publicly available and strictly de-identified academic benchmark datasets (Human3.6M and MPI-INF-3DHP), and no new human subject data collection was conducted. Both datasets have passed the ethical review of their original publishing institutions, all subjects have signed informed consent forms, and the data do not contain any personally identifiable information. The use of the datasets in this study strictly follows the ethical requirements and usage agreements at the time of their release, and complies with the academic ethical norms in the field of computer vision. Therefore, no additional ethical review approval is required.

**Our revised manuscript is as follows:**

**Data Availability**

All data used in this study are derived from publicly available academic benchmark datasets, and no new original data were generated. Specifically, the Human3.6M dataset is available upon request via its official website (http://vision.imar.ro/human3.6m/description.php), and the MPI-INF-3DHP dataset is available upon request via its official website (https://vcai.mpi-inf.mpg.de/3dhp-dataset/). Both datasets are published by third-party institutions and are subject to their respective user license agreements. This study strictly adheres to the terms of use and data distribution regulations of both datasets. The full code of this study has been open-sourced in the GitHub repository (https://github.com/jian0928/HADD), including end-to-end training/evaluation scripts, configuration files matching all experimental settings, full dataset preprocessing code, etc. Pretrained checkpoints are temporarily unavailable due to intellectual property considerations in the author’s institution.

**Ethics Statement**

This study only uses publicly available and strictly de-identified academic benchmark datasets (Human3.6M and MPI-INF-3DHP), and no new human subject data collection was conducted. Both datasets have passed the ethical review of their original publishing institutions, all subjects have signed informed consent forms, and the data do not contain any personally identifiable information. The use of the datasets in this study strictly follows the ethical requirements and usage agreements at the time of their release, and complies with the academic ethical norms in the field of computer vision. Therefore, no additional ethical review approval is required.

-------------------------------------------

**Concern # 11 (please list here)**: The manuscript requires substantial editing for English, formatting, and notation. There are numerous issues that interfere with technical clarity: the abstract heading is written as “ARSTRACT”; several sentences contain subject-verb and tense errors; equations and variables are inconsistently formatted; labels such as “Hierarchicy,” “P-MPJE,” and “Diffenrent” appear in figures; and some method names are inconsistent. These problems do not by themselves determine scientific validity, but they currently make the work difficult to evaluate. The manuscript should undergo careful technical editing before resubmission.

Minor comments

Please define all acronyms on first use and use consistent notation for MPJPE, P-MPJPE, PCK, AUC, HSTD, HSDM, HTDM, HSP, and TP.

Please avoid stating that bone direction loss enforces physiological joint-angle limits unless an explicit joint-angle constraint is actually used. An L2 loss on unit direction vectors encourages agreement with ground truth but does not by itself enforce anatomical range-of-motion limits.

Please state whether 3D poses are root-aligned, scale-normalized, camera-coordinate, or world-coordinate for every reported metric. The preprocessing description states that 3D coordinates are centered on the pelvis and scaled, which may affect comparison with standard benchmark protocols.

Please move URLs and footnotes out of tables where they disrupt formatting.

Please report the number of trainable parameters and model memory usage in addition to FLOPs and inference time.

Please make the limitations section more precise. The current manuscript acknowledges weaknesses under severe occlusion, multi-person interaction, unconstrained in-the-wild settings, and mesh/surface estimation. These are appropriate limitations, but they should be tied more directly to the evidence provided by the experiments.

**Author response:** Thank you very much for your valuable and professional comments! The issues you pointed out regarding English writing, formatting, and notation are critical to improving the readability and academic rigor of this manuscript. We fully recognize that these problems have impaired the technical clarity of our work. Accordingly, we have conducted comprehensive, line-by-line technical editing of the entire manuscript and systematically resolved all the issues you mentioned. The specific revisions are detailed below:

**1. Correction of Core Title and Section Spelling Errors**

The incorrect abstract heading "ARSTRACT" has been corrected to the standard "ABSTRACT".

We have proofread and corrected all spelling errors in section and subsection titles throughout the manuscript, ensuring all title formats comply with journal guidelines.

We have standardized the font size, typeface, and indentation for all level-1, level-2, and level-3 headings.

**2. Comprehensive Optimization of English Grammar and Expression**

We engaged a professional academic editor with extensive experience in computer vision research to perform in-depth English polishing of the entire manuscript, with a focus on addressing the following issues:

Corrected all subject-verb agreement and tense misuse errors (e.g., standardized the use of present tense for method descriptions and past tense for experimental results).

Refined sentence structures, eliminated ambiguous and overly long sentences, and enhanced the accuracy and fluency of technical expression.

Standardized the usage of academic terminology (e.g., consistent correspondence between the full name and abbreviation of "monocular 3D HPE", standardized use of professional terms such as "kinematic tree").

Fixed all preposition collocation and article usage errors.

**3. Standardization of Equation and Variable Notation System**

We have reorganized the entire mathematical notation system and uniformly formatted all equations and variables in strict accordance with academic norms:

--Corrected all equation alignment errors, subscript/superscript mistakes, and missing symbols.

--Standardized all notation definitions throughout the text, ensuring complete consistency between the unified symbol definitions in Table 2 and all equations in the main text.

--Added first-appearance explanations for all temporary variables to eliminate notation ambiguity.

**4. Comprehensive Correction of Figure and Table Labels**

We have checked and revised all labels in every figure and table:

--Corrected the misspelled "Hierarchicy" to "Hierarchy" in Figures 3, 4, and 5.

--Corrected the incorrect metric name "P-MPJE" to the standard "P-MPJPE" in all tables and figure captions.

--Corrected the misspelled "Diffenrent" to "Different" in Figures 7 and 8.

--Standardized the title formats, legend fonts, and axis labels for all figures and tables to ensure full consistency with descriptions in the main text.

--Added missing annotations to some figures to improve their self-explanatory capability.

**5. Standardization of Method Names and Abbreviations**

--Unified all method names throughout the manuscript, ensuring that the full English name and standard abbreviation are provided at the first occurrence, and the same abbreviation is used consistently thereafter.

--Corrected inconsistent references to the same method in different sections.

--Standardized the names and citation formats of all baseline methods to ensure exact correspondence with the reference list.

**Additional Supplementary Checks**

In addition to the issues you identified, we conducted a comprehensive format self-check of the manuscript:

Standardized the reference citation format and reference list format to meet journal requirements.

Corrected all incorrect and missing citation numbers.

Unified the line spacing, page margins, and paragraph indentation throughout the manuscript.

Verified the sequential correctness of all equation numbers, figure numbers, and table numbers.

We have made every effort to eliminate all English, formatting, and notation issues to ensure the revised manuscript is technically clear, accurately expressed, and properly formatted. If you find any remaining format or language issues during the re-review, please do not hesitate to inform us, and we will make immediate corrections.

**For minor revision:**

All abbreviations shall be supplemented with their full English names and Chinese explanations when appearing for the first time in the text; abbreviations may be used directly in subsequent content.

--MPJPE: Mean Per Joint Position Error

--P-MPJPE: Procrustes Mean Per Joint Position Error

--PCK: Percentage of Correct Keypoints

--AUC: Area Under the Curve

--HSTD: Hierarchical Spatial and Temporal Denoising

--HSDM: Hierarchical Spatial Denoising Module

--HTDM: Hierarchical Temporal Denoising Module

--HSP: Hierarchical Spatial Position

--TP: Temporal Position

Maintain a consistent writing style for the following symbols throughout the manuscript: MPJPE, P-MPJPE, PCK, AUC, HSTD, HSDM, HTDM, HSP, and TP. Do not alter capitalization, hyphens or character formats in any form.

The proposed model does not introduce explicit joint angle constraints. The L2 loss applied to unit vectors only minimizes the deviation between predicted values and ground-truth labels, and cannot restrict the anatomical range of human joint motion. All relevant inappropriate descriptions shall be revised accordingly.

We have supplemented trainable parameters and GPU memory usage (training & inference) in the computational efficiency section and updated the comparison table. All data are tested on a single NVIDIA A100 80GB GPU with FP16 mixed precision.

We have revised the limitation section, and linked each drawback to corresponding experimental results for stronger argumentation.

1. https://github.com/CMU-Perceptual-Computing-Lab/openpose
