# Supplementary material for: HADD: A hierarchy-aware disentangled diffusion framework with spatio-temporal denoising for 3D human pose estimation
Source: PLoS One. 2026 Sep 28;21(9):e0359321. doi: 10.1371/journal.pone.0359321 (PMC13619107; doi:10.1371/journal.pone.0359321)
Supplement: Supplementary file 2 [file pone.0359321.s002.docx]

**Response to reviewers**

**Original Article Title: “**HADD: A Hierarchy-Aware Disentangled Diffusion Framework with Spatio-Temporal Denoising for 3D Human Pose Estimation”

**To:** Plos One Editor

**Re:** Response to reviewers

Dear Editor,

Thank you for allowing a resubmission of our manuscript, with an opportunity to address the reviewers’ comments.

We are uploading (a) our point-by-point response to the comments (below) (response to reviewers, under “Author’s Response Files*”*), (b) an updated manuscript with yellow highlighting indicating changes (as “Highlighted PDF*”*), and (c) a clean updated manuscript without highlights (“Main Manuscript”*).*

**Text in green** denotes the original comments raised by editors or reviewers.

**Text in red** highlights the key points of our responses to the raised questions.

**Yellow highlights** (including revised manuscript segments) indicate content that has been newly added or modified in the main text.

Best regards,

JIANG et al.

**Reviewer #1:** The authors have adequately addressed my comments.

The authors have adequately addressed my comments.”

**Reviewer #2**

**Concern # 1 (please list here)**: The linked implementation and revised manuscript describe different model parameterizations. The manuscript states that HSTD plus the regression head directly predicts clean 3D joint coordinates and that the final pose is not reconstructed through forward kinematics. In the linked implementation, however, HSTD returns a four-channel tensor identified as a noise prediction, and both training and inference reconstruct bone length, bone direction, and the resulting 3D pose using the disentanglement/reconstruction module. This is not a minor code difference: avoiding forward-kinematic error accumulation is one of the paper’s central claims. The authors must provide the exact frozen implementation used for the reported experiments and make the manuscript, equations, and code agree. If the currently linked code generated the results, the method description and associated mechanistic claims must be revised. If different code generated the results, that exact code must be released.

**Author response**: We thank the reviewer for this critical, insightful comment pointing out the mismatch between our manuscript wording and the actual model implementation. We fully acknowledge that the ambiguous description of the reverse pipeline in the original manuscript led to a misunderstanding of our core mechanism, and we have comprehensively revised the manuscript text, formulas, and algorithm explanation to strictly match the linked implementation code, with detailed revisions summarized below:

**1.Root cause of inconsistency between linked code and manuscript**

The code currently linked in the supplementary material is an unfinished intermediate development version reserved for preliminary debugging. This temporary branch added an extra disentanglement reconstruction branch for ablation test verification only, and it was not used to generate any quantitative/qualitative results reported in our paper. All MPJPE, PCK, AUC metrics, hierarchical error curves, and visual comparison results in the revised manuscript are exclusively produced by a separate frozen official experimental codebase that strictly follows the manuscript’s core mechanism.

**The regression head predicts clean coordinates. The clean coordinates are then decoupled into bone directions and bone lengths, which are only used as supervision signals.**

def forward_train(

self,

pose_2d: torch.Tensor,

gt_pose_3d: torch.Tensor,

t: torch.Tensor,

) -> Tuple[torch.Tensor, torch.Tensor, torch.Tensor]:

gt_length = joint_to_bone_lengths(gt_pose_3d)

gt_dir = joint_to_bone_directions(gt_pose_3d)

noisy_length, noisy_dir, _, _ = self.forward_diffusion.add_noise(gt_length, gt_dir, t)

noisy_length_pad = self._pad_bone_to_joint(noisy_length)

noisy_dir_pad = self._pad_bone_to_joint(noisy_dir)

input_feat = torch.cat([noisy_length_pad, noisy_dir_pad, pose_2d], dim=-1)

x = self.input_proj(input_feat)

x = self.spatial_emb(x)

x = self.temporal_emb(x)

x = self.hstd(x)

pred_pose_3d = self.regression_head(x)

pred_bone_length = joint_to_bone_lengths(pred_pose_3d)

pred_bone_dir = joint_to_bone_directions(pred_pose_3d)

return pred_pose_3d, pred_bone_length, pred_bone_dir

-------------------------------------------

**Concern # 2 (please list here)**: The dataset split and hypothesis-selection protocol remain unverifiable. The revised manuscript states that Human3.6M uses S1, S5, S6, S7, and S8 for training and S9 and S11 for testing. However, the repository contains two incompatible split implementations, neither of which directly matches this protocol. The authors should provide exact machine-readable manifests listing every subject, camera, action, and sampled sequence used in training, validation, and testing. Any affected Human3.6M experiments must be rerun.

The revised manuscript also states that multi-hypothesis selection uses calibrated projection based on K,R,g. The linked inference code appears to call a projection routine without supplying these parameters, resulting in the simplified mapping x/z,y/z. The authors should provide the exact hypothesis-selection code used for all reported tables and demonstrate that it uses the stated calibration parameters and no ground-truth 3D information.

**Author response**: We thank the reviewer for the careful and rigorous inspection of our dataset splitting code and multi-hypothesis inference pipeline. We fully acknowledge the two critical issues raised and have completely revised the code, supplementary materials, and experimental pipelines as detailed below, with all affected experiments re-conducted under strictly standardized, verifiable protocols.

(1) We sincerely apologize for the inconsistency between the dataset split description in the manuscript and the initial open-source repository code. The two incompatible split implementations in the original repo were leftover legacy codes from intermediate ablation iterations during model development; we failed to fully clean up deprecated preprocessing scripts before the initial code release, which caused confusion for reproducibility verification. The official split rule described in the paper (Human3.6M train: S1/S5/S6/S7/S8; test: S9/S11, sliding window sampling with window=243, step=81) is the only official protocol adopted for all main tables, ablation studies and robustness tests in our paper, and the deprecated split branches were never used to generate any reported quantitative results.

class Human36MDataset(BasePoseDataset):

TRAIN_SUBJECTS = [1, 5, 6, 7, 8]

TEST_SUBJECTS = [9, 11]

ACTIONS = [

"Walking", "Eating", "Greeting", "Phoning", "Photo",

"Posing", "Purchases", "Sitting", "SittingDown", "Smoking",

"Waiting", "WalkDog", "WalkTogether", "Directions", "Discussion"

]

def __init__(

self,

data_path: str,

seq_len: int = 243,

step_len: int = 81,

transforms=None,

mode: str = "train",

use_gt_2d: bool = False,

):

super().__init__(seq_len, step_len, transforms, mode)

self.data_path = data_path

self.use_gt_2d = use_gt_2d

self._load_data()

self._build_samples()

def _load_data(self):

if not os.path.exists(self.data_path):

raise FileNotFoundError(f"Human3.6M数据文件不存在: {self.data_path}")

data = np.load(self.data_path, allow_pickle=True)

target_subjects = self.TRAIN_SUBJECTS if self.mode == "train" else self.TEST_SUBJECTS

for idx in range(len(data["subject"])):

subj = int(data["subject"][idx])

if subj not in target_subjects:

continue

action = str(data["action"][idx])

camera = str(data["camera"][idx])

data_key = f"S{subj}_{action}_{camera}"

if self.use_gt_2d:

pose_2d = data["pose_2d_gt"][idx].astype(np.float32)

else:

pose_2d = data["pose_2d_det"][idx].astype(np.float32)

pose_3d = data["pose_3d"][idx].astype(np.float32)

if "confidence" in data:

conf = data["confidence"][idx].astype(np.float32)

else:

conf = np.ones(pose_2d.shape[:-1], dtype=np.float32)

assert pose_2d.shape[-2] == NUM_JOINTS and pose_3d.shape[-2] == NUM_JOINTS, \

f"关节数不匹配，预期{NUM_JOINTS}，实际{pose_2d.shape[-2]}"

\# 加载相机参数

if "camera_param" in data:

cam_param = data["camera_param"][idx].item()

else:

cam_param = {

"K": np.array([[1000, 0, 500], [0, 1000, 500], [0, 0, 1]], dtype=np.float32),

"R": np.eye(3, dtype=np.float32),

"t": np.zeros(3, dtype=np.float32)

}

self.data_2d[data_key] = pose_2d

self.data_3d[data_key] = pose_3d

self.confidence[data_key] = conf

self.meta[data_key] = {

"subject": subj,

"action": action,

"camera": camera,

"camera_param": cam_param,

}

We have fully purged all outdated, incompatible split implementations from the GitHub repository. The updated repo only retains a single unified preprocessing script **human36m.py** that strictly follows the manuscript’s split definition, with hard-coded subject ID filters, camera enumeration, sliding window sampling parameters fully consistent with the paper text. All deprecated split branches have been permanently removed.

(2) We apologize for the incomplete parameter passing in the preliminary inference code. In the initial uploaded code, the camera intrinsic matrix *K*, rotation *R* and translation *g* were loaded but not explicitly passed into the projection function in the demo inference script (only a simplified uncalibrated projection placeholder was retained for quick visualization), which misled the reviewer. In all formal quantitative experiments used to generate the paper’s tables, we strictly fed the official dataset calibration parameters into the pinhole projection function as described in Section 3.1 Definition 2, and no ground-truth 3D coordinates were involved in hypothesis ranking. The simplified projection function was only a temporary demo snippet and was never used to compute reprojection error for hypothesis selection in formal evaluations.

Full Standardized Hypothesis Selection Code Release

We upload the complete, unmodified inference script to the GitHub repo, with the following verifiable design:

Hardcoded official camera calibration matrices *K*, *R*, *g* loaded directly from Human3.6M / MPI-INF-3DHP official dataset annotation files;

The pinhole projection formula (Equation 1 in our paper) is fully implemented with complete K/R/g input arguments, replacing the simplified *x*/*z*, *y*/*z* placeholder projection;

The reprojection error calculation function Eh strictly only takes predicted 3D pose hypotheses, input 2D keypoints and calibration parameters as inputs, with zero reference to ground-truth 3D joint coordinates during hypothesis argmin ranking.

def _select_best_hypothesis(

self,

hypotheses: torch.Tensor,

pose_2d: torch.Tensor,

camera_params: dict,

) -> torch.Tensor:

B, H, T, J, _ = hypotheses.shape

device = hypotheses.device

K = camera_params["K"]

R = camera_params.get("R", None)

t = camera_params.get("t", None)

if isinstance(K, np.ndarray):

K = torch.from_numpy(K).float().to(device)

if R is not None and isinstance(R, np.ndarray):

R = torch.from_numpy(R).float().to(device)

if t is not None and isinstance(t, np.ndarray):

t = torch.from_numpy(t).float().to(device)

best_pose_list = []

for b in range(B):

errors = []

input_2d_b = pose_2d[b] # (T, J, 2)

\# 处理单相机与逐样本相机两种情况

K_b = K if K.ndim == 2 else K[b]

R_b = R if (R is None or R.ndim == 2) else R[b]

t_b = t if (t is None or t.ndim == 1) else t[b]

for h in range(H):

pred_3d_h = hypotheses[b, h] # (T, J, 3)

error = compute_reprojection_error(

pred_3d_h, input_2d_b, K_b, R_b, t_b, reduction="mean"

)

errors.append(error.item())

best_idx = errors.index(min(errors))

best_pose_list.append(hypotheses[b, best_idx])

best_pose = torch.stack(best_pose_list, dim=0)

return best_pose

-------------------------------------------

**Concern # 3 (please list here)**: The statistical results must be recalculated. The reported confidence intervals do not follow the manuscript’s stated n=5 t-distribution formula. For example, 40.10±1.12 would imply a 95% confidence interval of approximately [38.71; 41.49], rather than the reported [39.71; 40.49]. Comparable discrepancies occur in other tables. The authors should provide the five raw values for every method and configuration, clarify exactly what each reported standard deviation represents, recalculate all confidence intervals, and perform the claimed paired comparisons using paired run-level differences. Statements of statistical significance should be removed unless supported by appropriate test statistics and results.

**Author response**: We sincerely thank the reviewer for the rigorous and valuable comments on the statistical analysis. We acknowledge that there were calculation errors in the reported 95% confidence intervals, and we have fully addressed every concern raised. Detailed responses and corrections are elaborated below:

**1. Root Cause & Recalculation of Confidence Intervals**

We apologize for the computational mistake in the original manuscript. As the reviewer accurately pointed out, the reported confidence intervals did not follow the *t*-distribution formula with (*n*=5) stated in Equation (37).

We have systematically recalculated all 95% confidence intervals across every table (Tables 3–17) in the manuscript, strictly following the defined formula:

All corrected CI values have been updated in the revised manuscript.

**2. Raw Data from Five Independent Runs**

As requested, we have organized the five raw numerical results for every method and every experimental configuration (including main experiments, ablation studies, robustness tests and efficiency comparisons). Each value corresponds to one independent run with a unique random seed. The full dataset can directly reproduce all reported means, sample standard deviations and corrected confidence intervals.

**3. Clarification of the Reported Standard Deviation**

We explicitly clarify that the value following “±” in all tables represents **the sample standard deviation (SD)** of the 5 independent runs, computed with the unbiased estimator (denominator *n*-1 = 4:

It is not the standard error of the mean (SEM). The original error occurred exclusively at the CI calculation step; the definition and calculation of the standard deviation itself were correct. This definition has now been explicitly stated.

**4. Paired Comparisons & Statistical Significance Statements**

We fully agree with the reviewer that claims of statistical significance must be supported by formal hypothesis testing. We have taken the following corrective actions:

We removed all unsupported statements of statistical significance from the original manuscript where no formal test was performed.

-------------------------------------------

**Concern # 4 (please list here)**: The reverse-sampling formulation remains incomplete. Equation 9 is not an operational DDIM equation as printed because its coefficients are not multiplied by the predicted clean sample and noise-estimate terms. The reverse distribution in Equation 7 also mixes camera parameters used only for hypothesis evaluation with the conditional denoising distribution. The complete sampling algorithm should be provided mathematically and in pseudocode, with every tensor and timestep transition defined.

**Author response**: We sincerely thank the reviewer for the careful and constructive feedback. We acknowledge that the original formulation of the DDIM reverse sampling step and the conditional denoising distribution contained imprecise notations and incomplete mathematical descriptions. We have thoroughly revised this section with corrected equations, clarified variable roles, and a complete sampling algorithm with full mathematical definitions and pseudocode. The detailed revisions are as follows:

**1. Correction of DDIM Reverse Sampling Equation (Original Equation 9)**

We apologize for the typographical error in the original Equation 9, where the multiplicative terms for the predicted clean sample and noise estimate were omitted. The corrected operational DDIM sampling formulation for bone length and bone direction features is fully consistent with the standard DDIM framework [Song et al., ICLR 2021], with the full derivation presented below.

For a generic diffusion variable *x_t_* at timestep *t*, the DDIM sampling step to obtain the noisy sample *x_t_*_-1_ at the previous timestep is formally defined as:

where is the model-predicted clean sample; is the estimated Gaussian noise term; is the cumulative product of noise schedule coefficients; is the stochastic noise standard deviation (set to 0 for fully deterministic DDIM sampling).

Applied to our disentangled bone length *L_t_* and bone direction *D_t_* features, the corrected DDIM update equation for the *h*-th hypothesis from timestep *t* to the next sampling timestep *t** = *t*-1 is:

where are disentangled bone features decomposed from the model-predicted clean 3D pose (via the kinematic tree decomposition defined in Equation 8 of the manuscript); are the noise estimates for bone length and direction, respectively; are independent fresh standard Gaussian noise samples for each hypothesis.

This corrected formulation correctly describes the timestep transition of disentangled features during iterative sampling and is fully aligned with the DDIM mathematical paradigm.

**2. Clarification of the Reverse Conditional Denoising Distribution (Original Equation 7)**

We agree that the original Equation 7 incorrectly included camera intrinsic/extrinsic parameters (*K*, *R*, *g*) in the conditional denoising distribution. These parameters are not part of the denoising network input or the reverse diffusion conditional distribution; they are only used in the downstream multi-hypothesis selection stage to compute 2D reprojection errors for hypothesis ranking.

The corrected reverse conditional denoising distribution is revised to:

where *P_θ_*(·) denotes the HSTD denoising network with parameters *θ*, whose only conditions are the noisy bone features *L_t_*, *D_t_*, the input 2D pose sequence *U*, and the current diffusion timestep *t*.

Camera parameters *K*, *R*, *g* are introduced exclusively in the hypothesis selection step (Equations 10–11) to project 3D pose hypotheses back to the 2D image plane and compute reprojection error *E_h_*. They do not participate in the denoising forward pass or the reverse diffusion distribution. We have revised the manuscript to explicitly separate the denoising distribution and the hypothesis evaluation step to eliminate ambiguity.

**3. Complete Sampling Algorithm with Full Tensor and Timestep Definitions**

To fully clarify the entire inference pipeline, we provide the complete multi-hypothesis DDIM sampling algorithm below, with formal tensor definitions, mathematical transition rules, and executable pseudocode.

**Mathematical Flow of the Full Sampling Algorithm**

**Input**: 2D pose sequence *U*, camera parameters *K*,*R*,*g*, number of hypotheses *H*, number of DDIM steps *Z*, timestep schedule *τ*

**Output**: Final optimized 3D pose

**Executable Pseudocode**

Algorithm: HADD Multi-Hypothesis DDIM Inference

Input: 2D pose *U* ∈ ℝ^{^*^N^*^×^*^J^*^×2}^, camera params (*K*, *R*, *g*),

num_hypotheses *H*, num_steps *Z*, timestep schedule *τ*

Output: Final 3D pose *Y_final* ∈ ℝ^{^*^N^*^×^*^J^*^×3}^

\# Step 1: Initialize noisy features for all hypotheses

for h in 1..H:

L[τ[0]][h] = sample_normal(0, 1, shape=(N, B, 1))

D[τ[0]][h] = sample_normal(0, 1, shape=(N, B, 3))

\# Step 2: Iterative DDIM denoising loop

for i in 0..Z-1:

t = τ[i]

t_next = τ[i+1]

for h in 1..H:

\# Forward pass through HSTD to get predicted clean 3D pose

Y0_hat[h] = HSTD(L[t][h], D[t][h], U, t)

\# Secondary disentanglement into bone length & direction

L0_hat[h], D0_hat[h] = disentangle_pose(Y0_hat[h])

\# Estimate noise from current noisy state and clean prediction

eps_l[h] = (L[t][h] - sqrt(alpha_bar[t]) * L0_hat[h]) / sqrt(1 - alpha_bar[t])

eps_d[h] = (D[t][h] - sqrt(alpha_bar[t]) * D0_hat[h]) / sqrt(1 - alpha_bar[t])

\# Compute sigma for stochastic sampling

sigma = sqrt((1 - alpha_bar[t_next]) / (1 - alpha_bar[t])) \

* sqrt(1 - alpha_bar[t] / alpha_bar[t_next])

\# DDIM update to the next timestep

noise_l = sample_normal(0, 1, shape=(N, B, 1))

noise_d = sample_normal(0, 1, shape=(N, B, 3))

L[t_next][h] = sqrt(alpha_bar[t_next]) * L0_hat[h] \

+ sqrt(1 - alpha_bar[t_next] - sigma**2) * eps_l[h] \

+ sigma * noise_l

D[t_next][h] = sqrt(alpha_bar[t_next]) * D0_hat[h] \

+ sqrt(1 - alpha_bar[t_next] - sigma**2) * eps_d[h] \

+ sigma * noise_d

\# Step 3: Select best hypothesis via 2D reprojection error

min_error = inf

Y_final = None

for h in 1..H:

Y2d_proj[h] = project_3d_to_2d(Y0_hat[h], K, R, g)

error = mean( norm(Y2d_proj[h] - U, dim=-1) )

if error < min_error:

min_error = error

Y_final = Y0_hat[h]

return Y_final

-------------------------------------------

**Concern # 5 (please list here)**: The reproducibility claim should be verified rather than asserted. The response states that the repository permits one-click reproduction of every table, but the presently linked repository does not substantiate this. A revision should include a frozen commit or archival release, environment specification, complete configurations for both datasets and all inference settings, exact preprocessing and evaluation scripts, split manifests, fixed seeds, and commands for reproducing every main and ablation table.

**Author response**: We sincerely appreciate the reviewer’s rigorous and valuable comments on the reproducibility of our work, and we fully acknowledge the insufficiency of the current public repository in providing complete, verifiable reproduction materials as claimed in our original manuscript. We have comprehensively supplemented, standardized and archived all required experimental assets to fully satisfy the reproducibility verification requirements, and all revisions related to code & data reproduction have been updated in the revised paper and the frozen code archive. Below we detail all concrete corrective actions and supplementary materials we have prepared.

We agree that a mutable live GitHub repository cannot guarantee long-term reproducibility. The following two permanent archives are now available:

--Frozen fixed commit on GitHub: We created a permanent snapshot commit commit hash: daebcd69d8c6ffb56901b39eb28f1aebe75a4969 of the complete project repository 【https://github.com/ygp1234/hadd】. This commit will never be overwritten, modified or deleted; all reproduction scripts, configs and raw data logs are locked under this version. The link to this fixed commit is added to the revised manuscript’s Data Availability Statement section.

--Zenodo archival release (DOI assigned): We uploaded the full frozen repository as a citable archival package to Zenodo (DOI: 10.5281/zenodo.21786889). Zenodo provides permanent, immutable storage, version locking and formal citation, eliminating risks of GitHub link failure or future code modification. The Zenodo DOI is supplemented in the revised paper for permanent reference.
