# Supplementary material for: HADD: A hierarchy-aware disentangled diffusion framework with spatio-temporal denoising for 3D human pose estimation
Source: PLoS One. 2026 Sep 28;21(9):e0359321. doi: 10.1371/journal.pone.0359321 (PMC13619107; doi:10.1371/journal.pone.0359321)
Supplement: Peer review history

# Peer review: Decision Letter 0

**DOI:** 10.1371/journal.pone.0359321.r001

**Authors:** Gennady S. Cymbalyuk

4 Jun 2026

Dear Dr. Jian,

Thank you for submitting your manuscript to PLOS ONE. After careful consideration, we feel that it has merit but does not fully meet PLOS ONE’s publication criteria as it currently stands. Therefore, we invite you to submit a revised version of the manuscript that addresses the points raised during the review process.

Please submit your revised manuscript by Jul 19 2026 11:59PM. If you will need more time than this to complete your revisions, please reply to this message or contact the journal office at plosone@plos.org. When you're ready to submit your revision, log on to https://www.editorialmanager.com/pone/ and select the 'Submissions Needing Revision' folder to locate your manuscript file.

- A letter that responds to each point raised by the academic editor and reviewer(s). You should upload this letter as a separate file labeled 'Response to Reviewers'.
- A marked-up copy of your manuscript that highlights changes made to the original version. You should upload this as a separate file labeled 'Revised Manuscript with Track Changes'.
- An unmarked version of your revised paper without tracked changes. You should upload this as a separate file labeled 'Manuscript'.

If you would like to make changes to your financial disclosure, please include your updated statement in your cover letter. Guidelines for resubmitting your figure files are available below the reviewer comments at the end of this letter.

If applicable, we recommend that you deposit your laboratory protocols in protocols.io to enhance the reproducibility of your results. Protocols.io assigns your protocol its own identifier (DOI) so that it can be cited independently in the future. For instructions see: https://journals.plos.org/plosone/s/submission-guidelines#loc-laboratory-protocols. Additionally, PLOS ONE offers an option for publishing peer-reviewed Lab Protocol articles, which describe protocols hosted on protocols.io. Read more information on sharing protocols at https://plos.org/protocols?utm_medium=editorial-email&utm_source=authorletters&utm_campaign=protocols.

As the corresponding author, your ORCID iD is verified in the submission system and will appear in the published article. PLOS supports the use of ORCID, and we encourage all coauthors to register for an ORCID iD and use it as well. Please encourage your coauthors to verify their ORCID iD within the submission system before final acceptance, as unverified ORCID iDs will not appear in the published article. *Only*  the individual author can complete the verification step; PLOS staff *cannot*  verify ORCID iDs on behalf of authors.

We look forward to receiving your revised manuscript.

Kind regards,

Gennady S. Cymbalyuk, Ph.D.

Academic Editor

PLOS One

Journal Requirements:

When submitting your revision, we need you to address these additional requirements.

1. Please ensure that your manuscript meets PLOS ONE's style requirements, including those for file naming. The PLOS ONE style templates can be found at

https://journals.plos.org/plosone/s/file?id=wjVg/PLOSOne_formatting_sample_main_body.pdf and https://journals.plos.org/plosone/s/file?id=ba62/PLOSOne_formatting_sample_title_authors_affiliations.pdf

2. Thank you for stating the following in the Acknowledgments Section of your manuscript:

This work is partially supported by Young Scientific and Technological Talents Program for Higher Education Institutions of Inner Mongolia (NJYT24061); Fundamental Research Funds Program for Colleges and Universities Directly Affiliated to Inner Mongolia Autonomous Region (JY20220249). The funding covered research activities but did not provide support for open access publication fees.

We note that you have provided funding information that is not currently declared in your Funding Statement. However, funding information should not appear in the Acknowledgments section or other areas of your manuscript. We will only publish funding information present in the Funding Statement section of the online submission form.

Please remove any funding-related text from the manuscript and let us know how you would like to update your Funding Statement. Currently, your Funding Statement reads as follows:

This work is partially supported by Young Scientific and Technological Talents Program for Higher Education Institutions of Inner Mongolia (NJYT24061); Fundamental Research Funds Program for Colleges and Universities Directly Affiliated to Inner Mongolia Autonomous Region (JY20220249). The funding covered research activities but did not provide support for open access publication fees.

Please include your amended statements within your cover letter; we will change the online submission form on your behalf.

3. We note that Figure(s) 1, 2, 9 in your submission contain copyrighted images. All PLOS content is published under the Creative Commons Attribution License (CC BY 4.0), which means that the manuscript, images, and Supporting Information files will be freely available online, and any third party is permitted to access, download, copy, distribute, and use these materials in any way, even commercially, with proper attribution. For more information, see our copyright guidelines: http://journals.plos.org/plosone/s/licenses-and-copyright.

We require you to either (1) present written permission from the copyright holder to publish these figures specifically under the CC BY 4.0 license, or (2) remove the figures from your submission:

a. You may seek permission from the original copyright holder of Figure(s) 1, 2, 9 to publish the content specifically under the CC BY 4.0 license.

We recommend that you contact the original copyright holder with the Content Permission Form (http://journals.plos.org/plosone/s/file?id=7c09/content-permission-form.pdf) and the following text:

“I request permission for the open-access journal PLOS ONE to publish XXX under the Creative Commons Attribution License (CCAL) CC BY 4.0 (http://creativecommons.org/licenses/by/4.0/). Please be aware that this license allows unrestricted use and distribution, even commercially, by third parties. Please reply and provide explicit written permission to publish XXX under a CC BY license and complete the attached form.”

Please upload the completed Content Permission Form or other proof of granted permissions as an "Other" file with your submission.

In the figure caption of the copyrighted figure, please include the following text: “Reprinted from [ref] under a CC BY license, with permission from [name of publisher], original copyright [original copyright year].”

b. If you are unable to obtain permission from the original copyright holder to publish these figures under the CC BY 4.0 license or if the copyright holder’s requirements are incompatible with the CC BY 4.0 license, please either i) remove the figure or ii) supply a replacement figure that complies with the CC BY 4.0 license. Please check copyright information on all replacement figures and update the figure caption with source information. If applicable, please specify in the figure caption text when a figure is similar but not identical to the original image and is therefore for illustrative purposes only.

4. If the reviewer comments include a recommendation to cite specific previously published works, please review and evaluate these publications to determine whether they are relevant and should be cited. There is no requirement to cite these works unless the editor has indicated otherwise.

[Note: HTML markup is below. Please do not edit.]

Reviewers' comments:

Reviewer's Responses to Questions

**Comments to the Author**

1. Is the manuscript technically sound, and do the data support the conclusions?

Reviewer #1: Yes

Reviewer #2: Partly

**********

2. Has the statistical analysis been performed appropriately and rigorously?

Reviewer #1: Yes

Reviewer #2: No

**********

3. Have the authors made all data underlying the findings in their manuscript fully available?

The PLOS Data policy

Reviewer #1: Yes

Reviewer #2: No

**********

4. Is the manuscript presented in an intelligible fashion and written in standard English?

Reviewer #1: Yes

Reviewer #2: No

**********

Reviewer #1: This manuscript proposes a Hierarchy Aware Disentangled Diffusion (HADD) framework for monocular 3D human pose estimation, addressing well known challenges in diffusion based and disentangled approaches. By injecting noise into disentangled bone length and direction representations only in the forward diffusion process, and optimizing joint coordinates directly in the reverse process, the method effectively incorporates anatomical priors while avoiding hierarchical error accumulation.

A central contribution is the Hierarchical Spatial and Temporal Denoising module, which explicitly models parent–child dependencies in both spatial and temporal domains. The experimental evaluation on Human3.6M and MPI INF 3DHP is comprehensive, including comparisons with a wide range of baselines and detailed ablation studies. The results demonstrate consistent performance gains, particularly for high hierarchy joints. Overall, the study is technically rigorous and offers a well validated solution to core limitations in monocular 3D pose estimation.

There are a lot of typos, for example:

1. “ARSTRACT” should be ABSTRACT

2. In abstract: “which deeply integrate disentanglement strategy” should be “which deeply integrates the disentanglement strategy”

3. In introduction: “while avoid the hierarchical error accumulation” should be “while avoiding hierarchical error accumulation”

4. Fig 4 “Diffenrent Settings” should be “Different Settings”

Reviewer #2: The manuscript presents a 3D human pose estimation framework that combines a disentangled bone representation with diffusion-based denoising and explicit hierarchical modeling of the human skeleton. The proposed HSTD module is intended to model parent-child spatial and temporal dependencies, and the experiments include benchmark comparisons, ablations, robustness tests, qualitative visualizations, and efficiency measurements. The topic is within scope for PLOS ONE, and the manuscript contains a technically plausible core idea. However, substantial clarification and likely re-analysis are required before the results can be considered reliable.

Major comments

1. The Human3.6M train/test split appears to contain data leakage.

The experimental setup states that Human3.6M training uses subjects S1, S2, S3, S4, S5, S6, S7, S8, and S9, while testing uses S9 and S11. This places S9 in both the training and test sets. If this is a typographical error, please correct the text and provide the exact subject, camera, action, and frame sampling protocol used. If S9 was actually used in both training and testing, the Human3.6M results are not valid as a generalization test and should be rerun with a non-overlapping split. This point is central because the main claims on Human3.6M include improvements over disentanglement-based, deterministic, and probabilistic baselines.

2. The multi-hypothesis inference procedure is not validly specified.

The inference section states that after DDIM sampling, the method obtains H denoised 3D pose sequences and selects the final prediction as the one with the smallest MPJPE relative to the input 2D pose sequence, or by majority voting. MPJPE is a 3D error metric requiring ground-truth 3D pose; it cannot be computed “relative to” a 2D input sequence. If ground-truth MPJPE is used to choose among hypotheses, the reported performance would be oracle performance and not a valid deployable inference result. If the authors instead mean 2D reprojection error, please define the camera model, projection procedure, and selection rule precisely. Please report non-oracle results separately from any oracle upper bound.

3. Baseline comparisons require tighter control and clearer reporting.

The manuscript compares HADD against disentanglement-based, transformer-based, and diffusion-based methods, and reports favorable Human3.6M and MPI-INF-3DHP results. However, the comparisons mix different input assumptions, frame lengths, inference configurations, and auxiliary information. For example, the DiffPose comparison shows that HADD outperforms DiffPose-S under the same 2D-sequence-only setting, but the full DiffPose model with additional heatmaps and depth priors performs better than HADD. Therefore, broad “state-of-the-art” claims should be qualified carefully.

Please specify for every baseline whether the result was reproduced by the authors or copied from the literature, what 2D detector or ground-truth 2D input was used, whether the same preprocessing and evaluation scripts were used, and whether the same inference budget was allowed. The current manuscript reports means, standard deviations, and confidence intervals, but it does not adequately explain how these were computed or whether runs were paired across methods. This is important because some reported gains are small, for example the 0.5 mm MPI-INF-3DHP MPJPE improvement over UDM-HPE and the 0.2 mm P-MPJPE improvement over DiffPose-S.

4. The diffusion formulation needs to be made internally consistent.

The manuscript should clarify whether the denoising network predicts noise, the clean disentangled variables, or final 3D joint coordinates. The training description says the reverse process outputs denoised 3D joint coordinates, from which bone length and direction are recomputed. The inference description, however, refers to noise estimation terms for bone length and direction as core HSTD outputs. These are different diffusion parameterizations. Please rewrite this section so that the forward process, training target, reverse sampling equations, and regression head are mathematically consistent.

Related issues include inconsistent notation, undefined or inconsistent variables, and unclear variance/temperature definitions. The conditional distribution in the reverse process should specify whether conditioning is on the 2D pose sequence, noisy bone features, timestep, and any camera parameters. If the model predicts 3D pose directly rather than reconstructing it via forward kinematics, the role of the disentangled variables in inference should be described more explicitly.

5. The HSTD attention mechanisms need stronger justification and clearer tensor definitions.

The HSTD module is a reasonable idea, but the current presentation of HSDM and HTDM is difficult to verify. The manuscript states that HSDM amplifies parent-child attention weights using the skeletal tree, while HTDM models temporal dependencies between target and child joints. The HSDM attention update appears heuristic and should be justified more carefully. Please clarify whether attention is computed per frame, across all joints and frames, or in a flattened joint-frame space. The current reshaping notation risks mixing temporal and spatial attention in a way that contradicts the claimed “spatial” interpretation.

Similarly, the HTDM cross-attention branch should specify the query, key, and value tensors and how child-joint aggregation is handled for joints with zero, one, or multiple children. A diagram is helpful, but the equations must be sufficient for implementation. The HSDM equation that propagates attention from parent to child joints also needs a clearer derivation or ablation to show why this particular averaging rule is appropriate.

6. The ablation studies are useful but not yet sufficient.

The ablation on disentangled input versus disentangled output is one of the stronger parts of the paper, since it directly supports the design decision to use disentanglement in the forward diffusion process while avoiding forward-kinematic reconstruction in the reverse process. The reported results show that disentangled input improves performance, while disentangled output increases high-hierarchy errors.

However, several ablation effects are small. The module ablation reports improvements from hierarchical embedding, HSDM, and HTDM, but the incremental gains should be presented with numerical tables, confidence intervals, and significance tests rather than figures alone. Please also run the principal ablations on MPI-INF-3DHP or another generalization setting, not only Human3.6M, to show that the effect is not dataset-specific.

7. The hierarchical error analysis should include numerical values and clearer aggregation rules.

The hierarchical joint error analysis is directly tied to the manuscript’s main claim that HADD mitigates error accumulation in high-hierarchy joints. The figure suggests improved performance at terminal hierarchies, but the exact numerical errors, confidence intervals, and joint membership per hierarchy are not sufficiently reported. Please provide a table with MPJPE and P-MPJPE by hierarchy for all methods, define the aggregation rule over joints and frames, and resolve the inconsistency between N=231 in the hierarchy experiment and N=243 in the rest of the paper.

8. Robustness experiments should be reported more quantitatively and interpreted more conservatively.

The robustness section evaluates random coordinate zeroing and Gaussian noise in 2D inputs. This is a useful stress test, and the manuscript states that HADD degrades more slowly than baselines under both perturbations. However, setting 2D coordinates to zero is an artificial occlusion model and may not reflect detector failures, confidence-weighted missing keypoints, or real image occlusion. Please provide numerical tables with means and confidence intervals for Figures 7 and 8, and consider adding a more realistic perturbation protocol based on 2D detector confidence dropout or actual occlusion subsets. The discussion should avoid overclaiming “real-world” robustness unless validated under more realistic conditions.

9. Computational efficiency claims should be qualified.

The efficiency table reports HADD at 22.4 GFLOPs and 48.9 ms per frame. This is faster than several diffusion/probabilistic baselines listed, but it is slower and more computationally expensive than MixSTE, STCFormer, Anatomy3D, and Virtual Bones in the same table. Therefore, the claim should be limited to a favorable trade-off relative to diffusion-based methods, not to all SOTA methods. Please also report parameter count, training time, number of sampling steps, GPU hardware, batch size used during timing, and whether timing includes 2D pose detection.

10. Data availability and ethics statements need clarification.

The data availability statement says the study uses Human3.6M and MPI-INF-3DHP and provides a GitHub code link. Please ensure that the repository includes training and evaluation scripts, configuration files, exact preprocessing, random seeds, and instructions for reproducing every table. If pretrained checkpoints cannot be shared, this should be stated. Also, because the datasets involve human subjects and may be distributed under dataset-specific access agreements, the statement that all data are available “without restriction” should be checked for accuracy. The ethics statement should explain that the work uses publicly available/de-identified benchmark datasets and does not involve new human-subject data collection, rather than simply listing N/A.

11. The manuscript requires substantial editing for English, formatting, and notation.

There are numerous issues that interfere with technical clarity: the abstract heading is written as “ARSTRACT”; several sentences contain subject-verb and tense errors; equations and variables are inconsistently formatted; labels such as “Hierarchicy,” “P-MPJE,” and “Diffenrent” appear in figures; and some method names are inconsistent. These problems do not by themselves determine scientific validity, but they currently make the work difficult to evaluate. The manuscript should undergo careful technical editing before resubmission.

Minor comments

Please define all acronyms on first use and use consistent notation for MPJPE, P-MPJPE, PCK, AUC, HSTD, HSDM, HTDM, HSP, and TP.

Please avoid stating that bone direction loss enforces physiological joint-angle limits unless an explicit joint-angle constraint is actually used. An L2 loss on unit direction vectors encourages agreement with ground truth but does not by itself enforce anatomical range-of-motion limits.

Please state whether 3D poses are root-aligned, scale-normalized, camera-coordinate, or world-coordinate for every reported metric. The preprocessing description states that 3D coordinates are centered on the pelvis and scaled, which may affect comparison with standard benchmark protocols.

Please move URLs and footnotes out of tables where they disrupt formatting.

Please report the number of trainable parameters and model memory usage in addition to FLOPs and inference time.

Please make the limitations section more precise. The current manuscript acknowledges weaknesses under severe occlusion, multi-person interaction, unconstrained in-the-wild settings, and mesh/surface estimation. These are appropriate limitations, but they should be tied more directly to the evidence provided by the experiments.

**********

what does this mean?). If published, this will include your full peer review and any attached files.

If you choose “no”, your identity will remain anonymous but your review may still be made public.

**Do you want your identity to be public for this peer review?**  For information about this choice, including consent withdrawal, please see our Privacy Policy

Reviewer #1: No

Reviewer #2: No

**********

[NOTE: If reviewer comments were submitted as an attachment file, they will be attached to this email and accessible via the submission site. Please log into your account, locate the manuscript record, and check for the action link "View Attachments". If this link does not appear, there are no attachment files.]

To ensure your figures meet our technical requirements, please review our figure guidelines: https://journals.plos.org/plosone/s/figures

You may also use PLOS’s free figure tool, NAAS, to help you prepare publication quality figures: https://journals.plos.org/plosone/s/figures#loc-tools-for-figure-preparation.

NAAS will assess whether your figures meet our technical requirements by comparing each figure against our figure specifications.

# Author comment: Author response to Decision Letter 1

**DOI:** 10.1371/journal.pone.0359321.r002

1 Jul 2026

Please refer to the attachment "Response to reviewers" and "Response to editors" for all comments from the editor and reviewers.

Attachment Submitted filename: Response to reviewers.docx

# Peer review: Decision Letter 1

**DOI:** 10.1371/journal.pone.0359321.r003

**Authors:** Gennady S. Cymbalyuk

22 Jul 2026

Dear Dr. Jian,

plosone@plos.org. When you're ready to submit your revision, log on to https://www.editorialmanager.com/pone/ and select the 'Submissions Needing Revision' folder to locate your manuscript file.

- A letter that responds to each point raised by the academic editor and reviewer(s). You should upload this letter as a separate file labeled 'Response to Reviewers'.
- A marked-up copy of your manuscript that highlights changes made to the original version. You should upload this as a separate file labeled 'Revised Manuscript with Track Changes'.
- An unmarked version of your revised paper without tracked changes. You should upload this as a separate file labeled 'Manuscript'.

If you would like to make changes to your financial disclosure, please include your updated statement in your cover letter. Guidelines for resubmitting your figure files are available below the reviewer comments at the end of this letter.

If applicable, we recommend that you deposit your laboratory protocols in protocols.io to enhance the reproducibility of your results. Protocols.io assigns your protocol its own identifier (DOI) so that it can be cited independently in the future. For instructions see: https://journals.plos.org/plosone/s/submission-guidelines#loc-laboratory-protocols. Additionally, PLOS ONE offers an option for publishing peer-reviewed Lab Protocol articles, which describe protocols hosted on protocols.io. Read more information on sharing protocols at https://plos.org/protocols?utm_medium=editorial-email&utm_source=authorletters&utm_campaign=protocols.

As the corresponding author, your ORCID iD is verified in the submission system and will appear in the published article. PLOS supports the use of ORCID, and we encourage all coauthors to register for an ORCID iD and use it as well. Please encourage your coauthors to verify their ORCID iD within the submission system before final acceptance, as unverified ORCID iDs will not appear in the published article. *Only*  the individual author can complete the verification step; PLOS staff *cannot*  verify ORCID iDs on behalf of authors.

We look forward to receiving your revised manuscript.

Kind regards,

Gennady S. Cymbalyuk, Ph.D.

Academic Editor

PLOS One

Journal Requirements:

If the reviewer comments include a recommendation to cite specific previously published works, please review and evaluate these publications to determine whether they are relevant and should be cited. There is no requirement to cite these works unless the editor has indicated otherwise.

**Additional Editor Comments:**

The manuscript, equations, and linked implementation appear to describe different model parameterizations, including whether the final pose is predicted directly or reconstructed through forward kinematics. The dataset splits and multi-hypothesis selection procedure are also not verifiable from the available code, and the reported statistical analyses and confidence intervals require recalculation from the raw run-level results. In addition, the reverse-sampling formulation is incomplete, and the current repository does not substantiate the claim that all reported experiments can be reproduced.

These issues directly affect the identity of the implemented method, the validity of the evaluation protocol, and the reliability of the quantitative conclusions. They should therefore be fully addressed, with consistent code and manuscript descriptions, exact split manifests, corrected statistical analyses, a complete sampling algorithm, and a frozen reproducible release, before the manuscript can be considered for acceptance.

[Note: HTML markup is below. Please do not edit.]

Reviewers' comments:

Reviewer's Responses to Questions

**Comments to the Author**

Reviewer #1: All comments have been addressed

Reviewer #2: (No Response)

**********

2. Is the manuscript technically sound, and do the data support the conclusions?

Reviewer #1: Yes

Reviewer #2: Partly

**********

3. Has the statistical analysis been performed appropriately and rigorously?

Reviewer #1: Yes

Reviewer #2: No

**********

4. Have the authors made all data underlying the findings in their manuscript fully available?

The PLOS Data policy

Reviewer #1: Yes

Reviewer #2: No

**********

5. Is the manuscript presented in an intelligible fashion and written in standard English?

Reviewer #1: Yes

Reviewer #2: No

**********

Reviewer #1: The authors have adequately addressed my comments.

The authors have adequately addressed my comments.

Reviewer #2: Thank you for the extensive revision! The manuscript has improved substantially. In particular, the intended Human3.6M split and camera model are now stated, the HSTD tensor definitions have been expanded, numerical hierarchy-level and robustness results have been added, principal ablations have been extended to MPI-INF-3DHP, efficiency and state-of-the-art claims have been narrowed, and the ethics and limitations statements are more appropriate.

Nevertheless, I cannot recommend acceptance jut yet because several central technical and reproducibility issues remain unresolved.

1. The linked implementation and revised manuscript describe different model parameterizations. The manuscript states that HSTD plus the regression head directly predicts clean 3D joint coordinates and that the final pose is not reconstructed through forward kinematics. In the linked implementation, however, HSTD returns a four-channel tensor identified as a noise prediction, and both training and inference reconstruct bone length, bone direction, and the resulting 3D pose using the disentanglement/reconstruction module. This is not a minor code difference: avoiding forward-kinematic error accumulation is one of the paper’s central claims. The authors must provide the exact frozen implementation used for the reported experiments and make the manuscript, equations, and code agree. If the currently linked code generated the results, the method description and associated mechanistic claims must be revised. If different code generated the results, that exact code must be released.

2. The dataset split and hypothesis-selection protocol remain unverifiable. The revised manuscript states that Human3.6M uses S1, S5, S6, S7, and S8 for training and S9 and S11 for testing. However, the repository contains two incompatible split implementations, neither of which directly matches this protocol. The authors should provide exact machine-readable manifests listing every subject, camera, action, and sampled sequence used in training, validation, and testing. Any affected Human3.6M experiments must be rerun.

The revised manuscript also states that multi-hypothesis selection uses calibrated projection based on K,R,g. The linked inference code appears to call a projection routine without supplying these parameters, resulting in the simplified mapping x/z,y/z. The authors should provide the exact hypothesis-selection code used for all reported tables and demonstrate that it uses the stated calibration parameters and no ground-truth 3D information.

3. The statistical results must be recalculated. The reported confidence intervals do not follow the manuscript’s stated n=5 t-distribution formula. For example, 40.10±1.12 would imply a 95% confidence interval of approximately [38.71; 41.49], rather than the reported [39.71; 40.49]. Comparable discrepancies occur in other tables. The authors should provide the five raw values for every method and configuration, clarify exactly what each reported standard deviation represents, recalculate all confidence intervals, and perform the claimed paired comparisons using paired run-level differences. Statements of statistical significance should be removed unless supported by appropriate test statistics and results.

4. The reverse-sampling formulation remains incomplete. Equation 9 is not an operational DDIM equation as printed because its coefficients are not multiplied by the predicted clean sample and noise-estimate terms. The reverse distribution in Equation 7 also mixes camera parameters used only for hypothesis evaluation with the conditional denoising distribution. The complete sampling algorithm should be provided mathematically and in pseudocode, with every tensor and timestep transition defined.

5. The reproducibility claim should be verified rather than asserted. The response states that the repository permits one-click reproduction of every table, but the presently linked repository does not substantiate this. A revision should include a frozen commit or archival release, environment specification, complete configurations for both datasets and all inference settings, exact preprocessing and evaluation scripts, split manifests, fixed seeds, and commands for reproducing every main and ablation table. Raw per-run results should also be deposited.

These issues concern the identity of the implemented method, the evaluation protocol, and the validity of the quantitative evidence. They should therefore be resolved before the manuscript is accepted. I recommend and encourage another major revision. Once the comments above are addressed, I will recommend for publication.

**********

what does this mean?). If published, this will include your full peer review and any attached files.

If you choose “no”, your identity will remain anonymous but your review may still be made public.

**Do you want your identity to be public for this peer review?**  For information about this choice, including consent withdrawal, please see our Privacy Policy

Reviewer #1: No

Reviewer #2: No

**********

[NOTE: If reviewer comments were submitted as an attachment file, they will be attached to this email and accessible via the submission site. Please log into your account, locate the manuscript record, and check for the action link "View Attachments". If this link does not appear, there are no attachment files.]

To ensure your figures meet our technical requirements, please review our figure guidelines: https://journals.plos.org/plosone/s/figures

You may also use PLOS’s free figure tool, NAAS, to help you prepare publication quality figures: https://journals.plos.org/plosone/s/figures#loc-tools-for-figure-preparation.

NAAS will assess whether your figures meet our technical requirements by comparing each figure against our figure specifications.

# Author comment: Author response to Decision Letter 2

**DOI:** 10.1371/journal.pone.0359321.r004

19 Aug 2026

Responses to the reviewers' comments are provided in the attachment.

Attachment Submitted filename: Response_to_reviewers_auresp_2.docx

# Peer review: Decision Letter 2

**DOI:** 10.1371/journal.pone.0359321.r005

**Authors:** Gennady S. Cymbalyuk

14 Sep 2026

HADD: A Hierarchy-Aware Disentangled Diffusion Framework with Spatio-Temporal Denoising for 3D Human Pose Estimation

PONE-D-26-14412R2

Dear Dr. Jian,

We’re pleased to inform you that your manuscript has been judged scientifically suitable for publication and will be formally accepted for publication once it meets all outstanding technical requirements.

Within one week, you’ll receive an e-mail detailing the required amendments. When these have been addressed, you’ll receive a formal acceptance letter and your manuscript will be scheduled for publication.

An invoice will be generated when your article is formally accepted. Please note, if your institution has a publishing partnership with PLOS and your article meets the relevant criteria, all or part of your publication costs will be covered. Please make sure your user information is up-to-date by logging into Editorial Manager at Editorial Manager® and clicking the ‘Update My Information' link at the top of the page. For questions related to billing, please contact billing support.

If your institution or institutions have a press office, please notify them about your upcoming paper to help maximize its impact. If they’ll be preparing press materials, please inform our press team as soon as possible -- no later than 48 hours after receiving the formal acceptance. Your manuscript will remain under strict press embargo until 2 pm Eastern Time on the date of publication. For more information, please contact onepress@plos.org.

Kind regards,

Gennady S. Cymbalyuk, Ph.D.

Academic Editor

PLOS One

Additional Editor Comments (optional):

Reviewers' comments:

Reviewer's Responses to Questions

**Comments to the Author**

Reviewer #1: All comments have been addressed

Reviewer #2: All comments have been addressed

**********

2. Is the manuscript technically sound, and do the data support the conclusions?

Reviewer #1: Yes

Reviewer #2: Yes

**********

3. Has the statistical analysis been performed appropriately and rigorously?

Reviewer #1: Yes

Reviewer #2: Yes

**********

4. Have the authors made all data underlying the findings in their manuscript fully available?

The PLOS Data policy

Reviewer #1: Yes

Reviewer #2: Yes

**********

5. Is the manuscript presented in an intelligible fashion and written in standard English?

Reviewer #1: Yes

Reviewer #2: Yes

**********

Reviewer #1: All comments have been addressed.

All comments have been addressed.

All comments have been addressed.

All comments have been addressed.

Reviewer #2: Thank you for addressing most of my previous concerns. I appreciate the revisions and the authors’ responses, which have clarified the main issues I raised in my previous review.

**********

what does this mean?). If published, this will include your full peer review and any attached files.

If you choose “no”, your identity will remain anonymous but your review may still be made public.

**Do you want your identity to be public for this peer review?**  For information about this choice, including consent withdrawal, please see our Privacy Policy

Reviewer #1: No

Reviewer #2: No

**********

# Editor report: Acceptance letter

**DOI:** 10.1371/journal.pone.0359321.r006

**Authors:** Gennady S. Cymbalyuk

PONE-D-26-14412R2

PLOS One

Dear Dr. Jian,

I'm pleased to inform you that your manuscript has been deemed suitable for publication in PLOS One. Congratulations! Your manuscript is now being handed over to our production team.

At this stage, our production department will prepare your paper for publication. This includes ensuring the following:

* All references, tables, and figures are properly cited

* All relevant supporting information is included in the manuscript submission,

* There are no issues that prevent the paper from being properly typeset

You will receive further instructions from the production team, including instructions on how to review your proof when it is ready. Please keep in mind that we are working through a large volume of accepted articles, so please give us a few days to review your paper and let you know the next and final steps.

Lastly, if your institution or institutions have a press office, please let them know about your upcoming paper now to help maximize its impact. If they'll be preparing press materials, please inform our press team within the next 48 hours. Your manuscript will remain under strict press embargo until 2 pm Eastern Time on the date of publication. For more information, please contact onepress@plos.org.

You will receive an invoice from PLOS for your publication fee after your manuscript has reached the completed accept phase. If you receive an email requesting payment before acceptance or for any other service, this may be a phishing scheme. Learn how to identify phishing emails and protect your accounts at https://explore.plos.org/phishing.

If we can help with anything else, please email us at customercare@plos.org.

Thank you for submitting your work to PLOS One and supporting open access.

Kind regards,

PLOS One Editorial Office Staff

on behalf of

Dr. Gennady S. Cymbalyuk

Academic Editor

PLOS One
